# Global quality of care trends for lip and oral cavity cancer: A quality of care index analysis, 1990–2021

**DOI:** 10.1016/j.pmedr.2026.103380

**Published:** 2026-01-07

**Authors:** Yilin Zhang, Wenkai Xu, Haocheng Wang, Liang Mao, Jing Sun

**Affiliations:** a School of Stomatology, Shandong Second Medical University, Weifang 261053, Shandong Province, People's Republic of China; b Zibo Municipal Government Hospital, No. 24, West Renmin Road, Zhangdian District, Zibo City, Shandong Province, 255000, People's Republic of China; c Peking University Medical Zibo Hospital; No. 2, Fifth Street, Western Hills, Zhangdian District, Zibo, Shandong Province, 255000, People's Republic of China

**Keywords:** Lip and oral cavity cancer, Quality of care index, Epidemiology, Disease burden, Public health

## Abstract

**Objective:**

To evaluate global trends and disparities in the quality of care for lip and oral cavity cancer (LOCC) from 1990 to 2021 using the Quality of Care Index (QCI).

**Methods:**

Data on LOCC burden were obtained from the Global Burden of Disease (GBD) 2021 study, covering 204 countries and territories from 1990 to 2021. The QCI was constructed via principal component analysis of four component indices: mortality-to-incidence ratio, prevalence-to-incidence ratio, years of life lost to years lived with disability ratio, and disability-adjusted life years to prevalence ratio. Analyses were stratified by socio-demographic index (SDI), age, and sex.

**Results:**

A geographical decoupling was observed: high-SDI regions had the highest LOCC incidence, while low-middle SDI regions had the highest mortality. The global QCI improved from 1990 to 2021 but strongly correlated with national development. Females consistently had higher QCI scores than males across all SDI groups. The QCI-age relationship varied by SDI region.

**Conclusions:**

Despite global improvement, significant inequities in LOCC care quality persist across regions, ages, and sexes. Future strategies must focus on enhancing healthcare system performance and ensuring equitable access to effective care.

## Highlights

• Global oral cancer care quality improved from 1990 to 2021.
• High incidence in developed regions, high mortality in low-resource areas.
• Females consistently had higher care quality scores than males.
• Care quality varied by age and socioeconomic development level.
• Strong correlation between national development and care quality.

## 1 Introduction

LOCC represents a significant global public health challenge(GBD 2017 Oral Disorders Collaborators and Bernabe, 2020). As the most common subtype of head and neck cancers, it is associated with substantial morbidity, mortality, and detrimental impacts on essential functions such as speech and swallowing(Chen et al., 2025). According to the GBD studies, LOCC affected over 1.54 million people worldwide in 2021(Alves-Costa et al., 2025). The burden exhibits notable disparities linked to geographic region, socioeconomic status, and healthcare access(Fan et al., 2022), compounded by risk factors like tobacco and alcohol use(Dai et al., 2025).

Extensive epidemiological studies and cancer registries have detailed LOCC incidence and mortality(GBD, 2023 Cancer Collaborators and, 2025). However, these metrics primarily reflect disease burden rather than healthcare system performance in prevention and treatment(Pan et al., 2025). A critical gap remains in quantitatively assessing the quality of LOCC care globally(Bi et al., 2025).

To bridge this gap, the QCI, a composite metric derived from GBD data, has been developed(Luo et al., 2025). The QCI synthesizes several key health metrics into a single, interpretable score, enabling a standardized, comparative assessment of healthcare quality across different geographies and over time(Xu et al., 2025). The longitudinal data (1990–2021) are crucial for evaluating trends in care quality, which evolve slowly with health system development. The GBD 2021 estimates remain the most recent comprehensive global data, ensuring contemporary relevance for policy. Therefore, this study aims to assess global and regional trends in the quality of care for LOCC from 1990 to 2021 using the QCI, examine disparities by SDI, age, and sex, and identify policy-relevant patterns that may inform equitable cancer control strategies.

## 2 Methods

### 2.1 Study design and population

Ethical statement: This study used publicly available, anonymized data from the GBD 2021 study and was exempt from ethical review approval at our institution.

The Global Burden of Disease 2021 study offers a comprehensive evaluation of the health impacts of 371 diseases and injuries, encompassing 204 countries and territories(GBD 2021 Diseases and Injuries Collaborators, 2024). Within the framework of this study, LOCC was delineated by the ICD-10 codes C00 through C08(Yu et al., 2024). All data, models, and methods are available via the GBD 2021 repository: https://ghdx.healthdata.org/gbd-2021/sources.

Our analysis was based on LOCC data from the GBD study (1990–2021), encompassing 204 countries and territories. The metrics extracted included incidence, mortality, prevalence, years of life lost (YLLs), years lived with disability (YLDs), and disability-adjusted life years (DALYs), alongside demographic variables(GBD 2021 Diseases and Injuries Collaborators, 2024). The SDI, a composite indicator of a country's development level (ranging from 0 to 1), was used for stratification(GBD 2019 Healthcare Access and Quality Collaborators, 2022). According to the GBD framework, study units were grouped into five SDI regions. Data for individuals under 15 years of age were excluded at the point of extraction from the GBD repository; therefore, all subsequent analyses, including the calculation of component indices and the PCA, were based solely on the population aged 15 years and older.(Alves-Costa et al., 2025).

### 2.2 Measures

The incidence and deaths of LOCC globally and across the five SDI regions in 2021 were summarized and compared.

This study employed four key ratios derived from GBD data to indirectly assess the quality of care for LOCC(Ghamari et al., 2022). The Years of life lost to Years lived with disability Ratio (YLR) distinguishes between diseases causing primarily premature death (high YLR) and those leading predominantly to disability (low YLR)(Azadnajafabad et al., 2023). The Mortality to Incidence Ratio (MIR) directly reflects the lethality of the disease and the effectiveness of medical interventions, with a lower MIR signifying more effective care(Choi et al., 2017). The Disability-adjusted life years to Prevalence Ratio (DPR) evaluates the severity of the disease at the population level; a high DPR indicates that even with low prevalence, the disease imposes a severe health burden. Lastly, the Prevalence to Incidence Ratio (PIR) sheds light on the disease duration, where a high PIR is characteristic of chronic conditions, and a low PIR suggests an acute disease process(Sofi-Mahmudi et al., 2021). These indices were calculated from the GBD estimates as follows: MIR = Deaths / Incidence; PIR = Prevalence / Incidence; YLR = YLLs / YLDs; DPR = DALYs / Prevalence(Ghafouri et al., 2023).

### 2.3 Statistical analysis

Data preprocessing was conducted to ensure robustness. Instances with zero denominators in the calculation of these ratios were handled by setting the resulting ratio to zero. Subsequently, all calculated values for MIR, PIR, YLR, and DPR were checked for infinite, missing, or extreme outlier values; all such instances were recoded to zero.(Ringnér, 2008). Prior to dimensionality reduction, the four component indices were standardized using *Z*-score normalization (mean-centered and scaled to unit variance) to account for differences in their scales and variances. Principal Component Analysis (PCA) was then performed on the standardized matrix. The PCA calculation was implemented using the prcomp function from the R stats package, while the factoextra package was employed for the visualization of PCA results. The first two principal components (PC1 and PC2) were retained for the construction of the composite index.(Pan et al., 2025). The final QCI score for each observation was derived in two steps. First, a weighted composite score was calculated from PC1 and PC2, with weights proportional to the variance explained by each component. This composite score was then linearly rescaled to a range of 0 to 100, where a higher score indicates better quality of care. All statistical analyses and visualizations were performed using R software (version 4.3.1).

## 3 Results

### 3.1 The global burden of lip and oral cavity cancer in 2021 and trends since 1990

**Fig. 1:**
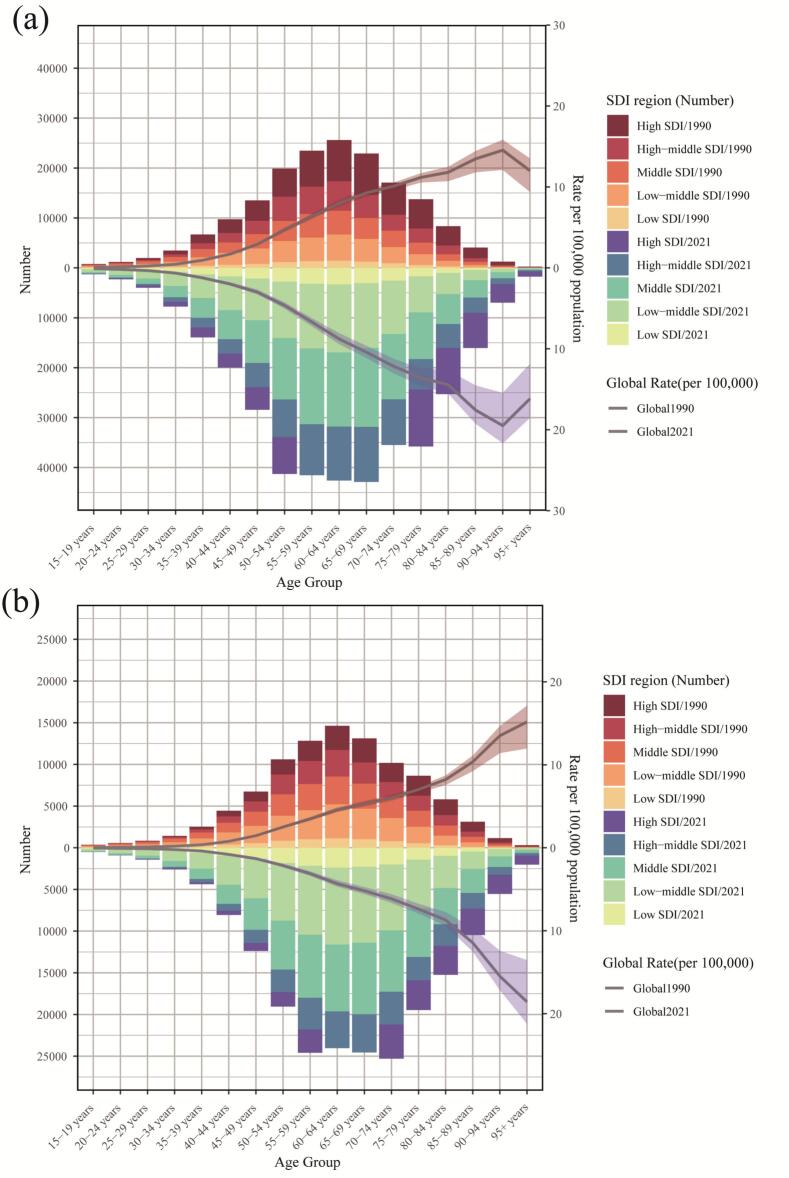
Age-specific incidence (A) and mortality (B) of lip and oral cavity cancer among individuals aged 15 years and older, stratified by Socio-demographic Index region, 1990 and 2021.

This study presents a comprehensive analysis of the burden of LOCC from 1990 to 2021, focusing on incidence and mortality across the SDI spectrum (Fig. 1 and Supplemental Table 1). The incidence of LOCC was highest in high-SDI regions, however, a critical divergence was observed in mortality patterns, as the highest number of deaths was concentrated in low-middle SDI regions, significantly surpassing those in high-SDI areas. This discrepancy underscores a stark disparity in healthcare outcomes, suggesting that while high-SDI regions detect more cases, low-middle SDI regions face greater challenges in delivering effective treatment, leading to poorer survival. From 1990 to 2021, the absolute number of both new cases and deaths from LOCC increased globally across most SDI levels and age groups. A marked rise in incident cases was documented; for instance, in the middle-high SDI region, cases in the 60–64 age group increased from 5943.89(95%UIs,5649.86265.4) to 10,807.5(95%UIs,9817.611765.8). Mortality exhibited a similarly concerning upward trend, particularly in low-middle SDI regions, where deaths in the 55–59 age group surged from 3494.9 to 8276.3(95%UIs,7146.69468), representing one of the most pronounced increases. In contrast, some high-SDI regions showed stable or even declining mortality in younger age groups. The age distribution of LOCC burden demonstrated a consistent pattern, with both incidence and mortality remaining low in younger ages (15–49 years), rising precipitously through middle age, and peaking between 60 and 70 years before declining in the oldest old (80+ years). This pattern aligns with the typical carcinogenesis process of LOCC, with the peak mortality burden occurring slightly earlier, in the 55-69 year cohort, within low-middle SDI regions.

### 3.2 QCI trends in various countries around the world

**Fig. 2:**
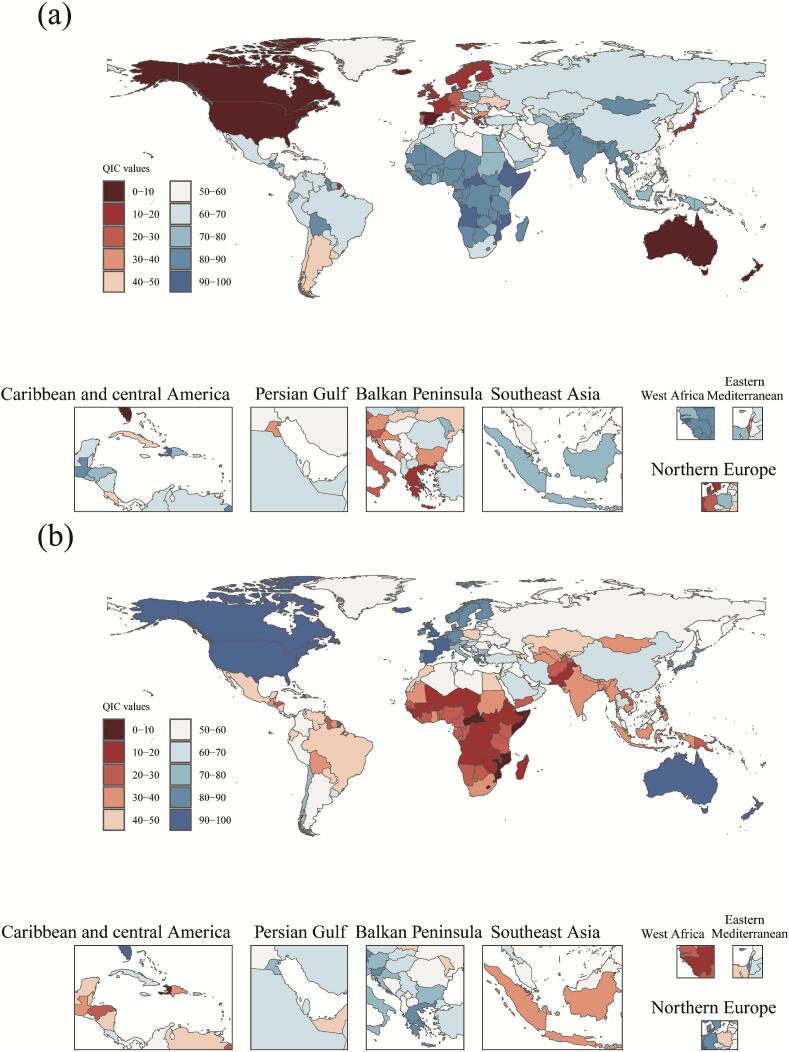
Global distribution of the Quality of Care Index for lip and oral cavity cancer among individuals aged 15 years and older, 2021.

The QCI assessment revealed a global distribution across a spectrum of performance levels Fig. 2 and Supplemental Table 2). Countries with QCI scores of 90 or above, such as Australia, Spain, and the United States, represented the highest tier. This was followed by countries scoring between 80 and 89, including Switzerland, Norway, Japan, and South Korea. A tier of scores from 60 to 79 included nations like Italy, Singapore, and China (67.1 points,95%UIs:60**,**70), while scores in the 40–59 range were observed in countries such as Russia and Brazil (47.2 points，95%UIs:40**,**50). Lower performance tiers included scores of 20–39 (e.g., India at 34.8 points,95%UIs:30**,**40), 10–19 (e.g., Pakistan, Ethiopia), and below 10, with the lowest scores found in Haiti, Somalia, and the Central African Republic.

Geographically, higher QCI scores were concentrated in Oceania, Western Europe, and North America, whereas lower scores were predominantly clustered in Sub-Saharan Africa and South Asia. The index demonstrated a positive association with national economic development levels. However, some countries, including Cuba (61.9 points,95%UIs:60**,**70) and Costa Rica (62.4 points, 95%UIs:60**,**70), achieved scores notably higher than their economic peers. Regional disparities were evident within Asia, with Japan and South Korea attaining high scores, contrasting with the significant gap between Singapore (79.7 points,95%UIs:70**,**80) and Cambodia (32.76 points,95%UIs:30**,**40). European countries consistently demonstrated strong performance, featuring prominently among the higher-scoring nations.

### 3.3 QCI situation of each age group

**Fig. 3:**
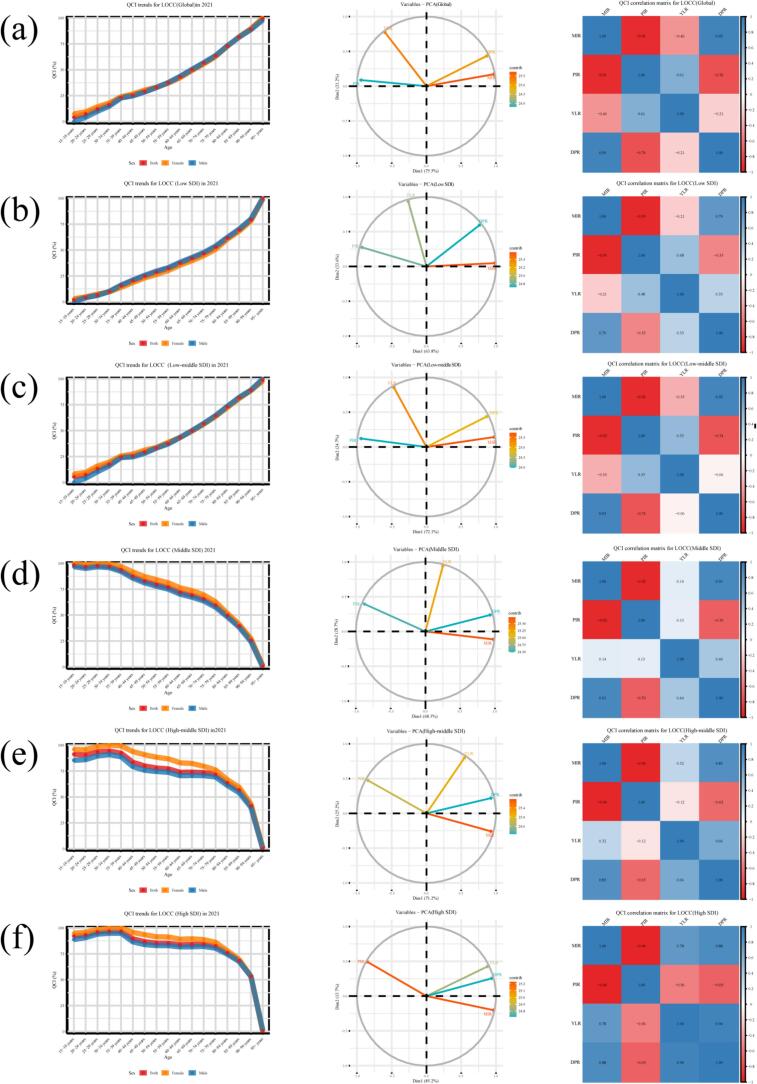
Age- and sex-specific trends in the Quality of Care Index for lip and oral cavity cancer and its correlation with component indices among individuals aged 15 years and older, stratified by Socio-demographic Index region, 2021. (Component indices: Years of Life Lost to Years Lived with Disability Ratio, Disability-Adjusted Life Years to Prevalence Ratio, Mortality-to-Incidence Ratio, Prevalence-to-Incidence Ratio).

Globally, the QCI exhibited a consistent positive correlation with age, increasing from a mean of 1.8(95%UIs: 0**,**10) in the 15–19 age group to 99.7(95%UIs:90**,**100) in the 95+ age group, with females generally showing marginally higher QCI values than males. Principal component analysis revealed that the PC1 accounted for 75.5% of the total variance (Fig. 3 and Supplemental Table 3). Analysis across SDI regions revealed distinct patterns. In Low and Low-Middle SDI regions, QCI demonstrated a monotonic increase with advancing age, rising from 0 to 7.5(95%UIs:0**,**10) to 98.1–100(95%UIs:90**,**100) and from approximately 5.3(95%UIs:0**,**10) to 98.8(95%UIs:90**,**100), respectively; females consistently had higher QCI values, and PC1 explained a substantial portion of the variance (63.8% and 72.1%, respectively) (Fig. 3b and c and Supplemental Table3). Conversely, in the Middle SDI region, QCI declined with age, from 98.4(95%UIs:90**,**100) in the youngest group to 0.7(95%UIs:0**,**10) in the oldest, with higher female QCI, while PC1 explained 68.3% of the variance (Fig. 3d and Supplemental Table 3). An inverted U-shaped trajectory was observed in High-Middle and High SDI regions, where QCI initially rose to a peak in middle age (e.g., 100(95%UIs:90**,**100) in females aged 30–34 and 35–39, respectively) before sharply declining to near zero in the oldest groups; females maintained a QCI advantage, and the explanatory power of PC1 was 71.2% and 85.2%, respectively (Fig. 3e and f and Supplemental Table 3).

Analysis of correlations among MIR, PIR, YLR, and DPR revealed that (Fig. 3): In the Low SDI region, MIR showed a strong positive correlation with DPR. In the Low-Middle SDI region, MIR also demonstrated strong positive correlations with YLR and DPR (approximately 0.8–0.9), while PIR was negatively correlated with these indicators. In the Middle SDI region, MIR and DPR increased with age and were negatively correlated with QCI, whereas PIR showed a positive correlation trend with QCI. In the High-Middle and High SDI regions, QCI exhibited strong negative correlations with MIR, YLR, and DPR, and a strong positive correlation with PIR.

### 3.4 QCI situation of each sex group

**Fig. 4:**
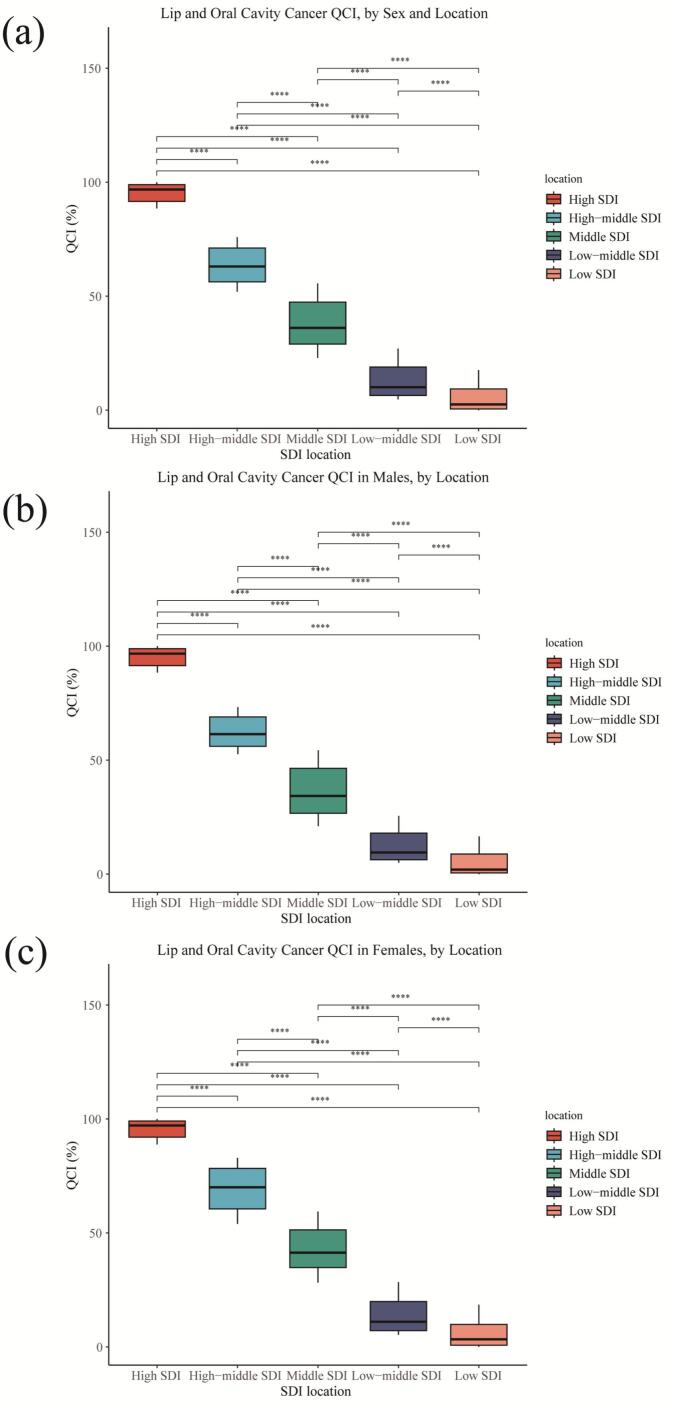
Univariate analysis of Quality of Care Index scores for lip and oral cavity cancer among individuals aged 15 years and older, stratified by sex and Socio-demographic Index group, 2021.

Between 1990 and 2021, the QCI showed universal improvement across all SDI regions for the combined data of both sexes. The high SDI region maintained the highest QCI, increasing from 88.5(95%UIs:80**,**90) to 100(95%UIs:90**,**100), while the middle SDI region demonstrated the most significant growth, surging by 143% from 22.9(95%UIs:20**,**30) to 55.6(95%UIs:50**,**60) (Fig. 4a and Supplemental Table 4). Analysis by gender also revealed a consistent upward trend. Among males, QCI increased from 88.4(95%UIs:80**,**90) to 100(95%UIs:90**,**100) in the high SDI region, from 52.6(95%UIs:50**,**60) to 73.3(95%UIs:70**,**80) in the high-middle SDI region, from 21(95%UIs:20**,**30) to 54.4(95%UIs:50**,**60) in the middle SDI region, from 4.9(95%UIs:0**,**10) to 25.5(95%UIs:20**,**30) in the low-middle SDI region, and from 0.6(95%UIs:0**,**10) to 16.5(95%UIs:10**,**20) in the low SDI region (Fig. 4b and Supplemental Table 4). Similarly, among females, QCI rose from 88.9(95%UIs:80**,**90) to 100(95%UIs:90**,**100) in the high SDI region, from 53.9(95%UIs:50**,**60) to 83(95%UIs:80**,**90) in the high-middle SDI region, from 28.2(95%UIs:20**,**30) to 59.4(95%UIs:50**,**60) in the middle SDI region, from 5.3(95%UIs:0**,**10) to 28.4(95%UIs:20**,**30) in the low-middle SDI region, and from 0.27(95%UIs:0**,**10) to 18.5(95%UIs:10**,**20) in the low SDI region (Fig. 4c and Supplemental Table 4). Throughout the study period, females consistently showed higher QCI values than males across all SDI regions, which aligns with our previous analysis.

## 4 Discussion

This study, based on global data on the burden and quality of care for oral cancer from 1990 to 2021, systematically reveals its multidimensional characteristics in terms of incidence, mortality, and the QCI. The main findings present a complex and uneven global picture. These persistent disparities highlight priorities that remain urgent today, such as strengthening early detection in low-resource settings. The findings thus provide directly actionable evidence for current efforts to achieve equitable cancer care.

Regarding disease burden, a notable “geographic decoupling of incidence and mortality” was observed: while high-SDI regions reported the highest incidence rates, low- and low-middle SDI regions bore the heaviest mortality burden(Meng et al., 2025). This contrast highlights structural inequities in early diagnosis and treatment accessibility for oral cancer globally. More comprehensive screening systems and diagnostic capabilities in high-SDI regions drive higher registered incidence, whereas low- and middle-low SDI regions suffer from high mortality due to scarce medical resources and delayed treatment, particularly evident among the core working-age population(Wu et al., 2025a, Wu et al., 2025b). This highlights the urgent need for targeted interventions in resource-limited settings. For example, strengthening community-based patient education to improve recognition of early oral symptoms such as persistent ulcers, lumps, or red or white patches could encourage earlier care seeking. Training primary healthcare workers in basic oral examination and referral protocols may also help bridge the diagnostic gap where specialist access is limited. (Liu and Han, 2025).

The global distribution of QCI further confirms the strong correlation between healthcare resources and economic development levels. High-scoring countries are concentrated in North America, Western Europe, and Oceania, while low-scoring countries cluster in Sub-Saharan Africa and South Asia. Countries like Cuba and Costa Rica achieved QCI scores significantly surpassing their SDI counterparts. This suggests that targeted health policies and strategic resource allocation can partially mitigate economic constraints(Benzian et al., 2022).Cuba's achievement may be attributed to its deeply embedded, community-oriented primary healthcare network, which could facilitate early detection and patient navigation(Dresang et al., 2005). Costa Rica's performance might reflect the success of its robust public health system and sustained tobacco control policies(Crosbie et al., 2016). These examples emphasize that beyond gross national income, governance, political commitment to health equity, and the design of service delivery models are pivotal determinants of cancer care quality. China's QCI score places it in the middle range, above most developing countries but still showing a noticeable gap compared to high-performing nations, reflecting room for improvement in its specialized oral cancer prevention and treatment system(Wu et al., 2025a, Wu et al., 2025b).

The relationship between QCI and age exhibited distinct patterns stratified by SDI levels. In low and low-middle SDI regions, QCI increased monotonically with age, suggesting that limited resources might be channeled more toward older patients with advanced disease, representing a passive, reactive approach rather than proactive prevention(Yang et al., 2025a, Yang et al., 2025b). In contrast, in high-middle and high SDI regions, QCI showed an inverted U-shaped relationship with age, with a sharp decline in the oldest groups (especially those over 80), hinting at potential age-based undertreatment or clinical bias, where even in resource-rich settings, older patients may face barriers to receiving aggressive therapy(Pan et al., 2025).

Females consistently demonstrated a QCI advantage across all SDI regions and age groups. This disparity may stem from behavioral patterns (higher exposure to tobacco and alcohol among males), health awareness (earlier help-seeking and better oral hygiene in females), and biological factors (protective effects of hormones or differential immune regulation)(Ghanem et al., 2024; Yang et al., 2023). Furthermore, QCI showed an upward trend across all regions from 1990 to 2021, with the most significant increase in middle-SDI regions, reflecting overall global improvement in oral cancer care quality and the gradual effectiveness of control measures(Ghamari et al., 2022).

The correlations between QCI and its component indicators (MIR, PIR, YLR, DPR) across SDI regions also offer policy insights. The strong positive correlation between MIR and DPR in low-SDI regions suggests a priority should be expanding access to essential healthcare services(Mohammadi et al., 2021). The positive correlation between QCI and PIR in high-SDI regions validates the successful model of improving survival through high-quality, standardized treatment(Yang et al., 2025a, Yang et al., 2025b). In low-middle SDI regions, the negative correlation between PIR and poor outcome indicators serves as a warning that merely expanding service coverage without enhancing treatment quality may fail to effectively reduce mortality rates(Lin et al., 2025). To address this, integrating palliative and supportive care services represents a critical dimension of quality enhancement that is often overlooked. Improving symptom control, pain management, and psychosocial support for advanced-stage patients through training for nurses and community health workers is not merely a compassionate add-on, but a fundamental component of effective care that can reduce suffering and potentially mitigate poor outcomes even when curative options are scarce(Huynh and Moore, 2021). This approach ensures that expanded service coverage is matched with substantive improvements in the patient experience and clinical management at all stages of disease.

This study has several limitations. First, incomplete disease registration systems in some low- and middle-low SDI countries may lead to underreporting and underestimation of the true burden. Second, reliance on GBD modeled estimates may introduce uncertainty. Third, the macro-level data cannot infer individual-level exposure-outcome relationships. Fourth, the retrospective design limits causal inference between care quality and health outcomes and may not fully capture recent advances in diagnosis and treatment.

## 5 Conclusion

From 1990 to 2021, the overall quality of care for lip and oral cavity cancer improved globally, yet significant inequities persist across regions, ages, and genders. Future strategies for LOCC control must move beyond merely tracking incidence and mortality and focus on systematically enhancing the performance of entire health systems. The QCI, as a comprehensive assessment tool, can provide crucial evidence for policymakers. We call for expanded access to primary care and standardized treatment in resource-limited settings, while in high-resource regions, attention should shift toward equitable diagnosis and management for elderly patients.

## CRediT authorship contribution statement

**Yilin Zhang:** Writing – original draft, Visualization, Software, Methodology. **Wenkai Xu:** Writing – original draft, Validation, Software, Resources. **Haocheng Wang:** Visualization, Validation, Software. **Liang Mao:** Writing – review & editing, Methodology, Data curation. **Jing Sun:** Writing – review & editing, Project administration, Funding acquisition.

## Ethical approval

Not Applicable.

## Funding

This research did not receive any specific grant from funding agencies in the public, commercial, or not-for-profit sectors.

## Declaration of competing interest

The authors declare that they have no known competing financial interests or personal relationships that could have appeared to influence the work reported in this paper.

## Data Availability

Data will be made available on request.

## Appendix A Supplementary data

Supplementary material Image 1

## References

[bb0005] GBD 2021 Diseases and Injuries Collaborators (2024). Global incidence, prevalence, years lived with disability (YLDs), disability-adjusted life-years (DALYs), and healthy life expectancy (HALE) for 371 diseases and injuries in 204 countries and territories and 811 subnational locations, 1990-2021: a systematic analysis for the global burden of disease study 2021. Lancet.

[bb0010] GBD 2019 Healthcare Access and Quality Collaborators (2022). Assessing performance of the healthcare access and quality index, overall and by select age groups, for 204 countries and territories, 1990-2019: a systematic analysis from the global burden of disease study 2019. Lancet Glob. Health.

[bb0015] Alves-Costa S., Romandini M., Nascimento G.G. (2025). Lip and Oral Cancer, caries and other Oral conditions: estimates from the 2021 global burden of disease study and projections up to 2050. J. Periodontal Res..

[bb0020] Azadnajafabad S., Saeedi Moghaddam S., Keykhaei M. (2023). Expansion of the quality of care index on breast cancer and its risk factors using the global burden of disease study 2019. Cancer Med..

[bb0025] Benzian H., Watt R., Makino Y., Stauf N., Varenne B. (2022). WHO calls to end the global crisis of oral health. Lancet.

[bb0030] Bi Y., Huang K., Wang M., Jin Y., Zheng Z.J. (2025). Global, regional and national burden and quality of care index (QCI) of leukaemia and brain and central nervous system tumours in children and adolescents aged 0-19 years: a systematic analysis of the global burden of disease study 1990-2019. BMJ Open.

[bb0035] Chen S., Yang X., Huang L., Xie Y., Li Y., Lin Y. (2025). Increasing incidence, prevalence, and mortality of lip and oral cavity cancer in adults aged 70 and older globally: findings from GBD 2021. World J. Surg. Oncol..

[bb0040] Choi E., Lee S., Nhung B.C. (2017). Cancer mortality-to-incidence ratio as an indicator of cancer management outcomes in Organization for Economic Cooperation and Development countries. Epidemiol. Health.

[bb0045] Crosbie E., Sosa P., Glantz S.A. (2016). Costa Rica’s implementation of the framework convention on tobacco control: overcoming decades of industry dominance. Salud Publica Mex..

[bb0050] Dai R., Zhang Y., Zou H., Li H., Zhang S. (2025). Increased burden of lip, oral, and pharyngeal cancer in adolescents and young adults from 1990 to 2021. BMC Oral Health.

[bb0055] Dresang L.T., Brebrick L., Murray D., Shallue A., Sullivan-Vedder L. (2005). Family medicine in Cuba: community-oriented primary care and complementary and alternative medicine. J. Am. Board Fam. Pract..

[bb0060] Fan K.M., Rimal J., Zhang P., Johnson N.W. (2022). Stark differences in cancer epidemiological data between GLOBOCAN and GBD: emphasis on oral cancer and wider implications. EClinicalMedicine.

[bb0065] GBD (2023).

[bb0070] Bernabe E., GBD 2017 Oral Disorders Collaborators (2020). Global, regional, and National Levels and trends in burden of Oral conditions from 1990 to 2017: a systematic analysis for the global burden of disease 2017 study. J. Dent. Res..

[bb0075] Ghafouri M., Ghasemi E., Rostami M. (2023). The quality of care index for low back pain: a systematic analysis of the global burden of disease study 1990-2017. Archives Publi. Health Archives Belges de Sante Publi..

[bb0080] Ghamari S.H., Yoosefi M., Abbasi-Kangevari M. (2022). Trends in global, regional, and National Burden and quality of care index for liver Cancer by cause from global burden of disease 1990-2019. Hepatol. Commun..

[bb0085] Ghanem A.S., Memon H.A., Nagy A.C. (2024). Evolving trends in oral cancer burden in Europe: a systematic review. Front. Oncol..

[bb0090] Huynh L., Moore J. (2021). Palliative and end-of-life care for the older adult with cancer. Curr. Opin. Support. Palliat. Care.

[bb0095] Lin L., Xu J., Chai Y., Wu W. (2025). Global, regional, and national burden of infective endocarditis from 2010 to 2021 and predictions for the next five years: results from the global burden of disease study 2021. BMC Public Health.

[bb0100] Liu Y., Han B. (2025). Global, regional, and national burden trends of lip and oral cavity cancer among individuals aged 60 and above from 1990 to 2021: a systematic analysis based on the 2021 global burden of disease data. BMC Cancer.

[bb0105] Luo Z., Jiang D., Shan S. (2025). Cross-country inequalities in disease burden and quality of Care of Stroke, 1990-2021: a systematic analysis of the global burden of disease study 2021. Eur. J. Neurol..

[bb0110] Meng S., Lv A., Li N., Ding X. (2025). Global, regional, and national burden of lip and oral cavity cancer and projections to 2036. BMC Cancer.

[bb0115] Mohammadi E., Ghasemi E., Azadnajafabad S. (2021). A global, regional, and national survey on burden and quality of care index (QCI) of brain and other central nervous system cancers; global burden of disease systematic analysis 1990-2017. PLoS One.

[bb0120] Pan Y., Liu Q., Zhang N., Peng S., Li X., Zhou F. (2025). Global assessment of leukemia care quality: insights from the quality of care index (QCI) from 1990 to 2021. EClinicalMedicine.

[bb0125] Ringnér M. (2008). What is principal component analysis. Nat. Biotechnol..

[bb0130] Sofi-Mahmudi A., Masinaei M., Shamsoddin E. (2021). Global, regional, and national burden and quality of care index (QCI) of lip and oral cavity cancer: a systematic analysis of the global burden of disease study 1990-2017. BMC Oral Health.

[bb0135] Wu J., Chen H., Liu Y., Yang R., An N. (2025). The global, regional, and national burden of oral cancer, 1990-2021: a systematic analysis for the global burden of disease study 2021. J. Cancer Res. Clin. Oncol..

[bb0140] Wu S., Zhang Z., Yuan W., Yang J., Huang X. (2025). Analysis of lip and oral cavity cancer burden between China and the global from 1990 to 2021 and projections for the next fifteen years. BMC Oral Health.

[bb0145] Xu C., Zhou E., Shen Y. (2025). Assessment and future projection of brain and central nervous system cancer burden using a modified quality care index: evidence from the global burden of disease 2021. Eur. J. Oncol. Nurs..

[bb0150] Yang Y.P., Hsin H.T., Wang B.L. (2023). Gender differences in oral health among prisoners: a cross-sectional study from Taiwan. BMC Oral Health.

[bb0155] Yang M., Wang G., Lou Y., Xuan F. (2025). Trends and inequalities in gastrointestinal cancer care quality from 1990 to 2021: a population-based analysis of the global burden of disease study 2021. Medicine.

[bb0160] Yang J., Zhu L., Tang W. (2025). Global, regional, and national burden and quality of care index of five head and neck cancers: a 32-year longitudinal analysis. Eur. Arch. Otorrinolaringol..

[bb0165] Yu Z., Ma X., Xiao H. (2024). Disease burden and attributable risk factors of lip and oral cavity cancer in China from 1990 to 2021 and its prediction to 2031. Front. Public Health.

