# Supplementary material for: Global quality of care trends for lip and oral cavity cancer: A quality of care index analysis, 1990–2021
Source: Prev Med Rep. 2026 Jan 7;62:103380. doi: 10.1016/j.pmedr.2026.103380 (PMC12861060; doi:10.1016/j.pmedr.2026.103380)
Supplement: Supplementary file 1 — Supplementary material [file mmc1.pdf]

Supplementary Materials

Table S1. Incidence and mortality cases and rates for lip and oral cavity cancer among individuals aged 15 years and older, by Socio-demographic Index region, age group, and year (1990 and 2021). 1

Table S2. Country-level ranking and stratum of the Quality of Care Index for lip and oral cavity cancer among individuals aged 15 years and older, 2021. 17

Table S3. Age- and sex-specific Quality of Care Index and related component indicators for lip and oral cavity cancer among individuals aged 15 years and older, by global region and Socio-demographic Index, 2021. 22

Table S4. Univariate analysis of the Quality of Care Index and related indicators for lip and oral cavity cancer among individuals aged 15 years and older, 1990–2021: values stratified by sex and Socio-demographic Index region. 36

Table S1. Incidence and mortality cases and rates for lip and oral cavity cancer among individuals aged 15 years and older, by Socio-demographic Index region, age group, and year (1990 and 2021).

	group	measure_name	location_name	sex_name	age_name	cause_name	metric_name	year	val	upper	lower	color
1	High-middle SDI/1990	Incidence	High-middle SDI	Both	95+ years	Lip and oral cavity cancer	Number	1990	37.3	41.3	29.8	#C84150
2	High-middle SDI/1990	Incidence	High-middle SDI	Both	30-34 years	Lip and oral cavity cancer	Number	1990	532.8	572.8	499.0	#C84150
3	High-middle SDI/1990	Incidence	High-middle SDI	Both	15-19 years	Lip and oral cavity cancer	Number	1990	100.5	110.7	92.1	#C84150
4	High-middle SDI/1990	Incidence	High-middle SDI	Both	50-54 years	Lip and oral cavity cancer	Number	1990	4882.8	5139.1	4663.6	#C84150
5	High-middle SDI/1990	Incidence	High-middle SDI	Both	20-24 years	Lip and oral cavity cancer	Number	1990	149.2	163.7	137.2	#C84150
6	High-middle SDI/1990	Incidence	High-middle SDI	Both	55-59 years	Lip and oral cavity cancer	Number	1990	5498.2	5803.6	5197.0	#C84150
7	High-middle SDI/1990	Incidence	High-middle SDI	Both	25-29 years	Lip and oral cavity cancer	Number	1990	266.2	287.7	248.8	#C84150
8	High-middle SDI/1990	Incidence	High-middle SDI	Both	60-64 years	Lip and oral cavity cancer	Number	1990	5943.9	6265.4	5649.8	#C84150
9	High-middle SDI/1990	Incidence	High-middle SDI	Both	40-44 years	Lip and oral cavity cancer	Number	1990	1908.3	2005.5	1819.2	#C84150
10	High-middle SDI/1990	Incidence	High-middle SDI	Both	65-69 years	Lip and oral cavity cancer	Number	1990	4697.3	4978.7	4422.0	#C84150
11	High-middle SDI/1990	Incidence	High-middle SDI	Both	35-39 years	Lip and oral cavity cancer	Number	1990	1204.4	1270.1	1140.1	#C84150
12	High-middle SDI/1990	Incidence	High-middle SDI	Both	70-74 years	Lip and oral cavity cancer	Number	1990	3196.2	3403.0	3000.4	#C84150
13	High-middle SDI/1990	Incidence	High-middle SDI	Both	75-79 years	Lip and oral cavity cancer	Number	1990	2843.6	2987.9	2670.3	#C84150
14	High-middle SDI/1990	Incidence	High-middle SDI	Both	45-49 years	Lip and oral cavity cancer	Number	1990	2691.2	2848.2	2566.5	#C84150
15	High-middle SDI/1990	Incidence	High-middle SDI	Both	80-84 years	Lip and oral cavity cancer	Number	1990	1757.5	1875.8	1605.6	#C84150
16	High-middle SDI/1990	Incidence	High-middle SDI	Both	85-89 years	Lip and oral cavity cancer	Number	1990	767.2	828.1	675.1	#C84150
17	High-middle SDI/1990	Incidence	High-middle SDI	Both	90-94 years	Lip and oral cavity cancer	Number	1990	208.9	228.4	178.0	#C84150
18	High-middle SDI/2021	Incidence	High-middle SDI	Both	95+ years	Lip and oral cavity cancer	Number	2021	255.9	291.2	190.9	#4484B0
19	High-middle SDI/2021	Incidence	High-middle SDI	Both	15-19 years	Lip and oral cavity cancer	Number	2021	84.2	94.1	73.7	#4484B0
20	High-middle SDI/2021	Incidence	High-middle SDI	Both	20-24 years	Lip and oral cavity cancer	Number	2021	148.7	166.8	131.8	#4484B0

21	High-middle SDI/2021	Incidence	High-middle SDI	Both	25-29 years	Lip and oral cavity cancer	Number	2021	322.9	361.8	283.1	#4484B0
22	High-middle SDI/2021	Incidence	High-middle SDI	Both	60-64 years	Lip and oral cavity cancer	Number	2021	10807.5	11765.8	9817.6	#4484B0
23	High-middle SDI/2021	Incidence	High-middle SDI	Both	30-34 years	Lip and oral cavity cancer	Number	2021	933.6	1043.0	836.7	#4484B0
24	High-middle SDI/2021	Incidence	High-middle SDI	Both	65-69 years	Lip and oral cavity cancer	Number	2021	11022.1	12232.9	9886.3	#4484B0
25	High-middle SDI/2021	Incidence	High-middle SDI	Both	35-39 years	Lip and oral cavity cancer	Number	2021	1959.0	2128.6	1788.4	#4484B0
26	High-middle SDI/2021	Incidence	High-middle SDI	Both	70-74 years	Lip and oral cavity cancer	Number	2021	9157.6	10178.6	8208.7	#4484B0
27	High-middle SDI/2021	Incidence	High-middle SDI	Both	40-44 years	Lip and oral cavity cancer	Number	2021	2912.1	3165.3	2679.3	#4484B0
28	High-middle SDI/2021	Incidence	High-middle SDI	Both	75-79 years	Lip and oral cavity cancer	Number	2021	6102.5	6816.6	5393.9	#4484B0
29	High-middle SDI/2021	Incidence	High-middle SDI	Both	45-49 years	Lip and oral cavity cancer	Number	2021	4866.3	5330.7	4466.2	#4484B0
30	High-middle SDI/2021	Incidence	High-middle SDI	Both	80-84 years	Lip and oral cavity cancer	Number	2021	4798.5	5272.2	4161.7	#4484B0
31	High-middle SDI/2021	Incidence	High-middle SDI	Both	50-54 years	Lip and oral cavity cancer	Number	2021	7522.8	8307.5	6812.0	#4484B0
32	High-middle SDI/2021	Incidence	High-middle SDI	Both	85-89 years	Lip and oral cavity cancer	Number	2021	3067.8	3416.6	2556.7	#4484B0
33	High-middle SDI/2021	Incidence	High-middle SDI	Both	55-59 years	Lip and oral cavity cancer	Number	2021	10228.4	11250.9	9331.2	#4484B0
34	High-middle SDI/2021	Incidence	High-middle SDI	Both	90-94 years	Lip and oral cavity cancer	Number	2021	1192.1	1329.9	953.0	#4484B0
35	High SDI/1990	Incidence	High SDI	Both	95+ years	Lip and oral cavity cancer	Number	1990	140.5	158.9	104.9	#8F243D
36	High SDI/1990	Incidence	High SDI	Both	45-49 years	Lip and oral cavity cancer	Number	1990	4067.7	4327.2	3847.9	#8F243D
37	High SDI/1990	Incidence	High SDI	Both	15-19 years	Lip and oral cavity cancer	Number	1990	95.7	99.7	92.1	#8F243D
38	High SDI/1990	Incidence	High SDI	Both	50-54 years	Lip and oral cavity cancer	Number	1990	5599.4	5977.1	5288.0	#8F243D
39	High SDI/1990	Incidence	High SDI	Both	20-24 years	Lip and oral cavity cancer	Number	1990	178.6	186.4	171.1	#8F243D
40	High SDI/1990	Incidence	High SDI	Both	55-59 years	Lip and oral cavity cancer	Number	1990	7183.0	7603.8	6792.6	#8F243D
41	High SDI/1990	Incidence	High SDI	Both	25-29 years	Lip and oral cavity cancer	Number	1990	381.7	397.1	366.9	#8F243D
42	High SDI/1990	Incidence	High SDI	Both	60-64 years	Lip and oral cavity cancer	Number	1990	8224.5	8662.4	7860.5	#8F243D

43	High SDI/1990	Incidence	High SDI	Both	30-34 years	Lip and oral cavity cancer	Number	1990	759.8	790.6	728.3	#8F243D
44	High SDI/1990	Incidence	High SDI	Both	65-69 years	Lip and oral cavity cancer	Number	1990	8217.2	8574.0	7854.5	#8F243D
45	High SDI/1990	Incidence	High SDI	Both	35-39 years	Lip and oral cavity cancer	Number	1990	1723.5	1812.9	1638.3	#8F243D
46	High SDI/1990	Incidence	High SDI	Both	70-74 years	Lip and oral cavity cancer	Number	1990	6427.0	6688.7	6118.1	#8F243D
47	High SDI/1990	Incidence	High SDI	Both	40-44 years	Lip and oral cavity cancer	Number	1990	2716.2	2892.0	2587.7	#8F243D
48	High SDI/1990	Incidence	High SDI	Both	75-79 years	Lip and oral cavity cancer	Number	1990	5846.2	6105.6	5487.2	#8F243D
49	High SDI/1990	Incidence	High SDI	Both	80-84 years	Lip and oral cavity cancer	Number	1990	3852.9	4087.0	3444.0	#8F243D
50	High SDI/1990	Incidence	High SDI	Both	85-89 years	Lip and oral cavity cancer	Number	1990	2090.3	2254.4	1772.8	#8F243D
51	High SDI/1990	Incidence	High SDI	Both	90-94 years	Lip and oral cavity cancer	Number	1990	699.3	770.0	562.2	#8F243D
52	High SDI/2021	Incidence	High SDI	Both	95+ years	Lip and oral cavity cancer	Number	2021	1061.9	1242.1	739.8	#6752A9
53	High SDI/2021	Incidence	High SDI	Both	15-19 years	Lip and oral cavity cancer	Number	2021	81.1	85.9	75.5	#6752A9
54	High SDI/2021	Incidence	High SDI	Both	20-24 years	Lip and oral cavity cancer	Number	2021	206.6	217.3	196.1	#6752A9
55	High SDI/2021	Incidence	High SDI	Both	25-29 years	Lip and oral cavity cancer	Number	2021	467.1	494.9	441.3	#6752A9
56	High SDI/2021	Incidence	High SDI	Both	60-64 years	Lip and oral cavity cancer	Number	2021	13572.7	14306.4	12919.4	#6752A9
57	High SDI/2021	Incidence	High SDI	Both	30-34 years	Lip and oral cavity cancer	Number	2021	945.2	997.3	900.8	#6752A9
58	High SDI/2021	Incidence	High SDI	Both	65-69 years	Lip and oral cavity cancer	Number	2021	14547.9	15237.2	13741.5	#6752A9
59	High SDI/2021	Incidence	High SDI	Both	35-39 years	Lip and oral cavity cancer	Number	2021	1965.6	2072.2	1866.8	#6752A9
60	High SDI/2021	Incidence	High SDI	Both	70-74 years	Lip and oral cavity cancer	Number	2021	14561.6	15318.3	13581.2	#6752A9
61	High SDI/2021	Incidence	High SDI	Both	40-44 years	Lip and oral cavity cancer	Number	2021	2788.4	2985.7	2618.2	#6752A9
62	High SDI/2021	Incidence	High SDI	Both	75-79 years	Lip and oral cavity cancer	Number	2021	11398.7	12201.1	10274.5	#6752A9
63	High SDI/2021	Incidence	High SDI	Both	45-49 years	Lip and oral cavity cancer	Number	2021	4514.9	4796.4	4267.4	#6752A9
64	High SDI/2021	Incidence	High SDI	Both	80-84 years	Lip and oral cavity cancer	Number	2021	9244.6	10154.5	7725.3	#6752A9

65	High SDI/2021	Incidence	High SDI	Both	50-54 years	Lip and oral cavity cancer	Number	2021	7422.8	7836.0	7020.0	#6752A9
66	High SDI/2021	Incidence	High SDI	Both	85-89 years	Lip and oral cavity cancer	Number	2021	7041.0	7915.6	5439.0	#6752A9
67	High SDI/2021	Incidence	High SDI	Both	55-59 years	Lip and oral cavity cancer	Number	2021	11351.5	12014.4	10723.1	#6752A9
68	High SDI/2021	Incidence	High SDI	Both	90-94 years	Lip and oral cavity cancer	Number	2021	3699.6	4244.0	2790.8	#6752A9
69	Low-middle SDI/1990	Incidence	Low-middle SDI	Both	15-19 years	Lip and oral cavity cancer	Number	1990	265.1	309.3	224.9	#FDAD6B
70	Low-middle SDI/1990	Incidence	Low-middle SDI	Both	50-54 years	Lip and oral cavity cancer	Number	1990	4253.5	4919.2	3733.1	#FDAD6B
71	Low-middle SDI/1990	Incidence	Low-middle SDI	Both	20-24 years	Lip and oral cavity cancer	Number	1990	413.5	472.7	359.0	#FDAD6B
72	Low-middle SDI/1990	Incidence	Low-middle SDI	Both	55-59 years	Lip and oral cavity cancer	Number	1990	4729.1	5403.1	4205.6	#FDAD6B
73	Low-middle SDI/1990	Incidence	Low-middle SDI	Both	25-29 years	Lip and oral cavity cancer	Number	1990	626.6	728.0	536.1	#FDAD6B
74	Low-middle SDI/1990	Incidence	Low-middle SDI	Both	65-69 years	Lip and oral cavity cancer	Number	1990	4557.6	5198.1	3927.3	#FDAD6B
75	Low-middle SDI/1990	Incidence	Low-middle SDI	Both	30-34 years	Lip and oral cavity cancer	Number	1990	992.2	1149.5	851.1	#FDAD6B
76	Low-middle SDI/1990	Incidence	Low-middle SDI	Both	60-64 years	Lip and oral cavity cancer	Number	1990	5259.8	6001.1	4612.9	#FDAD6B
77	Low-middle SDI/1990	Incidence	Low-middle SDI	Both	35-39 years	Lip and oral cavity cancer	Number	1990	1689.4	1940.2	1436.6	#FDAD6B
78	Low-middle SDI/1990	Incidence	Low-middle SDI	Both	75-79 years	Lip and oral cavity cancer	Number	1990	2170.9	2461.3	1836.5	#FDAD6B
79	Low-middle SDI/1990	Incidence	Low-middle SDI	Both	40-44 years	Lip and oral cavity cancer	Number	1990	2397.1	2717.2	2080.7	#FDAD6B
80	Low-middle SDI/1990	Incidence	Low-middle SDI	Both	70-74 years	Lip and oral cavity cancer	Number	1990	3275.9	3739.4	2855.1	#FDAD6B
81	Low-middle SDI/1990	Incidence	Low-middle SDI	Both	45-49 years	Lip and oral cavity cancer	Number	1990	3111.9	3540.8	2766.4	#FDAD6B
82	Low-middle SDI/1990	Incidence	Low-middle SDI	Both	95+ years	Lip and oral cavity cancer	Number	1990	27.9	34.0	22.1	#FDAD6B
83	Low-middle SDI/1990	Incidence	Low-middle SDI	Both	85-89 years	Lip and oral cavity cancer	Number	1990	481.4	563.2	400.5	#FDAD6B
84	Low-middle SDI/1990	Incidence	Low-middle SDI	Both	80-84 years	Lip and oral cavity cancer	Number	1990	1129.6	1306.6	947.5	#FDAD6B
85	Low-middle SDI/1990	Incidence	Low-middle SDI	Both	90-94 years	Lip and oral cavity cancer	Number	1990	139.2	163.8	116.5	#FDAD6B
86	Low-middle SDI/2021	Incidence	Low-middle SDI	Both	20-24 years	Lip and oral cavity cancer	Number	2021	1059.0	1424.9	738.7	#BCF0AC

87	Low-middle SDI/2021	Incidence	Low-middle SDI	Both	25-29 years	Lip and oral cavity cancer	Number	2021	1660. 9	2119. 1	1222.1	#BCF0AC
88	Low-middle SDI/2021	Incidence	Low-middle SDI	Both	15-19 years	Lip and oral cavity cancer	Number	2021	589.5	783.8	414.3	#BCF0AC
89	Low-middle SDI/2021	Incidence	Low-middle SDI	Both	30-34 years	Lip and oral cavity cancer	Number	2021	2839. 5	3480. 8	2165.4	#BCF0AC
90	Low-middle SDI/2021	Incidence	Low-middle SDI	Both	60-64 years	Lip and oral cavity cancer	Number	2021	13632 .5	15667 .3	11636.4	#BCF0AC
91	Low-middle SDI/2021	Incidence	Low-middle SDI	Both	95+ years	Lip and oral cavity cancer	Number	2021	160.6	189.3	125.3	#BCF0AC
92	Low-middle SDI/2021	Incidence	Low-middle SDI	Both	65-69 years	Lip and oral cavity cancer	Number	2021	13005 .2	14619 .3	11362.6	#BCF0AC
93	Low-middle SDI/2021	Incidence	Low-middle SDI	Both	40-44 years	Lip and oral cavity cancer	Number	2021	6804. 8	7828. 6	5748.5	#BCF0AC
94	Low-middle SDI/2021	Incidence	Low-middle SDI	Both	70-74 years	Lip and oral cavity cancer	Number	2021	10685 .5	11993 .5	9259.1	#BCF0AC
95	Low-middle SDI/2021	Incidence	Low-middle SDI	Both	35-39 years	Lip and oral cavity cancer	Number	2021	4717. 8	5634. 4	3726.5	#BCF0AC
96	Low-middle SDI/2021	Incidence	Low-middle SDI	Both	75-79 years	Lip and oral cavity cancer	Number	2021	7242. 9	8133. 4	6388.9	#BCF0AC
97	Low-middle SDI/2021	Incidence	Low-middle SDI	Both	50-54 years	Lip and oral cavity cancer	Number	2021	11281 .9	12907 .0	9740.6	#BCF0AC
98	Low-middle SDI/2021	Incidence	Low-middle SDI	Both	80-84 years	Lip and oral cavity cancer	Number	2021	4243. 8	4787. 7	3687.5	#BCF0AC
99	Low-middle SDI/2021	Incidence	Low-middle SDI	Both	45-49 years	Lip and oral cavity cancer	Number	2021	8351. 2	9676. 1	7006.7	#BCF0AC
100	Low-middle SDI/2021	Incidence	Low-middle SDI	Both	85-89 years	Lip and oral cavity cancer	Number	2021	2056. 7	2351. 7	1742.9	#BCF0AC
101	Low-middle SDI/2021	Incidence	Low-middle SDI	Both	55-59 years	Lip and oral cavity cancer	Number	2021	12962 .9	14804 .2	11201.0	#BCF0AC
102	Low-middle SDI/2021	Incidence	Low-middle SDI	Both	90-94 years	Lip and oral cavity cancer	Number	2021	745.1	853.4	618.4	#BCF0AC
103	Low SDI/1990	Incidence	Low SDI	Both	95+ years	Lip and oral cavity cancer	Number	1990	4.9	6.3	3.8	#FADA93
104	Low SDI/1990	Incidence	Low SDI	Both	45-49 years	Lip and oral cavity cancer	Number	1990	795.7	930.2	666.3	#FADA93
105	Low SDI/1990	Incidence	Low SDI	Both	15-19 years	Lip and oral cavity cancer	Number	1990	80.6	99.8	64.5	#FADA93
106	Low SDI/1990	Incidence	Low SDI	Both	50-54 years	Lip and oral cavity cancer	Number	1990	1147. 0	1365. 4	970.6	#FADA93
107	Low SDI/1990	Incidence	Low SDI	Both	20-24 years	Lip and oral cavity cancer	Number	1990	108.8	133.3	86.3	#FADA93
108	Low SDI/1990	Incidence	Low SDI	Both	55-59 years	Lip and oral cavity cancer	Number	1990	1346. 8	1556. 8	1143.9	#FADA93

109	Low SDI/1990	Incidence	Low SDI	Both	25-29 years	Lip and oral cavity cancer	Number	1990	159.9	195.1	125.0	#FADA93
110	Low SDI/1990	Incidence	Low SDI	Both	60-64 years	Lip and oral cavity cancer	Number	1990	1434.1	1653.4	1198.6	#FADA93
111	Low SDI/1990	Incidence	Low SDI	Both	30-34 years	Lip and oral cavity cancer	Number	1990	249.2	299.5	200.1	#FADA93
112	Low SDI/1990	Incidence	Low SDI	Both	65-69 years	Lip and oral cavity cancer	Number	1990	1249.2	1443.4	1044.8	#FADA93
113	Low SDI/1990	Incidence	Low SDI	Both	35-39 years	Lip and oral cavity cancer	Number	1990	435.6	522.2	347.3	#FADA93
114	Low SDI/1990	Incidence	Low SDI	Both	70-74 years	Lip and oral cavity cancer	Number	1990	915.2	1059.4	768.4	#FADA93
115	Low SDI/1990	Incidence	Low SDI	Both	40-44 years	Lip and oral cavity cancer	Number	1990	594.7	688.4	494.5	#FADA93
116	Low SDI/1990	Incidence	Low SDI	Both	75-79 years	Lip and oral cavity cancer	Number	1990	568.8	663.6	483.8	#FADA93
117	Low SDI/1990	Incidence	Low SDI	Both	80-84 years	Lip and oral cavity cancer	Number	1990	287.1	334.4	244.0	#FADA93
118	Low SDI/1990	Incidence	Low SDI	Both	85-89 years	Lip and oral cavity cancer	Number	1990	99.3	115.9	83.2	#FADA93
119	Low SDI/1990	Incidence	Low SDI	Both	90-94 years	Lip and oral cavity cancer	Number	1990	24.9	29.5	20.5	#FADA93
120	Low SDI/2021	Incidence	Low SDI	Both	95+ years	Lip and oral cavity cancer	Number	2021	20.5	25.0	16.2	#EFF5A4
121	Low SDI/2021	Incidence	Low SDI	Both	15-19 years	Lip and oral cavity cancer	Number	2021	238.5	319.1	176.2	#EFF5A4
122	Low SDI/2021	Incidence	Low SDI	Both	20-24 years	Lip and oral cavity cancer	Number	2021	347.4	479.3	249.1	#EFF5A4
123	Low SDI/2021	Incidence	Low SDI	Both	25-29 years	Lip and oral cavity cancer	Number	2021	485.9	618.2	365.6	#EFF5A4
124	Low SDI/2021	Incidence	Low SDI	Both	60-64 years	Lip and oral cavity cancer	Number	2021	3305.3	3903.3	2767.8	#EFF5A4
125	Low SDI/2021	Incidence	Low SDI	Both	30-34 years	Lip and oral cavity cancer	Number	2021	797.3	1045.3	593.6	#EFF5A4
126	Low SDI/2021	Incidence	Low SDI	Both	65-69 years	Lip and oral cavity cancer	Number	2021	3044.7	3600.2	2562.2	#EFF5A4
127	Low SDI/2021	Incidence	Low SDI	Both	35-39 years	Lip and oral cavity cancer	Number	2021	1295.6	1606.0	1014.6	#EFF5A4
128	Low SDI/2021	Incidence	Low SDI	Both	70-74 years	Lip and oral cavity cancer	Number	2021	2553.8	3041.2	2110.2	#EFF5A4
129	Low SDI/2021	Incidence	Low SDI	Both	40-44 years	Lip and oral cavity cancer	Number	2021	1697.0	2066.7	1367.0	#EFF5A4
130	Low SDI/2021	Incidence	Low SDI	Both	75-79 years	Lip and oral cavity cancer	Number	2021	1671.3	1934.4	1399.5	#EFF5A4

131	Low SDI/2021	Incidence	Low SDI	Both	45-49 years	Lip and oral cavity cancer	Number	2021	2145.8	2577.3	1709.3	#EFF5A4
132	Low SDI/2021	Incidence	Low SDI	Both	80-84 years	Lip and oral cavity cancer	Number	2021	1011.5	1163.1	857.7	#EFF5A4
133	Low SDI/2021	Incidence	Low SDI	Both	50-54 years	Lip and oral cavity cancer	Number	2021	2776.1	3348.5	2243.3	#EFF5A4
134	Low SDI/2021	Incidence	Low SDI	Both	85-89 years	Lip and oral cavity cancer	Number	2021	421.4	499.7	349.9	#EFF5A4
135	Low SDI/2021	Incidence	Low SDI	Both	55-59 years	Lip and oral cavity cancer	Number	2021	3160.9	3651.9	2676.7	#EFF5A4
136	Low SDI/2021	Incidence	Low SDI	Both	90-94 years	Lip and oral cavity cancer	Number	2021	116.5	137.9	95.9	#EFF5A4
137	Middle SDI/1990	Incidence	Middle SDI	Both	95+ years	Lip and oral cavity cancer	Number	1990	33.5	40.1	26.6	#F06D49
138	Middle SDI/1990	Incidence	Middle SDI	Both	40-44 years	Lip and oral cavity cancer	Number	1990	2103.1	2275.4	1946.0	#F06D49
139	Middle SDI/1990	Incidence	Middle SDI	Both	75-79 years	Lip and oral cavity cancer	Number	1990	2312.0	2490.3	2131.1	#F06D49
140	Middle SDI/1990	Incidence	Middle SDI	Both	45-49 years	Lip and oral cavity cancer	Number	1990	2848.5	3107.5	2651.0	#F06D49
141	Middle SDI/1990	Incidence	Middle SDI	Both	15-19 years	Lip and oral cavity cancer	Number	1990	222.8	245.7	203.7	#F06D49
142	Middle SDI/1990	Incidence	Middle SDI	Both	50-54 years	Lip and oral cavity cancer	Number	1990	3997.2	4350.9	3673.1	#F06D49
143	Middle SDI/1990	Incidence	Middle SDI	Both	20-24 years	Lip and oral cavity cancer	Number	1990	339.2	373.9	309.9	#F06D49
144	Middle SDI/1990	Incidence	Middle SDI	Both	55-59 years	Lip and oral cavity cancer	Number	1990	4716.0	5129.7	4375.6	#F06D49
145	Middle SDI/1990	Incidence	Middle SDI	Both	25-29 years	Lip and oral cavity cancer	Number	1990	546.3	600.2	502.8	#F06D49
146	Middle SDI/1990	Incidence	Middle SDI	Both	60-64 years	Lip and oral cavity cancer	Number	1990	4744.5	5144.7	4371.9	#F06D49
147	Middle SDI/1990	Incidence	Middle SDI	Both	30-34 years	Lip and oral cavity cancer	Number	1990	927.4	1011.4	857.6	#F06D49
148	Middle SDI/1990	Incidence	Middle SDI	Both	65-69 years	Lip and oral cavity cancer	Number	1990	4174.2	4519.1	3852.0	#F06D49
149	Middle SDI/1990	Incidence	Middle SDI	Both	35-39 years	Lip and oral cavity cancer	Number	1990	1637.0	1776.5	1501.0	#F06D49
150	Middle SDI/1990	Incidence	Middle SDI	Both	70-74 years	Lip and oral cavity cancer	Number	1990	3265.5	3506.4	3007.3	#F06D49
151	Middle SDI/1990	Incidence	Middle SDI	Both	85-89 years	Lip and oral cavity cancer	Number	1990	627.0	683.9	561.7	#F06D49
152	Middle SDI/1990	Incidence	Middle SDI	Both	80-84 years	Lip and oral cavity cancer	Number	1990	1315.0	1415.7	1202.9	#F06D49

153	Middle SDI/1990	Incidence	Middle SDI	Both	90-94 years	Lip and oral cavity cancer	Number	1990	172.0	192.1	147.8	#F06D49
154	Middle SDI/2021	Incidence	Middle SDI	Both	95+ years	Lip and oral cavity cancer	Number	2021	261.4	306.8	196.2	#7ED4B6
155	Middle SDI/2021	Incidence	Middle SDI	Both	75-79 years	Lip and oral cavity cancer	Number	2021	9357.0	10423.7	8336.9	#7ED4B6
156	Middle SDI/2021	Incidence	Middle SDI	Both	15-19 years	Lip and oral cavity cancer	Number	2021	283.4	329.5	227.2	#7ED4B6
157	Middle SDI/2021	Incidence	Middle SDI	Both	40-44 years	Lip and oral cavity cancer	Number	2021	5774.0	6542.4	5026.2	#7ED4B6
158	Middle SDI/2021	Incidence	Middle SDI	Both	20-24 years	Lip and oral cavity cancer	Number	2021	519.4	600.0	437.2	#7ED4B6
159	Middle SDI/2021	Incidence	Middle SDI	Both	25-29 years	Lip and oral cavity cancer	Number	2021	1048.3	1207.8	879.2	#7ED4B6
160	Middle SDI/2021	Incidence	Middle SDI	Both	60-64 years	Lip and oral cavity cancer	Number	2021	14844.7	16412.0	13280.7	#7ED4B6
161	Middle SDI/2021	Incidence	Middle SDI	Both	30-34 years	Lip and oral cavity cancer	Number	2021	2234.5	2496.9	1960.7	#7ED4B6
162	Middle SDI/2021	Incidence	Middle SDI	Both	65-69 years	Lip and oral cavity cancer	Number	2021	15802.1	17699.6	13928.6	#7ED4B6
163	Middle SDI/2021	Incidence	Middle SDI	Both	35-39 years	Lip and oral cavity cancer	Number	2021	3980.1	4515.7	3462.5	#7ED4B6
164	Middle SDI/2021	Incidence	Middle SDI	Both	70-74 years	Lip and oral cavity cancer	Number	2021	13082.1	14799.5	11504.6	#7ED4B6
165	Middle SDI/2021	Incidence	Middle SDI	Both	50-54 years	Lip and oral cavity cancer	Number	2021	12286.5	13779.9	10778.1	#7ED4B6
166	Middle SDI/2021	Incidence	Middle SDI	Both	85-89 years	Lip and oral cavity cancer	Number	2021	3459.3	3884.2	2951.4	#7ED4B6
167	Middle SDI/2021	Incidence	Middle SDI	Both	45-49 years	Lip and oral cavity cancer	Number	2021	8541.7	9616.7	7439.6	#7ED4B6
168	Middle SDI/2021	Incidence	Middle SDI	Both	80-84 years	Lip and oral cavity cancer	Number	2021	5982.2	6628.7	5194.1	#7ED4B6
169	Middle SDI/2021	Incidence	Middle SDI	Both	55-59 years	Lip and oral cavity cancer	Number	2021	15187.7	17192.2	13538.1	#7ED4B6
170	Middle SDI/2021	Incidence	Middle SDI	Both	90-94 years	Lip and oral cavity cancer	Number	2021	1213.4	1378.6	996.1	#7ED4B6
171	High-middle SDI/1990	Deaths	High-middle SDI	Both	15-19 years	Lip and oral cavity cancer	Number	1990	37.7	41.8	34.5	#C84150
172	High-middle SDI/1990	Deaths	High-middle SDI	Both	20-24 years	Lip and oral cavity cancer	Number	1990	57.9	63.5	53.1	#C84150
173	High-middle SDI/1990	Deaths	High-middle SDI	Both	25-29 years	Lip and oral cavity cancer	Number	1990	91.0	99.1	85.1	#C84150
174	High-middle SDI/1990	Deaths	High-middle SDI	Both	30-34 years	Lip and oral cavity cancer	Number	1990	181.4	196.9	169.5	#C84150

175	High-middle SDI/1990	Deaths	High-middle SDI	Both	35-39 years	Lip and oral cavity cancer	Number	1990	386.6	410.7	365.3	#C84150
176	High-middle SDI/1990	Deaths	High-middle SDI	Both	40-44 years	Lip and oral cavity cancer	Number	1990	767.6	807.8	733.5	#C84150
177	High-middle SDI/1990	Deaths	High-middle SDI	Both	45-49 years	Lip and oral cavity cancer	Number	1990	1229.8	1296.7	1171.4	#C84150
178	High-middle SDI/1990	Deaths	High-middle SDI	Both	50-54 years	Lip and oral cavity cancer	Number	1990	2394.0	2507.9	2281.0	#C84150
179	High-middle SDI/1990	Deaths	High-middle SDI	Both	55-59 years	Lip and oral cavity cancer	Number	1990	2786.2	2934.2	2648.2	#C84150
180	High-middle SDI/1990	Deaths	High-middle SDI	Both	60-64 years	Lip and oral cavity cancer	Number	1990	3207.6	3362.0	3066.6	#C84150
181	High-middle SDI/1990	Deaths	High-middle SDI	Both	65-69 years	Lip and oral cavity cancer	Number	1990	2535.6	2663.0	2409.2	#C84150
182	High-middle SDI/1990	Deaths	High-middle SDI	Both	70-74 years	Lip and oral cavity cancer	Number	1990	1831.5	1944.5	1719.1	#C84150
183	High-middle SDI/1990	Deaths	High-middle SDI	Both	75-79 years	Lip and oral cavity cancer	Number	1990	1813.3	1897.8	1722.2	#C84150
184	High-middle SDI/1990	Deaths	High-middle SDI	Both	80-84 years	Lip and oral cavity cancer	Number	1990	1260.8	1333.5	1163.6	#C84150
185	High-middle SDI/1990	Deaths	High-middle SDI	Both	85-89 years	Lip and oral cavity cancer	Number	1990	633.4	676.2	562.0	#C84150
186	High-middle SDI/1990	Deaths	High-middle SDI	Both	90-94 years	Lip and oral cavity cancer	Number	1990	213.1	230.9	181.5	#C84150
187	High-middle SDI/1990	Deaths	High-middle SDI	Both	95+ years	Lip and oral cavity cancer	Number	1990	50.6	56.0	40.7	#C84150
188	High-middle SDI/2021	Deaths	High-middle SDI	Both	15-19 years	Lip and oral cavity cancer	Number	2021	19.4	21.9	16.8	#4484B0
189	High-middle SDI/2021	Deaths	High-middle SDI	Both	20-24 years	Lip and oral cavity cancer	Number	2021	36.3	40.6	31.9	#4484B0
190	High-middle SDI/2021	Deaths	High-middle SDI	Both	25-29 years	Lip and oral cavity cancer	Number	2021	72.3	82.4	63.2	#4484B0
191	High-middle SDI/2021	Deaths	High-middle SDI	Both	30-34 years	Lip and oral cavity cancer	Number	2021	200.5	222.7	179.7	#4484B0
192	High-middle SDI/2021	Deaths	High-middle SDI	Both	35-39 years	Lip and oral cavity cancer	Number	2021	401.0	438.3	366.5	#4484B0
193	High-middle SDI/2021	Deaths	High-middle SDI	Both	40-44 years	Lip and oral cavity cancer	Number	2021	829.5	905.5	757.8	#4484B0
194	High-middle SDI/2021	Deaths	High-middle SDI	Both	45-49 years	Lip and oral cavity cancer	Number	2021	1595.4	1761.0	1455.5	#4484B0
195	High-middle SDI/2021	Deaths	High-middle SDI	Both	50-54 years	Lip and oral cavity cancer	Number	2021	2704.5	2999.4	2447.1	#4484B0
196	High-middle SDI/2021	Deaths	High-middle SDI	Both	55-59 years	Lip and oral cavity cancer	Number	2021	3810.3	4214.1	3437.1	#4484B0

197	High-middle SDI/2021	Deaths	High-middle SDI	Both	60-64 years	Lip and oral cavity cancer	Number	2021	4412.9	4798.9	4024.6	#4484B0
198	High-middle SDI/2021	Deaths	High-middle SDI	Both	65-69 years	Lip and oral cavity cancer	Number	2021	4589.8	5067.7	4155.3	#4484B0
199	High-middle SDI/2021	Deaths	High-middle SDI	Both	70-74 years	Lip and oral cavity cancer	Number	2021	3941.6	4342.4	3539.9	#4484B0
200	High-middle SDI/2021	Deaths	High-middle SDI	Both	75-79 years	Lip and oral cavity cancer	Number	2021	2817.5	3120.5	2504.0	#4484B0
201	High-middle SDI/2021	Deaths	High-middle SDI	Both	80-84 years	Lip and oral cavity cancer	Number	2021	2619.2	2854.2	2311.4	#4484B0
202	High-middle SDI/2021	Deaths	High-middle SDI	Both	85-89 years	Lip and oral cavity cancer	Number	2021	1887.5	2081.8	1611.1	#4484B0
203	High-middle SDI/2021	Deaths	High-middle SDI	Both	90-94 years	Lip and oral cavity cancer	Number	2021	956.4	1057.5	778.2	#4484B0
204	High-middle SDI/2021	Deaths	High-middle SDI	Both	95+ years	Lip and oral cavity cancer	Number	2021	302.7	340.8	228.5	#4484B0
205	High SDI/1990	Deaths	High SDI	Both	15-19 years	Lip and oral cavity cancer	Number	1990	17.9	18.6	17.4	#8F243D
206	High SDI/1990	Deaths	High SDI	Both	20-24 years	Lip and oral cavity cancer	Number	1990	34.6	36.0	33.4	#8F243D
207	High SDI/1990	Deaths	High SDI	Both	25-29 years	Lip and oral cavity cancer	Number	1990	67.2	69.9	64.9	#8F243D
208	High SDI/1990	Deaths	High SDI	Both	30-34 years	Lip and oral cavity cancer	Number	1990	136.5	141.7	131.4	#8F243D
209	High SDI/1990	Deaths	High SDI	Both	35-39 years	Lip and oral cavity cancer	Number	1990	305.5	316.1	294.7	#8F243D
210	High SDI/1990	Deaths	High SDI	Both	40-44 years	Lip and oral cavity cancer	Number	1990	674.6	704.3	648.1	#8F243D
211	High SDI/1990	Deaths	High SDI	Both	45-49 years	Lip and oral cavity cancer	Number	1990	1172.9	1233.4	1113.4	#8F243D
212	High SDI/1990	Deaths	High SDI	Both	50-54 years	Lip and oral cavity cancer	Number	1990	1810.1	1921.6	1719.0	#8F243D
213	High SDI/1990	Deaths	High SDI	Both	55-59 years	Lip and oral cavity cancer	Number	1990	2403.6	2537.9	2285.9	#8F243D
214	High SDI/1990	Deaths	High SDI	Both	60-64 years	Lip and oral cavity cancer	Number	1990	2881.9	3001.6	2768.1	#8F243D
215	High SDI/1990	Deaths	High SDI	Both	65-69 years	Lip and oral cavity cancer	Number	1990	2890.8	3004.8	2782.9	#8F243D
216	High SDI/1990	Deaths	High SDI	Both	70-74 years	Lip and oral cavity cancer	Number	1990	2299.4	2385.2	2190.8	#8F243D
217	High SDI/1990	Deaths	High SDI	Both	75-79 years	Lip and oral cavity cancer	Number	1990	2388.3	2475.7	2262.8	#8F243D
218	High SDI/1990	Deaths	High SDI	Both	80-84 years	Lip and oral cavity cancer	Number	1990	1867.1	1960.9	1686.5	#8F243D

219	High SDI/1990	Deaths	High SDI	Both	85-89 years	Lip and oral cavity cancer	Number	1990	1183.8	1265.6	1015.3	#8F243D
220	High SDI/1990	Deaths	High SDI	Both	90-94 years	Lip and oral cavity cancer	Number	1990	520.1	567.9	423.1	#8F243D
221	High SDI/1990	Deaths	High SDI	Both	95+ years	Lip and oral cavity cancer	Number	1990	158.0	178.9	118.0	#8F243D
222	High SDI/2021	Deaths	High SDI	Both	15-19 years	Lip and oral cavity cancer	Number	2021	9.7	10.3	9.1	#6752A9
223	High SDI/2021	Deaths	High SDI	Both	20-24 years	Lip and oral cavity cancer	Number	2021	25.7	27.2	24.4	#6752A9
224	High SDI/2021	Deaths	High SDI	Both	25-29 years	Lip and oral cavity cancer	Number	2021	53.4	56.6	50.9	#6752A9
225	High SDI/2021	Deaths	High SDI	Both	30-34 years	Lip and oral cavity cancer	Number	2021	112.4	120.1	106.9	#6752A9
226	High SDI/2021	Deaths	High SDI	Both	35-39 years	Lip and oral cavity cancer	Number	2021	237.2	249.1	225.8	#6752A9
227	High SDI/2021	Deaths	High SDI	Both	40-44 years	Lip and oral cavity cancer	Number	2021	502.6	533.1	475.4	#6752A9
228	High SDI/2021	Deaths	High SDI	Both	45-49 years	Lip and oral cavity cancer	Number	2021	952.4	1002.0	904.2	#6752A9
229	High SDI/2021	Deaths	High SDI	Both	50-54 years	Lip and oral cavity cancer	Number	2021	1738.9	1823.0	1653.3	#6752A9
230	High SDI/2021	Deaths	High SDI	Both	55-59 years	Lip and oral cavity cancer	Number	2021	2804.1	2955.8	2660.6	#6752A9
231	High SDI/2021	Deaths	High SDI	Both	60-64 years	Lip and oral cavity cancer	Number	2021	3665.5	3826.2	3502.9	#6752A9
232	High SDI/2021	Deaths	High SDI	Both	65-69 years	Lip and oral cavity cancer	Number	2021	4011.8	4199.5	3831.1	#6752A9
233	High SDI/2021	Deaths	High SDI	Both	70-74 years	Lip and oral cavity cancer	Number	2021	4090.8	4283.9	3855.6	#6752A9
234	High SDI/2021	Deaths	High SDI	Both	75-79 years	Lip and oral cavity cancer	Number	2021	3556.6	3732.9	3223.3	#6752A9
235	High SDI/2021	Deaths	High SDI	Both	80-84 years	Lip and oral cavity cancer	Number	2021	3460.1	3762.9	2892.8	#6752A9
236	High SDI/2021	Deaths	High SDI	Both	85-89 years	Lip and oral cavity cancer	Number	2021	3161.7	3515.4	2498.5	#6752A9
237	High SDI/2021	Deaths	High SDI	Both	90-94 years	Lip and oral cavity cancer	Number	2021	2274.0	2580.3	1732.8	#6752A9
238	High SDI/2021	Deaths	High SDI	Both	95+ years	Lip and oral cavity cancer	Number	2021	1091.3	1274.6	759.0	#6752A9
239	Low-middle SDI/1990	Deaths	Low-middle SDI	Both	15-19 years	Lip and oral cavity cancer	Number	1990	149.3	174.8	127.7	#FDAD6B
240	Low-middle SDI/1990	Deaths	Low-middle SDI	Both	20-24 years	Lip and oral cavity cancer	Number	1990	242.6	275.7	211.0	#FDAD6B

241	Low-middle SDI/1990	Deaths	Low-middle SDI	Both	25-29 years	Lip and oral cavity cancer	Number	1990	340.8	397.3	289.6	#FDAD6B
242	Low-middle SDI/1990	Deaths	Low-middle SDI	Both	30-34 years	Lip and oral cavity cancer	Number	1990	537.7	624.9	456.6	#FDAD6B
243	Low-middle SDI/1990	Deaths	Low-middle SDI	Both	35-39 years	Lip and oral cavity cancer	Number	1990	878.6	1019.2	743.3	#FDAD6B
244	Low-middle SDI/1990	Deaths	Low-middle SDI	Both	40-44 years	Lip and oral cavity cancer	Number	1990	1480.0	1676.5	1274.2	#FDAD6B
245	Low-middle SDI/1990	Deaths	Low-middle SDI	Both	45-49 years	Lip and oral cavity cancer	Number	1990	2085.5	2375.5	1856.8	#FDAD6B
246	Low-middle SDI/1990	Deaths	Low-middle SDI	Both	50-54 years	Lip and oral cavity cancer	Number	1990	3020.6	3473.9	2637.5	#FDAD6B
247	Low-middle SDI/1990	Deaths	Low-middle SDI	Both	55-59 years	Lip and oral cavity cancer	Number	1990	3494.9	4037.0	3110.1	#FDAD6B
248	Low-middle SDI/1990	Deaths	Low-middle SDI	Both	60-64 years	Lip and oral cavity cancer	Number	1990	4085.4	4675.8	3573.5	#FDAD6B
249	Low-middle SDI/1990	Deaths	Low-middle SDI	Both	65-69 years	Lip and oral cavity cancer	Number	1990	3664.1	4203.9	3149.6	#FDAD6B
250	Low-middle SDI/1990	Deaths	Low-middle SDI	Both	70-74 years	Lip and oral cavity cancer	Number	1990	2776.6	3170.7	2426.4	#FDAD6B
251	Low-middle SDI/1990	Deaths	Low-middle SDI	Both	75-79 years	Lip and oral cavity cancer	Number	1990	1994.1	2261.1	1692.2	#FDAD6B
252	Low-middle SDI/1990	Deaths	Low-middle SDI	Both	80-84 years	Lip and oral cavity cancer	Number	1990	1155.6	1334.0	970.9	#FDAD6B
253	Low-middle SDI/1990	Deaths	Low-middle SDI	Both	85-89 years	Lip and oral cavity cancer	Number	1990	543.4	637.0	456.4	#FDAD6B
254	Low-middle SDI/1990	Deaths	Low-middle SDI	Both	90-94 years	Lip and oral cavity cancer	Number	1990	180.6	213.4	151.4	#FDAD6B
255	Low-middle SDI/1990	Deaths	Low-middle SDI	Both	95+ years	Lip and oral cavity cancer	Number	1990	42.6	52.0	33.7	#FDAD6B
256	Low-middle SDI/2021	Deaths	Low-middle SDI	Both	15-19 years	Lip and oral cavity cancer	Number	2021	265.5	348.1	190.1	#BCF0AC
257	Low-middle SDI/2021	Deaths	Low-middle SDI	Both	20-24 years	Lip and oral cavity cancer	Number	2021	497.3	653.7	351.2	#BCF0AC
258	Low-middle SDI/2021	Deaths	Low-middle SDI	Both	25-29 years	Lip and oral cavity cancer	Number	2021	723.6	918.6	538.3	#BCF0AC
259	Low-middle SDI/2021	Deaths	Low-middle SDI	Both	30-34 years	Lip and oral cavity cancer	Number	2021	1222.1	1513.7	936.3	#BCF0AC
260	Low-middle SDI/2021	Deaths	Low-middle SDI	Both	35-39 years	Lip and oral cavity cancer	Number	2021	1951.2	2332.5	1549.5	#BCF0AC
261	Low-middle SDI/2021	Deaths	Low-middle SDI	Both	40-44 years	Lip and oral cavity cancer	Number	2021	3506.4	4055.3	2923.9	#BCF0AC
262	Low-middle SDI/2021	Deaths	Low-middle SDI	Both	45-49 years	Lip and oral cavity cancer	Number	2021	4753.5	5484.8	3930.4	#BCF0AC

263	Low-middle SDI/2021	Deaths	Low-middle SDI	Both	50-54 years	Lip and oral cavity cancer	Number	2021	6929.4	7967.3	5918.6	#BCF0AC
264	Low-middle SDI/2021	Deaths	Low-middle SDI	Both	55-59 years	Lip and oral cavity cancer	Number	2021	8276.3	9468.0	7146.6	#BCF0AC
265	Low-middle SDI/2021	Deaths	Low-middle SDI	Both	60-64 years	Lip and oral cavity cancer	Number	2021	9240.9	10598.4	7906.7	#BCF0AC
266	Low-middle SDI/2021	Deaths	Low-middle SDI	Both	65-69 years	Lip and oral cavity cancer	Number	2021	9136.1	10341.9	7992.5	#BCF0AC
267	Low-middle SDI/2021	Deaths	Low-middle SDI	Both	70-74 years	Lip and oral cavity cancer	Number	2021	7916.0	8841.5	6856.4	#BCF0AC
268	Low-middle SDI/2021	Deaths	Low-middle SDI	Both	75-79 years	Lip and oral cavity cancer	Number	2021	5845.3	6535.2	5133.5	#BCF0AC
269	Low-middle SDI/2021	Deaths	Low-middle SDI	Both	80-84 years	Lip and oral cavity cancer	Number	2021	3862.7	4367.5	3370.6	#BCF0AC
270	Low-middle SDI/2021	Deaths	Low-middle SDI	Both	85-89 years	Lip and oral cavity cancer	Number	2021	2099.5	2412.5	1790.2	#BCF0AC
271	Low-middle SDI/2021	Deaths	Low-middle SDI	Both	90-94 years	Lip and oral cavity cancer	Number	2021	898.1	1029.4	742.3	#BCF0AC
272	Low-middle SDI/2021	Deaths	Low-middle SDI	Both	95+ years	Lip and oral cavity cancer	Number	2021	236.9	278.8	185.1	#BCF0AC
273	Low SDI/1990	Deaths	Low SDI	Both	15-19 years	Lip and oral cavity cancer	Number	1990	47.9	59.7	38.8	#FADA93
274	Low SDI/1990	Deaths	Low SDI	Both	20-24 years	Lip and oral cavity cancer	Number	1990	65.7	81.2	53.4	#FADA93
275	Low SDI/1990	Deaths	Low SDI	Both	25-29 years	Lip and oral cavity cancer	Number	1990	89.6	109.0	71.4	#FADA93
276	Low SDI/1990	Deaths	Low SDI	Both	30-34 years	Lip and oral cavity cancer	Number	1990	139.3	169.1	111.2	#FADA93
277	Low SDI/1990	Deaths	Low SDI	Both	35-39 years	Lip and oral cavity cancer	Number	1990	232.9	278.8	186.9	#FADA93
278	Low SDI/1990	Deaths	Low SDI	Both	40-44 years	Lip and oral cavity cancer	Number	1990	374.5	435.4	311.2	#FADA93
279	Low SDI/1990	Deaths	Low SDI	Both	45-49 years	Lip and oral cavity cancer	Number	1990	544.3	635.6	462.0	#FADA93
280	Low SDI/1990	Deaths	Low SDI	Both	50-54 years	Lip and oral cavity cancer	Number	1990	830.8	988.5	700.1	#FADA93
281	Low SDI/1990	Deaths	Low SDI	Both	55-59 years	Lip and oral cavity cancer	Number	1990	1016.8	1176.8	875.7	#FADA93
282	Low SDI/1990	Deaths	Low SDI	Both	60-64 years	Lip and oral cavity cancer	Number	1990	1136.3	1313.3	962.0	#FADA93
283	Low SDI/1990	Deaths	Low SDI	Both	65-69 years	Lip and oral cavity cancer	Number	1990	1023.2	1185.4	864.2	#FADA93
284	Low SDI/1990	Deaths	Low SDI	Both	70-74 years	Lip and oral cavity cancer	Number	1990	793.0	924.5	667.0	#FADA93

285	Low SDI/1990	Deaths	Low SDI	Both	75-79 years	Lip and oral cavity cancer	Number	1990	532.5	621.5	453.5	#FADA93
286	Low SDI/1990	Deaths	Low SDI	Both	80-84 years	Lip and oral cavity cancer	Number	1990	298.3	345.6	254.1	#FADA93
287	Low SDI/1990	Deaths	Low SDI	Both	85-89 years	Lip and oral cavity cancer	Number	1990	113.6	131.5	95.4	#FADA93
288	Low SDI/1990	Deaths	Low SDI	Both	90-94 years	Lip and oral cavity cancer	Number	1990	32.5	38.7	27.1	#FADA93
289	Low SDI/1990	Deaths	Low SDI	Both	95+ years	Lip and oral cavity cancer	Number	1990	7.6	9.6	5.8	#FADA93
290	Low SDI/2021	Deaths	Low SDI	Both	15-19 years	Lip and oral cavity cancer	Number	2021	118.5	153.9	89.2	#EFF5A4
291	Low SDI/2021	Deaths	Low SDI	Both	20-24 years	Lip and oral cavity cancer	Number	2021	175.4	238.1	129.5	#EFF5A4
292	Low SDI/2021	Deaths	Low SDI	Both	25-29 years	Lip and oral cavity cancer	Number	2021	228.2	285.0	175.0	#EFF5A4
293	Low SDI/2021	Deaths	Low SDI	Both	30-34 years	Lip and oral cavity cancer	Number	2021	368.5	469.2	279.2	#EFF5A4
294	Low SDI/2021	Deaths	Low SDI	Both	35-39 years	Lip and oral cavity cancer	Number	2021	575.9	712.7	451.6	#EFF5A4
295	Low SDI/2021	Deaths	Low SDI	Both	40-44 years	Lip and oral cavity cancer	Number	2021	929.9	1126.9	746.7	#EFF5A4
296	Low SDI/2021	Deaths	Low SDI	Both	45-49 years	Lip and oral cavity cancer	Number	2021	1305.8	1583.0	1043.9	#EFF5A4
297	Low SDI/2021	Deaths	Low SDI	Both	50-54 years	Lip and oral cavity cancer	Number	2021	1816.1	2171.2	1490.0	#EFF5A4
298	Low SDI/2021	Deaths	Low SDI	Both	55-59 years	Lip and oral cavity cancer	Number	2021	2154.0	2506.2	1823.1	#EFF5A4
299	Low SDI/2021	Deaths	Low SDI	Both	60-64 years	Lip and oral cavity cancer	Number	2021	2374.6	2815.4	1996.3	#EFF5A4
300	Low SDI/2021	Deaths	Low SDI	Both	65-69 years	Lip and oral cavity cancer	Number	2021	2264.1	2659.7	1890.8	#EFF5A4
301	Low SDI/2021	Deaths	Low SDI	Both	70-74 years	Lip and oral cavity cancer	Number	2021	1999.3	2359.6	1655.5	#EFF5A4
302	Low SDI/2021	Deaths	Low SDI	Both	75-79 years	Lip and oral cavity cancer	Number	2021	1424.1	1651.8	1202.8	#EFF5A4
303	Low SDI/2021	Deaths	Low SDI	Both	80-84 years	Lip and oral cavity cancer	Number	2021	971.7	1117.5	831.3	#EFF5A4
304	Low SDI/2021	Deaths	Low SDI	Both	85-89 years	Lip and oral cavity cancer	Number	2021	451.2	533.1	380.6	#EFF5A4
305	Low SDI/2021	Deaths	Low SDI	Both	90-94 years	Lip and oral cavity cancer	Number	2021	145.8	171.6	120.1	#EFF5A4
306	Low SDI/2021	Deaths	Low SDI	Both	95+ years	Lip and oral cavity cancer	Number	2021	30.9	37.9	24.3	#EFF5A4

307	Middle SDI/1990	Deaths	Middle SDI	Both	15-19 years	Lip and oral cavity cancer	Number	1990	105.5	117.4	96.2	#F06D49
308	Middle SDI/1990	Deaths	Middle SDI	Both	20-24 years	Lip and oral cavity cancer	Number	1990	168.5	185.4	153.8	#F06D49
309	Middle SDI/1990	Deaths	Middle SDI	Both	25-29 years	Lip and oral cavity cancer	Number	1990	251.0	276.7	230.3	#F06D49
310	Middle SDI/1990	Deaths	Middle SDI	Both	30-34 years	Lip and oral cavity cancer	Number	1990	425.0	464.3	390.5	#F06D49
311	Middle SDI/1990	Deaths	Middle SDI	Both	35-39 years	Lip and oral cavity cancer	Number	1990	721.8	785.3	657.6	#F06D49
312	Middle SDI/1990	Deaths	Middle SDI	Both	40-44 years	Lip and oral cavity cancer	Number	1990	1143.1	1239.2	1058.0	#F06D49
313	Middle SDI/1990	Deaths	Middle SDI	Both	45-49 years	Lip and oral cavity cancer	Number	1990	1704.8	1870.1	1575.9	#F06D49
314	Middle SDI/1990	Deaths	Middle SDI	Both	50-54 years	Lip and oral cavity cancer	Number	1990	2551.8	2772.7	2339.8	#F06D49
315	Middle SDI/1990	Deaths	Middle SDI	Both	55-59 years	Lip and oral cavity cancer	Number	1990	3128.7	3416.4	2896.3	#F06D49
316	Middle SDI/1990	Deaths	Middle SDI	Both	60-64 years	Lip and oral cavity cancer	Number	1990	3316.6	3594.7	3052.2	#F06D49
317	Middle SDI/1990	Deaths	Middle SDI	Both	65-69 years	Lip and oral cavity cancer	Number	1990	3008.6	3263.5	2771.9	#F06D49
318	Middle SDI/1990	Deaths	Middle SDI	Both	70-74 years	Lip and oral cavity cancer	Number	1990	2479.0	2667.0	2285.3	#F06D49
319	Middle SDI/1990	Deaths	Middle SDI	Both	75-79 years	Lip and oral cavity cancer	Number	1990	1909.2	2059.7	1749.8	#F06D49
320	Middle SDI/1990	Deaths	Middle SDI	Both	80-84 years	Lip and oral cavity cancer	Number	1990	1223.7	1313.0	1122.5	#F06D49
321	Middle SDI/1990	Deaths	Middle SDI	Both	85-89 years	Lip and oral cavity cancer	Number	1990	651.5	710.6	586.5	#F06D49
322	Middle SDI/1990	Deaths	Middle SDI	Both	90-94 years	Lip and oral cavity cancer	Number	1990	209.5	233.7	180.9	#F06D49
323	Middle SDI/1990	Deaths	Middle SDI	Both	95+ years	Lip and oral cavity cancer	Number	1990	49.6	59.4	39.9	#F06D49
324	Middle SDI/2021	Deaths	Middle SDI	Both	15-19 years	Lip and oral cavity cancer	Number	2021	92.7	108.3	73.9	#7ED4B6
325	Middle SDI/2021	Deaths	Middle SDI	Both	20-24 years	Lip and oral cavity cancer	Number	2021	180.4	207.7	149.4	#7ED4B6
326	Middle SDI/2021	Deaths	Middle SDI	Both	25-29 years	Lip and oral cavity cancer	Number	2021	339.8	392.9	282.0	#7ED4B6
327	Middle SDI/2021	Deaths	Middle SDI	Both	30-34 years	Lip and oral cavity cancer	Number	2021	696.1	785.6	600.4	#7ED4B6
328	Middle SDI/2021	Deaths	Middle SDI	Both	35-39 years	Lip and oral cavity cancer	Number	2021	1207.9	1362.6	1035.5	#7ED4B6

329	Middle SDI/2021	Deaths	Middle SDI	Both	40-44 years	Lip and oral cavity cancer	Number	2021	2291.4	2615.0	1968.7	#7ED4B6
330	Middle SDI/2021	Deaths	Middle SDI	Both	45-49 years	Lip and oral cavity cancer	Number	2021	3775.2	4244.6	3300.9	#7ED4B6
331	Middle SDI/2021	Deaths	Middle SDI	Both	50-54 years	Lip and oral cavity cancer	Number	2021	5856.1	6551.0	5144.1	#7ED4B6
332	Middle SDI/2021	Deaths	Middle SDI	Both	55-59 years	Lip and oral cavity cancer	Number	2021	7554.2	8500.7	6711.6	#7ED4B6
333	Middle SDI/2021	Deaths	Middle SDI	Both	60-64 years	Lip and oral cavity cancer	Number	2021	8013.4	8867.8	7181.1	#7ED4B6
334	Middle SDI/2021	Deaths	Middle SDI	Both	65-69 years	Lip and oral cavity cancer	Number	2021	8565.0	9568.9	7601.0	#7ED4B6
335	Middle SDI/2021	Deaths	Middle SDI	Both	70-74 years	Lip and oral cavity cancer	Number	2021	7349.1	8229.3	6533.5	#7ED4B6
336	Middle SDI/2021	Deaths	Middle SDI	Both	75-79 years	Lip and oral cavity cancer	Number	2021	5826.3	6443.8	5246.0	#7ED4B6
337	Middle SDI/2021	Deaths	Middle SDI	Both	80-84 years	Lip and oral cavity cancer	Number	2021	4333.0	4757.1	3801.7	#7ED4B6
338	Middle SDI/2021	Deaths	Middle SDI	Both	85-89 years	Lip and oral cavity cancer	Number	2021	2864.6	3212.2	2449.6	#7ED4B6
339	Middle SDI/2021	Deaths	Middle SDI	Both	90-94 years	Lip and oral cavity cancer	Number	2021	1260.3	1417.8	1039.0	#7ED4B6
340	Middle SDI/2021	Deaths	Middle SDI	Both	95+ years	Lip and oral cavity cancer	Number	2021	360.3	421.3	272.2	#7ED4B6

Table S2. Country-level ranking and stratum of the Quality of Care Index for lip and oral cavity cancer among individuals aged 15 years and older, 2021.

	Country	val	val2
1	Australia	100.0	90-100
2	Spain	98.5	90-100
3	United States of America	95.7	90-100
4	France	95.4	90-100
5	New Zealand	95.2	90-100
6	United Kingdom	92.3	90-100
7	Iceland	91.5	90-100
8	Canada	90.3	90-100
9	Ireland	89.6	80-90
10	San Marino	89.3	80-90
11	Monaco	88.8	80-90
12	Switzerland	88.2	80-90
13	Norway	88.2	80-90
14	Finland	88.1	80-90
15	Sweden	88.0	80-90
16	Portugal	87.9	80-90
17	Netherlands	87.7	80-90
18	Belgium	86.9	80-90
19	Luxembourg	86.8	80-90
20	Slovenia	86.7	80-90
21	Andorra	86.6	80-90
22	Malta	86.4	80-90
23	Denmark	84.8	80-90
24	Cyprus	84.5	80-90
25	Greece	83.8	80-90
26	Israel	82.7	80-90
27	Germany	82.7	80-90
28	Japan	81.9	80-90
29	Taiwan (Province of China)	81.9	80-90
30	Republic of Korea	80.7	80-90
31	Italy	79.9	70-80
32	Singapore	79.7	70-80
33	Croatia	79.6	70-80
34	Estonia	76.9	70-80
35	Austria	75.3	70-80
36	Bermuda	74.7	70-80
37	Kuwait	74.7	70-80
38	Bulgaria	74.0	70-80
39	Chile	72.7	70-80
40	Puerto Rico	70.3	70-80
41	Qatar	69.9	60-70

42	Lebanon	68.3	60-70
43	China	67.1	60-70
44	Hungary	65.4	60-70
45	Oman	64.7	60-70
46	Uruguay	64.5	60-70
47	Thailand	64.4	60-70
48	Montenegro	64.0	60-70
49	Turkey	63.8	60-70
50	Cook Islands	63.6	60-70
51	Iran (Islamic Republic of)	63.3	60-70
52	Saudi Arabia	62.8	60-70
53	Tunisia	62.6	60-70
54	Jordan	62.5	60-70
55	Serbia	62.5	60-70
56	Costa Rica	62.4	60-70
57	Cuba	61.9	60-70
58	Bahrain	61.7	60-70
59	Czechia	60.9	60-70
60	Slovakia	60.5	60-70
61	Guam	60.0	50-60
62	Sri Lanka	59.5	50-60
63	Ukraine	59.4	50-60
64	Colombia	59.0	50-60
65	Argentina	58.6	50-60
66	Albania	58.4	50-60
67	Viet Nam	58.2	50-60
68	Bosnia and Herzegovina	57.9	50-60
69	Greenland	57.7	50-60
70	Peru	57.5	50-60
71	Belarus	57.5	50-60
72	Syrian Arab Republic	57.4	50-60
73	Romania	57.3	50-60
74	Libya	57.3	50-60
75	Maldives	57.0	50-60
76	Panama	56.4	50-60
77	Russian Federation	56.1	50-60
78	Malaysia	56.1	50-60
79	Iraq	55.8	50-60
80	Northern Mariana Islands	54.3	50-60
81	North Macedonia	54.2	50-60
82	Algeria	53.5	50-60
83	Mauritius	53.5	50-60
84	Palestine	53.4	50-60
85	Barbados	52.5	50-60

86	El Salvador	52.3	50-60
87	Lithuania	52.3	50-60
88	United States Virgin Islands	51.6	50-60
89	Democratic People's Republic of Korea	50.9	50-60
90	Latvia	50.8	50-60
91	Brunei Darussalam	50.0	40-50
92	Antigua and Barbuda	49.6	40-50
93	United Arab Emirates	49.3	40-50
94	Jamaica	49.2	40-50
95	Armenia	49.2	40-50
96	Palau	48.4	40-50
97	Kazakhstan	48.0	40-50
98	American Samoa	48.0	40-50
99	Venezuela (Bolivarian Republic of)	47.9	40-50
100	Nicaragua	47.8	40-50
101	Mexico	47.8	40-50
102	Niue	47.4	40-50
103	Brazil	47.2	40-50
104	Ecuador	47.1	40-50
105	Egypt	46.7	40-50
106	Seychelles	46.4	40-50
107	Tokelau	46.1	40-50
108	Poland	45.9	40-50
109	Morocco	45.5	40-50
110	Trinidad and Tobago	45.4	40-50
111	Bahamas	44.5	40-50
112	Republic of Moldova	44.3	40-50
113	Paraguay	43.8	40-50
114	Cabo Verde	43.3	40-50
115	Saint Lucia	43.3	40-50
116	Kyrgyzstan	42.0	40-50
117	Tonga	41.6	40-50
118	Georgia	41.6	40-50
119	Belize	41.5	40-50
120	Saint Kitts and Nevis	40.8	40-50
121	Azerbaijan	40.8	40-50
122	Grenada	40.8	40-50
123	Samoa	38.6	30-40
124	Dominican Republic	37.3	30-40
125	Bangladesh	37.3	30-40
126	Sudan	37.1	30-40
127	Saint Vincent and the Grenadines	36.4	30-40
128	Sao Tome and Principe	36.3	30-40
129	Indonesia	36.2	30-40

130	Turkmenistan	35.6	30-40
131	Uzbekistan	35.4	30-40
132	Equatorial Guinea	35.1	30-40
133	Fiji	35.0	30-40
134	Bhutan	35.0	30-40
135	India	34.7	30-40
136	Guatemala	34.7	30-40
137	Mauritania	34.7	30-40
138	Dominica	34.1	30-40
139	South Africa	34.0	30-40
140	Tuvalu	33.8	30-40
141	Myanmar	33.7	30-40
142	Ghana	33.2	30-40
143	Suriname	33.1	30-40
144	Cambodia	32.8	30-40
145	Philippines	32.2	30-40
146	Bolivia (Plurinational State of)	31.7	30-40
147	Nauru	31.4	30-40
148	Nepal	30.6	30-40
149	Mongolia	30.5	30-40
150	Gabon	29.8	20-30
151	Honduras	29.7	20-30
152	Namibia	29.5	20-30
153	Yemen	29.2	20-30
154	Micronesia (Federated States of)	28.5	20-30
155	Timor-Leste	27.0	20-30
156	Tajikistan	26.4	20-30
157	Gambia	26.3	20-30
158	Kenya	26.2	20-30
159	Guyana	25.9	20-30
160	Senegal	25.3	20-30
161	Lao People's Democratic Republic	24.9	20-30
162	Liberia	24.6	20-30
163	Marshall Islands	24.4	20-30
164	Botswana	24.3	20-30
165	C 涔炴 e d'Ivoire	23.8	20-30
166	Solomon Islands	23.3	20-30
167	Nigeria	23.1	20-30
168	Togo	23.0	20-30
169	Cameroon	22.6	20-30
170	Congo	22.5	20-30
171	Vanuatu	22.4	20-30
172	Afghanistan	22.3	20-30
173	Papua New Guinea	21.8	20-30

174	United Republic of Tanzania	21.7	20-30
175	Djibouti	20.8	20-30
176	Comoros	20.4	20-30
177	Benin	20.1	20-30
178	Malawi	20.0	10 月 20 日
179	Uganda	19.8	10 月 20 日
180	Sierra Leone	19.7	10 月 20 日
181	Rwanda	19.6	10 月 20 日
182	Mali	19.3	10 月 20 日
183	Zambia	19.2	10 月 20 日
184	Eswatini	19.0	10 月 20 日
185	Ethiopia	18.8	10 月 20 日
186	Pakistan	18.8	10 月 20 日
187	Angola	18.7	10 月 20 日
188	Burkina Faso	17.7	10 月 20 日
189	Zimbabwe	17.6	10 月 20 日
190	Madagascar	17.1	10 月 20 日
191	Democratic Republic of the Congo	16.2	10 月 20 日
192	Guinea	14.2	10 月 20 日
193	South Sudan	14.0	10 月 20 日
194	Niger	13.5	10 月 20 日
195	Eritrea	11.9	10 月 20 日
196	Guinea-Bissau	11.1	10 月 20 日
197	Chad	11.0	10 月 20 日
198	Burundi	10.2	10 月 20 日
199	Lesotho	9.2	0-10
200	Mozambique	9.1	0-10
201	Kiribati	7.8	0-10
202	Haiti	5.9	0-10
203	Somalia	2.5	0-10
204	Central African Republic	0.0	0-10

Table S3. Age- and sex-specific Quality of Care Index and related component indicators for lip and oral cavity cancer among individuals aged 15 years and older, by global region and Socio-demographic Index, 2021.

	year	sex_name	age_name	location_name	QCI	PC1	PC2	PCA_score	MIR	PIR	YLR	DPR	weight_PC1	weight_PC2	var_PC1	var_PC2
1	2021	Male	15-19 years	High SDI	88.8	0.5	1.0	0.6	0.1	6.5	21.3	1.6	0.9	0.1	3.4	0.5
2	2021	Female	15-19 years	High SDI	94.9	1.1	0.6	1.0	0.1	6.6	15.6	1.2	0.9	0.1	3.4	0.5
3	2021	Both	15-19 years	High SDI	92.1	0.8	0.8	0.8	0.1	6.6	18.2	1.4	0.9	0.1	3.4	0.5
4	2021	Male	20-24 years	High SDI	90.7	0.7	0.9	0.7	0.1	6.6	19.5	1.5	0.9	0.1	3.4	0.5
5	2021	Female	20-24 years	High SDI	95.9	1.2	0.6	1.1	0.1	6.8	14.9	1.1	0.9	0.1	3.4	0.5
6	2021	Both	20-24 years	High SDI	93.3	0.9	0.7	0.9	0.1	6.7	17.2	1.3	0.9	0.1	3.4	0.5
7	2021	Male	25-29 years	High SDI	94.0	1.0	0.7	1.0	0.1	6.8	16.5	1.3	0.9	0.1	3.4	0.5
8	2021	Female	25-29 years	High SDI	98.4	1.4	0.4	1.3	0.1	6.9	12.5	1.0	0.9	0.1	3.4	0.5
9	2021	Both	25-29 years	High SDI	96.1	1.2	0.6	1.1	0.1	6.8	14.6	1.1	0.9	0.1	3.4	0.5
10	2021	Male	30-34 years	High SDI	94.8	1.1	0.6	1.0	0.1	6.8	15.4	1.2	0.9	0.1	3.4	0.5
11	2021	Female	30-34 years	High SDI	99.6	1.5	0.3	1.3	0.1	6.9	11.3	0.9	0.9	0.1	3.4	0.5
12	2021	Both	30-34 years	High SDI	96.8	1.3	0.5	1.2	0.1	6.9	13.7	1.1	0.9	0.1	3.4	0.5
13	2021	Male	35-39 years	High SDI	94.9	1.1	0.4	1.0	0.1	6.6	14.2	1.2	0.9	0.1	3.4	0.5
14	2021	Female	35-39 years	High SDI	100.0	1.6	0.2	1.4	0.1	6.8	10.1	0.8	0.9	0.1	3.4	0.5
15	2021	Both	35-39 years	High SDI	96.7	1.3	0.3	1.1	0.1	6.7	12.7	1.0	0.9	0.1	3.4	0.5
16	2021	Male	40-44 years	High SDI	86.6	0.4	0.7	0.4	0.2	6.1	19.8	1.6	0.9	0.1	3.4	0.5
17	2021	Female	40-44 years	High SDI	95.7	1.2	0.3	1.1	0.1	6.6	13.0	1.0	0.9	0.1	3.4	0.5
18	2021	Both	40-44 years	High SDI	89.5	0.7	0.5	0.6	0.2	6.3	17.6	1.5	0.9	0.1	3.4	0.5
19	2021	Male	45-49 years	High SDI	83.9	0.2	0.6	0.3	0.2	5.8	20.4	1.8	0.9	0.1	3.4	0.5
20	2021	Female	45-49	High SDI	93.4	1.0	0.2	0.9	0.2	6.3	13.6	1.2	0.9	0.1	3.4	0.5

			years													
21	2021	Both	45-49 years	High SDI	86.7	0.4	0.5	0.4	0.2	6.0	18.4	1.6	0.9	0.1	3.4	0.5
22	2021	Male	50-54 years	High SDI	83.0	0.2	0.4	0.2	0.3	5.7	19.7	1.8	0.9	0.1	3.4	0.5
23	2021	Female	50-54 years	High SDI	91.4	0.9	0.2	0.8	0.2	6.1	14.2	1.2	0.9	0.1	3.4	0.5
24	2021	Both	50-54 years	High SDI	85.3	0.4	0.4	0.4	0.2	5.8	18.2	1.6	0.9	0.1	3.4	0.5
25	2021	Male	55-59 years	High SDI	82.9	0.2	0.3	0.2	0.3	5.5	18.5	1.7	0.9	0.1	3.4	0.5
26	2021	Female	55-59 years	High SDI	91.3	0.9	0.1	0.8	0.2	6.0	13.3	1.2	0.9	0.1	3.4	0.5
27	2021	Both	55-59 years	High SDI	85.2	0.4	0.2	0.3	0.2	5.6	17.1	1.6	0.9	0.1	3.4	0.5
28	2021	Male	60-64 years	High SDI	81.7	0.1	0.1	0.1	0.3	5.2	17.8	1.7	0.9	0.1	3.4	0.5
29	2021	Female	60-64 years	High SDI	89.1	0.7	-0.1	0.6	0.2	5.7	13.4	1.2	0.9	0.1	3.4	0.5
30	2021	Both	60-64 years	High SDI	83.7	0.3	0.0	0.2	0.3	5.3	16.6	1.5	0.9	0.1	3.4	0.5
31	2021	Male	65-69 years	High SDI	81.8	0.2	-0.2	0.1	0.3	4.9	15.9	1.5	0.9	0.1	3.4	0.5
32	2021	Female	65-69 years	High SDI	89.5	0.8	-0.3	0.6	0.2	5.5	11.7	1.1	0.9	0.1	3.4	0.5
33	2021	Both	65-69 years	High SDI	84.1	0.3	-0.2	0.3	0.3	5.1	14.6	1.4	0.9	0.1	3.4	0.5
34	2021	Male	70-74 years	High SDI	82.2	0.2	-0.5	0.1	0.3	4.6	13.7	1.4	0.9	0.1	3.4	0.5
35	2021	Female	70-74 years	High SDI	88.7	0.8	-0.5	0.6	0.2	5.2	10.4	1.0	0.9	0.1	3.4	0.5
36	2021	Both	70-74 years	High SDI	84.3	0.4	-0.5	0.3	0.3	4.8	12.5	1.3	0.9	0.1	3.4	0.5
37	2021	Male	75-79 years	High SDI	80.4	0.1	-0.8	0.0	0.3	4.1	12.6	1.3	0.9	0.1	3.4	0.5
38	2021	Female	75-79 years	High SDI	86.0	0.6	-0.7	0.4	0.3	4.8	10.2	1.1	0.9	0.1	3.4	0.5
39	2021	Both	75-79 years	High SDI	82.5	0.3	-0.8	0.2	0.3	4.4	11.6	1.2	0.9	0.1	3.4	0.5
40	2021	Male	80-84 years	High SDI	74.6	-0.3	-1.1	-0.4	0.4	3.2	12.9	1.6	0.9	0.1	3.4	0.5
41	2021	Female	80-84 years	High SDI	77.9	0.0	-1.1	-0.2	0.4	3.7	11.7	1.3	0.9	0.1	3.4	0.5
42	2021	Both	80-84	High SDI	76.1	-0.2	-1.1	-0.3	0.4	3.4	12.4	1.5	0.9	0.1	3.4	0.5

			years													
43	2021	Male	85-89 years	High SDI	67.6	-0.8	-1.4	-0.9	0.4	2.3	14.0	2.1	0.9	0.1	3.4	0.5
44	2021	Female	85-89 years	High SDI	69.5	-0.6	-1.4	-0.7	0.5	2.6	13.4	1.8	0.9	0.1	3.4	0.5
45	2021	Both	85-89 years	High SDI	68.6	-0.7	-1.4	-0.8	0.4	2.5	13.7	1.9	0.9	0.1	3.4	0.5
46	2021	Male	90-94 years	High SDI	53.1	-2.0	-1.3	-1.9	0.6	1.5	19.7	3.7	0.9	0.1	3.4	0.5
47	2021	Female	90-94 years	High SDI	53.1	-2.0	-1.2	-1.9	0.6	1.6	20.1	3.5	0.9	0.1	3.4	0.5
48	2021	Both	90-94 years	High SDI	53.1	-2.0	-1.3	-1.9	0.6	1.5	20.0	3.6	0.9	0.1	3.4	0.5
49	2021	Male	95+ years	High SDI	0.9	-6.5	1.0	-5.5	1.0	0.5	43.6	16.4	0.9	0.1	3.4	0.5
50	2021	Female	95+ years	High SDI	0.0	-6.6	1.0	-5.6	1.0	0.5	43.8	16.6	0.9	0.1	3.4	0.5
51	2021	Both	95+ years	High SDI	0.2	-6.6	1.0	-5.5	1.0	0.5	43.8	16.5	0.9	0.1	3.4	0.5
52	2021	Male	15-19 years	Global	0.0	-0.9	-2.7	-1.5	0.4	4.8	78.4	6.7	0.7	0.3	2.6	1.3
53	2021	Female	15-19 years	Global	3.0	-1.3	-1.6	-1.4	0.4	5.2	62.6	5.2	0.7	0.3	2.6	1.3
54	2021	Both	15-19 years	Global	1.8	-1.1	-2.0	-1.4	0.4	5.0	69.1	5.8	0.7	0.3	2.6	1.3
55	2021	Male	20-24 years	Global	4.0	-0.9	-2.1	-1.3	0.4	4.9	70.1	6.1	0.7	0.3	2.6	1.3
56	2021	Female	20-24 years	Global	4.8	-1.2	-1.4	-1.3	0.4	5.1	60.8	5.0	0.7	0.3	2.6	1.3
57	2021	Both	20-24 years	Global	4.4	-1.1	-1.7	-1.3	0.4	5.0	64.8	5.5	0.7	0.3	2.6	1.3
58	2021	Male	25-29 years	Global	6.3	-1.3	-1.1	-1.2	0.4	5.4	55.9	4.6	0.7	0.3	2.6	1.3
59	2021	Female	25-29 years	Global	7.3	-1.6	-0.4	-1.2	0.3	5.7	45.9	3.7	0.7	0.3	2.6	1.3
60	2021	Both	25-29 years	Global	6.8	-1.5	-0.8	-1.2	0.4	5.5	51.0	4.1	0.7	0.3	2.6	1.3
61	2021	Male	30-34 years	Global	10.0	-1.4	-0.5	-1.1	0.4	5.5	46.8	3.9	0.7	0.3	2.6	1.3
62	2021	Female	30-34 years	Global	9.6	-1.7	0.1	-1.1	0.3	5.8	38.3	3.1	0.7	0.3	2.6	1.3
63	2021	Both	30-34 years	Global	9.8	-1.5	-0.3	-1.1	0.3	5.6	43.1	3.5	0.7	0.3	2.6	1.3
64	2021	Male	35-39 years	Global	16.2	-1.3	0.0	-0.8	0.3	5.1	40.4	3.6	0.7	0.3	2.6	1.3
65	2021	Female	35-39 years	Global	14.0	-1.7	0.5	-0.9	0.3	5.5	32.9	2.7	0.7	0.3	2.6	1.3

66	2021	Both	35-39 years	Global	15.4	-1.4	0.2	-0.9	0.3	5.2	37.7	3.2	0.7	0.3	2.6	1.3
67	2021	Male	40-44 years	Global	21.0	-0.6	-0.7	-0.6	0.4	4.3	50.0	4.8	0.7	0.3	2.6	1.3
68	2021	Female	40-44 years	Global	18.6	-1.1	0.0	-0.7	0.4	4.9	40.4	3.6	0.7	0.3	2.6	1.3
69	2021	Both	40-44 years	Global	20.2	-0.8	-0.4	-0.7	0.4	4.5	46.7	4.4	0.7	0.3	2.6	1.3
70	2021	Male	45-49 years	Global	25.6	-0.4	-0.6	-0.5	0.5	4.1	48.5	4.9	0.7	0.3	2.6	1.3
71	2021	Female	45-49 years	Global	23.1	-0.9	0.0	-0.6	0.4	4.6	40.0	3.8	0.7	0.3	2.6	1.3
72	2021	Both	45-49 years	Global	24.8	-0.6	-0.3	-0.5	0.4	4.2	45.7	4.5	0.7	0.3	2.6	1.3
73	2021	Male	50-54 years	Global	29.7	-0.3	-0.3	-0.3	0.5	3.9	45.2	4.7	0.7	0.3	2.6	1.3
74	2021	Female	50-54 years	Global	26.4	-0.7	0.1	-0.4	0.4	4.4	39.3	3.7	0.7	0.3	2.6	1.3
75	2021	Both	50-54 years	Global	28.7	-0.4	-0.2	-0.3	0.5	4.1	43.4	4.4	0.7	0.3	2.6	1.3
76	2021	Male	55-59 years	Global	33.0	-0.3	0.1	-0.2	0.5	3.9	40.3	4.2	0.7	0.3	2.6	1.3
77	2021	Female	55-59 years	Global	30.3	-0.6	0.4	-0.3	0.4	4.3	35.8	3.5	0.7	0.3	2.6	1.3
78	2021	Both	55-59 years	Global	32.1	-0.4	0.2	-0.2	0.5	4.0	38.9	4.0	0.7	0.3	2.6	1.3
79	2021	Male	60-64 years	Global	38.2	-0.1	0.3	0.0	0.5	3.6	37.7	4.1	0.7	0.3	2.6	1.3
80	2021	Female	60-64 years	Global	35.4	-0.3	0.5	-0.1	0.5	3.9	34.9	3.6	0.7	0.3	2.6	1.3
81	2021	Both	60-64 years	Global	37.3	-0.2	0.3	0.0	0.5	3.7	36.8	3.9	0.7	0.3	2.6	1.3
82	2021	Male	65-69 years	Global	42.8	0.0	0.6	0.2	0.5	3.4	32.9	3.7	0.7	0.3	2.6	1.3
83	2021	Female	65-69 years	Global	39.7	-0.3	0.9	0.1	0.5	3.9	29.3	3.1	0.7	0.3	2.6	1.3
84	2021	Both	65-69 years	Global	41.8	-0.1	0.7	0.2	0.5	3.6	31.7	3.5	0.7	0.3	2.6	1.3
85	2021	Male	70-74 years	Global	47.6	0.1	1.0	0.4	0.5	3.2	28.2	3.3	0.7	0.3	2.6	1.3
86	2021	Female	70-74 years	Global	44.6	-0.2	1.2	0.3	0.5	3.6	25.1	2.8	0.7	0.3	2.6	1.3
87	2021	Both	70-74 years	Global	46.6	0.0	1.1	0.4	0.5	3.4	27.1	3.1	0.7	0.3	2.6	1.3

88	2021	Male	75-79 years	Global	53.8	0.4	1.2	0.7	0.6	2.9	25.4	3.2	0.7	0.3	2.6	1.3
89	2021	Female	75-79 years	Global	50.7	0.1	1.4	0.6	0.5	3.3	22.5	2.6	0.7	0.3	2.6	1.3
90	2021	Both	75-79 years	Global	52.7	0.3	1.3	0.6	0.5	3.0	24.3	3.0	0.7	0.3	2.6	1.3
91	2021	Male	80-84 years	Global	62.2	0.8	1.4	1.0	0.6	2.3	23.9	3.4	0.7	0.3	2.6	1.3
92	2021	Female	80-84 years	Global	60.5	0.7	1.5	1.0	0.6	2.6	22.6	3.1	0.7	0.3	2.6	1.3
93	2021	Both	80-84 years	Global	61.4	0.8	1.4	1.0	0.6	2.4	23.3	3.3	0.7	0.3	2.6	1.3
94	2021	Male	85-89 years	Global	70.2	1.3	1.4	1.3	0.7	1.7	23.3	4.0	0.7	0.3	2.6	1.3
95	2021	Female	85-89 years	Global	68.3	1.1	1.6	1.3	0.7	2.0	21.6	3.4	0.7	0.3	2.6	1.3
96	2021	Both	85-89 years	Global	69.3	1.2	1.5	1.3	0.7	1.8	22.4	3.7	0.7	0.3	2.6	1.3
97	2021	Male	90-94 years	Global	80.0	2.2	0.8	1.7	0.8	1.2	28.6	6.2	0.7	0.3	2.6	1.3
98	2021	Female	90-94 years	Global	78.3	2.0	1.1	1.7	0.8	1.3	26.7	5.3	0.7	0.3	2.6	1.3
99	2021	Both	90-94 years	Global	79.0	2.1	1.0	1.7	0.8	1.2	27.5	5.7	0.7	0.3	2.6	1.3
100	2021	Male	95+ years	Global	100.0	5.1	-2.2	2.6	1.2	0.5	49.3	18.9	0.7	0.3	2.6	1.3
101	2021	Female	95+ years	Global	99.5	5.0	-2.0	2.5	1.1	0.5	48.1	18.5	0.7	0.3	2.6	1.3
102	2021	Both	95+ years	Global	99.6	5.0	-2.1	2.5	1.1	0.5	48.4	18.6	0.7	0.3	2.6	1.3
103	2021	Male	15-19 years	Low SDI	0.0	-2.0	-2.4	-2.1	0.6	4.2	103.8	9.5	0.8	0.2	3.0	0.8
104	2021	Female	15-19 years	Low SDI	7.5	-2.0	-0.8	-1.7	0.5	4.6	81.7	7.2	0.8	0.2	3.0	0.8
105	2021	Both	15-19 years	Low SDI	4.1	-2.0	-1.5	-1.9	0.5	4.5	91.6	8.2	0.8	0.2	3.0	0.8
106	2021	Male	20-24 years	Low SDI	4.2	-1.8	-2.0	-1.9	0.6	4.2	98.0	9.2	0.8	0.2	3.0	0.8
107	2021	Female	20-24 years	Low SDI	9.3	-1.9	-0.6	-1.6	0.5	4.6	79.7	6.9	0.8	0.2	3.0	0.8
108	2021	Both	20-24 years	Low SDI	7.3	-1.9	-1.2	-1.7	0.5	4.4	86.8	7.8	0.8	0.2	3.0	0.8
109	2021	Male	25-29 years	Low SDI	9.9	-1.9	-0.6	-1.6	0.5	4.8	79.2	6.9	0.8	0.2	3.0	0.8
110	2021	Female	25-29 years	Low SDI	14.2	-1.9	0.5	-1.4	0.4	5.1	64.4	5.4	0.8	0.2	3.0	0.8
111	2021	Both	25-29	Low SDI	12.1	-1.9	0.0	-1.5	0.5	4.9	71.5	6.1	0.8	0.2	3.0	0.8

			years													
112	2021	Male	30-34 years	Low SDI	14.9	-1.8	0.0	-1.4	0.5	4.8	70.5	6.2	0.8	0.2	3.0	0.8
113	2021	Female	30-34 years	Low SDI	17.9	-1.9	1.0	-1.2	0.4	5.1	57.0	4.8	0.8	0.2	3.0	0.8
114	2021	Both	30-34 years	Low SDI	16.4	-1.8	0.5	-1.3	0.5	5.0	63.8	5.5	0.8	0.2	3.0	0.8
115	2021	Male	35-39 years	Low SDI	22.8	-1.4	0.5	-1.0	0.5	4.2	63.0	6.1	0.8	0.2	3.0	0.8
116	2021	Female	35-39 years	Low SDI	23.9	-1.6	1.4	-0.9	0.4	4.7	51.8	4.6	0.8	0.2	3.0	0.8
117	2021	Both	35-39 years	Low SDI	23.2	-1.5	0.9	-1.0	0.4	4.4	58.1	5.4	0.8	0.2	3.0	0.8
118	2021	Male	40-44 years	Low SDI	24.8	-1.0	-0.6	-0.9	0.6	3.3	77.0	8.6	0.8	0.2	3.0	0.8
119	2021	Female	40-44 years	Low SDI	26.6	-1.2	0.6	-0.8	0.5	3.9	61.4	6.2	0.8	0.2	3.0	0.8
120	2021	Both	40-44 years	Low SDI	25.5	-1.1	-0.1	-0.9	0.5	3.5	70.5	7.5	0.8	0.2	3.0	0.8
121	2021	Male	45-49 years	Low SDI	28.5	-0.7	-0.9	-0.7	0.6	2.9	79.3	9.6	0.8	0.2	3.0	0.8
122	2021	Female	45-49 years	Low SDI	30.3	-0.9	0.4	-0.6	0.5	3.4	63.8	7.0	0.8	0.2	3.0	0.8
123	2021	Both	45-49 years	Low SDI	29.3	-0.8	-0.4	-0.7	0.6	3.1	72.9	8.5	0.8	0.2	3.0	0.8
124	2021	Male	50-54 years	Low SDI	32.9	-0.5	-0.7	-0.5	0.7	2.8	76.3	9.7	0.8	0.2	3.0	0.8
125	2021	Female	50-54 years	Low SDI	33.1	-0.7	0.2	-0.5	0.6	3.1	65.3	7.4	0.8	0.2	3.0	0.8
126	2021	Both	50-54 years	Low SDI	32.9	-0.6	-0.3	-0.5	0.7	2.9	72.1	8.7	0.8	0.2	3.0	0.8
127	2021	Male	55-59 years	Low SDI	37.6	-0.3	-0.4	-0.3	0.7	2.6	72.2	9.5	0.8	0.2	3.0	0.8
128	2021	Female	55-59 years	Low SDI	37.2	-0.5	0.4	-0.3	0.6	3.0	62.3	7.3	0.8	0.2	3.0	0.8
129	2021	Both	55-59 years	Low SDI	37.4	-0.4	-0.1	-0.3	0.7	2.7	68.0	8.5	0.8	0.2	3.0	0.8
130	2021	Male	60-64 years	Low SDI	43.5	0.0	-0.2	0.0	0.7	2.3	68.1	9.6	0.8	0.2	3.0	0.8
131	2021	Female	60-64 years	Low SDI	42.4	-0.2	0.5	-0.1	0.7	2.6	60.8	7.7	0.8	0.2	3.0	0.8
132	2021	Both	60-64 years	Low SDI	43.0	-0.1	0.1	0.0	0.7	2.4	65.0	8.8	0.8	0.2	3.0	0.8
133	2021	Male	65-69	Low SDI	50.3	0.3	0.3	0.3	0.8	2.1	60.7	9.3	0.8	0.2	3.0	0.8

			years													
134	2021	Female	65-69 years	Low SDI	49.3	0.1	1.0	0.3	0.7	2.4	53.7	7.4	0.8	0.2	3.0	0.8
135	2021	Both	65-69 years	Low SDI	49.8	0.2	0.6	0.3	0.7	2.2	57.8	8.4	0.8	0.2	3.0	0.8
136	2021	Male	70-74 years	Low SDI	56.8	0.6	0.7	0.6	0.8	1.8	55.2	9.2	0.8	0.2	3.0	0.8
137	2021	Female	70-74 years	Low SDI	55.6	0.4	1.2	0.6	0.7	2.0	49.7	7.6	0.8	0.2	3.0	0.8
138	2021	Both	70-74 years	Low SDI	56.3	0.5	0.9	0.6	0.8	1.9	53.0	8.5	0.8	0.2	3.0	0.8
139	2021	Male	75-79 years	Low SDI	63.6	1.0	0.8	0.9	0.9	1.5	52.0	9.7	0.8	0.2	3.0	0.8
140	2021	Female	75-79 years	Low SDI	62.8	0.8	1.2	0.9	0.8	1.6	47.3	8.2	0.8	0.2	3.0	0.8
141	2021	Both	75-79 years	Low SDI	63.3	0.9	1.0	0.9	0.9	1.5	50.0	9.1	0.8	0.2	3.0	0.8
142	2021	Male	80-84 years	Low SDI	73.0	1.6	0.7	1.4	1.0	1.1	50.0	11.7	0.8	0.2	3.0	0.8
143	2021	Female	80-84 years	Low SDI	72.7	1.5	0.9	1.4	0.9	1.1	47.3	10.8	0.8	0.2	3.0	0.8
144	2021	Both	80-84 years	Low SDI	72.9	1.6	0.8	1.4	1.0	1.1	48.7	11.3	0.8	0.2	3.0	0.8
145	2021	Male	85-89 years	Low SDI	81.6	2.2	0.5	1.8	1.1	0.8	48.7	14.1	0.8	0.2	3.0	0.8
146	2021	Female	85-89 years	Low SDI	81.8	2.2	0.6	1.8	1.1	0.8	47.4	13.7	0.8	0.2	3.0	0.8
147	2021	Both	85-89 years	Low SDI	81.7	2.2	0.5	1.8	1.1	0.8	48.0	13.9	0.8	0.2	3.0	0.8
148	2021	Male	90-94 years	Low SDI	88.8	2.8	-0.2	2.2	1.2	0.6	52.6	17.4	0.8	0.2	3.0	0.8
149	2021	Female	90-94 years	Low SDI	89.6	2.8	-0.1	2.2	1.3	0.6	51.7	17.4	0.8	0.2	3.0	0.8
150	2021	Both	90-94 years	Low SDI	89.3	2.8	-0.2	2.2	1.3	0.6	52.1	17.4	0.8	0.2	3.0	0.8
151	2021	Male	95+ years	Low SDI	98.0	3.8	-1.9	2.6	1.5	0.5	64.4	25.1	0.8	0.2	3.0	0.8
152	2021	Female	95+ years	Low SDI	100.0	3.9	-1.7	2.7	1.5	0.5	61.9	25.1	0.8	0.2	3.0	0.8
153	2021	Both	95+ years	Low SDI	99.4	3.9	-1.7	2.7	1.5	0.5	62.7	25.1	0.8	0.2	3.0	0.8
154	2021	Male	15-19 years	Low-middle SDI	0.0	-1.8	-2.6	-2.0	0.5	4.4	94.1	8.5	0.7	0.3	2.9	1.0
155	2021	Female	15-19 years	Low-middle SDI	8.1	-1.9	-0.9	-1.6	0.4	4.8	74.7	6.4	0.7	0.3	2.9	1.0
156	2021	Both	15-19 years	Low-middle SDI	5.3	-1.9	-1.5	-1.8	0.5	4.6	81.4	7.1	0.7	0.3	2.9	1.0

157	2021	Male	20-24 years	Low-middle SDI	4.1	-1.7	-2.2	-1.8	0.5	4.4	89.5	8.2	0.7	0.3	2.9	1.0
158	2021	Female	20-24 years	Low-middle SDI	9.6	-1.8	-0.8	-1.6	0.4	4.7	73.8	6.3	0.7	0.3	2.9	1.0
159	2021	Both	20-24 years	Low-middle SDI	7.5	-1.8	-1.3	-1.7	0.5	4.6	79.6	7.0	0.7	0.3	2.9	1.0
160	2021	Male	25-29 years	Low-middle SDI	10.2	-1.8	-0.8	-1.5	0.5	4.9	73.0	6.2	0.7	0.3	2.9	1.0
161	2021	Female	25-29 years	Low-middle SDI	15.8	-1.9	0.4	-1.3	0.4	5.2	58.6	4.9	0.7	0.3	2.9	1.0
162	2021	Both	25-29 years	Low-middle SDI	13.3	-1.8	-0.1	-1.4	0.4	5.1	65.1	5.5	0.7	0.3	2.9	1.0
163	2021	Male	30-34 years	Low-middle SDI	16.0	-1.7	-0.1	-1.3	0.5	4.9	64.4	5.6	0.7	0.3	2.9	1.0
164	2021	Female	30-34 years	Low-middle SDI	19.6	-1.8	1.0	-1.1	0.4	5.3	51.9	4.3	0.7	0.3	2.9	1.0
165	2021	Both	30-34 years	Low-middle SDI	17.7	-1.7	0.4	-1.2	0.4	5.1	58.3	4.9	0.7	0.3	2.9	1.0
166	2021	Male	35-39 years	Low-middle SDI	23.9	-1.4	0.5	-0.9	0.4	4.4	57.7	5.5	0.7	0.3	2.9	1.0
167	2021	Female	35-39 years	Low-middle SDI	25.5	-1.6	1.4	-0.8	0.4	4.9	46.9	4.0	0.7	0.3	2.9	1.0
168	2021	Both	35-39 years	Low-middle SDI	24.5	-1.5	0.9	-0.9	0.4	4.6	53.3	4.8	0.7	0.3	2.9	1.0
169	2021	Male	40-44 years	Low-middle SDI	24.6	-0.9	-0.7	-0.9	0.6	3.5	71.4	7.7	0.7	0.3	2.9	1.0
170	2021	Female	40-44 years	Low-middle SDI	27.3	-1.2	0.6	-0.8	0.5	4.1	56.1	5.4	0.7	0.3	2.9	1.0
171	2021	Both	40-44 years	Low-middle SDI	25.7	-1.1	-0.2	-0.8	0.5	3.7	65.2	6.7	0.7	0.3	2.9	1.0
172	2021	Male	45-49 years	Low-middle SDI	28.1	-0.6	-0.9	-0.7	0.6	3.1	73.2	8.5	0.7	0.3	2.9	1.0
173	2021	Female	45-49 years	Low-middle SDI	31.1	-0.9	0.4	-0.6	0.5	3.7	57.9	6.1	0.7	0.3	2.9	1.0
174	2021	Both	45-49 years	Low-middle SDI	29.4	-0.8	-0.4	-0.7	0.6	3.4	66.8	7.4	0.7	0.3	2.9	1.0
175	2021	Male	50-54 years	Low-middle SDI	33.2	-0.4	-0.7	-0.5	0.6	3.0	69.9	8.5	0.7	0.3	2.9	1.0
176	2021	Female	50-54 years	Low-middle SDI	33.7	-0.7	0.3	-0.5	0.6	3.4	59.5	6.4	0.7	0.3	2.9	1.0
177	2021	Both	50-54 years	Low-middle SDI	33.3	-0.5	-0.3	-0.5	0.6	3.1	65.9	7.7	0.7	0.3	2.9	1.0
178	2021	Male	55-59 years	Low-middle SDI	37.2	-0.3	-0.4	-0.3	0.7	2.8	66.8	8.2	0.7	0.3	2.9	1.0

179	2021	Female	55-59 years	Low-middle SDI	38.7	-0.5	0.6	-0.2	0.6	3.2	55.7	6.3	0.7	0.3	2.9	1.0
180	2021	Both	55-59 years	Low-middle SDI	37.8	-0.4	0.0	-0.3	0.6	2.9	62.1	7.4	0.7	0.3	2.9	1.0
181	2021	Male	60-64 years	Low-middle SDI	43.4	0.0	-0.2	0.0	0.7	2.5	63.3	8.4	0.7	0.3	2.9	1.0
182	2021	Female	60-64 years	Low-middle SDI	43.3	-0.2	0.6	0.0	0.6	2.8	55.6	6.7	0.7	0.3	2.9	1.0
183	2021	Both	60-64 years	Low-middle SDI	43.3	-0.1	0.1	0.0	0.7	2.6	60.1	7.7	0.7	0.3	2.9	1.0
184	2021	Male	65-69 years	Low-middle SDI	49.9	0.3	0.3	0.3	0.7	2.2	57.5	8.1	0.7	0.3	2.9	1.0
185	2021	Female	65-69 years	Low-middle SDI	49.5	0.0	1.0	0.2	0.7	2.6	50.3	6.4	0.7	0.3	2.9	1.0
186	2021	Both	65-69 years	Low-middle SDI	49.7	0.1	0.6	0.3	0.7	2.4	54.5	7.4	0.7	0.3	2.9	1.0
187	2021	Male	70-74 years	Low-middle SDI	56.9	0.5	0.7	0.6	0.8	2.0	52.3	8.0	0.7	0.3	2.9	1.0
188	2021	Female	70-74 years	Low-middle SDI	56.2	0.3	1.3	0.5	0.7	2.2	46.5	6.5	0.7	0.3	2.9	1.0
189	2021	Both	70-74 years	Low-middle SDI	56.6	0.4	0.9	0.6	0.7	2.1	49.8	7.3	0.7	0.3	2.9	1.0
190	2021	Male	75-79 years	Low-middle SDI	64.5	0.9	0.9	0.9	0.8	1.6	48.2	8.3	0.7	0.3	2.9	1.0
191	2021	Female	75-79 years	Low-middle SDI	63.8	0.7	1.4	0.9	0.8	1.8	43.7	7.0	0.7	0.3	2.9	1.0
192	2021	Both	75-79 years	Low-middle SDI	64.1	0.8	1.2	0.9	0.8	1.7	46.2	7.7	0.7	0.3	2.9	1.0
193	2021	Male	80-84 years	Low-middle SDI	73.4	1.5	0.9	1.3	0.9	1.2	46.8	10.0	0.7	0.3	2.9	1.0
194	2021	Female	80-84 years	Low-middle SDI	72.3	1.3	1.1	1.3	0.9	1.3	45.4	9.1	0.7	0.3	2.9	1.0
195	2021	Both	80-84 years	Low-middle SDI	72.8	1.4	1.0	1.3	0.9	1.2	46.1	9.5	0.7	0.3	2.9	1.0
196	2021	Male	85-89 years	Low-middle SDI	82.1	2.1	0.7	1.7	1.0	0.8	46.1	12.3	0.7	0.3	2.9	1.0
197	2021	Female	85-89 years	Low-middle SDI	81.0	2.0	0.7	1.7	1.0	0.9	46.5	11.9	0.7	0.3	2.9	1.0
198	2021	Both	85-89 years	Low-middle SDI	81.5	2.0	0.7	1.7	1.0	0.9	46.3	12.1	0.7	0.3	2.9	1.0
199	2021	Male	90-94 years	Low-middle SDI	89.1	2.8	-0.1	2.0	1.2	0.7	51.0	15.9	0.7	0.3	2.9	1.0
200	2021	Female	90-94 years	Low-middle SDI	88.7	2.8	-0.2	2.0	1.2	0.7	51.9	15.9	0.7	0.3	2.9	1.0

201	2021	Both	90-94 years	Low-middle SDI	88.9	2.8	-0.1	2.0	1.2	0.7	51.5	15.9	0.7	0.3	2.9	1.0
202	2021	Male	95+ years	Low-middle SDI	100.0	4.0	-1.8	2.5	1.5	0.5	62.0	24.4	0.7	0.3	2.9	1.0
203	2021	Female	95+ years	Low-middle SDI	97.9	4.0	-2.0	2.4	1.5	0.5	64.5	24.2	0.7	0.3	2.9	1.0
204	2021	Both	95+ years	Low-middle SDI	98.8	4.0	-1.9	2.5	1.5	0.5	63.5	24.2	0.7	0.3	2.9	1.0
205	2021	Male	15-19 years	Middle SDI	96.8	0.5	2.7	1.1	0.4	5.2	64.9	5.4	0.7	0.3	2.7	1.1
206	2021	Female	15-19 years	Middle SDI	100.0	1.5	0.8	1.3	0.3	5.8	44.3	3.5	0.7	0.3	2.7	1.1
207	2021	Both	15-19 years	Middle SDI	98.4	1.0	1.7	1.2	0.3	5.5	54.4	4.4	0.7	0.3	2.7	1.1
208	2021	Male	20-24 years	Middle SDI	95.1	0.5	2.4	1.1	0.4	5.1	62.1	5.3	0.7	0.3	2.7	1.1
209	2021	Female	20-24 years	Middle SDI	98.7	1.4	0.8	1.2	0.3	5.7	44.9	3.5	0.7	0.3	2.7	1.1
210	2021	Both	20-24 years	Middle SDI	96.8	0.9	1.6	1.1	0.3	5.4	53.9	4.4	0.7	0.3	2.7	1.1
211	2021	Male	25-29 years	Middle SDI	96.6	1.0	1.4	1.1	0.4	5.5	51.8	4.2	0.7	0.3	2.7	1.1
212	2021	Female	25-29 years	Middle SDI	99.6	1.8	0.1	1.3	0.3	6.0	36.6	2.8	0.7	0.3	2.7	1.1
213	2021	Both	25-29 years	Middle SDI	97.8	1.3	0.9	1.2	0.3	5.7	45.5	3.6	0.7	0.3	2.7	1.1
214	2021	Male	30-34 years	Middle SDI	95.9	1.3	0.7	1.1	0.3	5.6	44.1	3.6	0.7	0.3	2.7	1.1
215	2021	Female	30-34 years	Middle SDI	99.3	2.0	-0.4	1.3	0.3	6.1	31.3	2.5	0.7	0.3	2.7	1.1
216	2021	Both	30-34 years	Middle SDI	97.1	1.5	0.3	1.2	0.3	5.8	39.3	3.2	0.7	0.3	2.7	1.1
217	2021	Male	35-39 years	Middle SDI	92.2	1.3	0.1	0.9	0.3	5.2	39.1	3.4	0.7	0.3	2.7	1.1
218	2021	Female	35-39 years	Middle SDI	96.9	2.0	-0.9	1.2	0.2	5.8	27.7	2.2	0.7	0.3	2.7	1.1
219	2021	Both	35-39 years	Middle SDI	93.6	1.5	-0.2	1.0	0.3	5.4	35.6	3.0	0.7	0.3	2.7	1.1
220	2021	Male	40-44 years	Middle SDI	85.6	0.5	1.0	0.7	0.4	4.4	49.3	4.7	0.7	0.3	2.7	1.1
221	2021	Female	40-44 years	Middle SDI	91.6	1.4	-0.3	0.9	0.3	5.2	35.2	3.0	0.7	0.3	2.7	1.1
222	2021	Both	40-44 years	Middle SDI	87.4	0.8	0.6	0.7	0.4	4.7	45.1	4.2	0.7	0.3	2.7	1.1

223	2021	Male	45-49 years	Middle SDI	81.1	0.3	0.9	0.5	0.5	4.0	49.9	5.1	0.7	0.3	2.7	1.1
224	2021	Female	45-49 years	Middle SDI	87.1	1.1	-0.2	0.7	0.4	4.8	37.0	3.4	0.7	0.3	2.7	1.1
225	2021	Both	45-49 years	Middle SDI	82.9	0.5	0.6	0.5	0.4	4.3	45.9	4.6	0.7	0.3	2.7	1.1
226	2021	Male	50-54 years	Middle SDI	77.9	0.2	0.7	0.3	0.5	3.8	48.1	5.1	0.7	0.3	2.7	1.1
227	2021	Female	50-54 years	Middle SDI	84.2	1.0	-0.3	0.6	0.4	4.5	36.9	3.5	0.7	0.3	2.7	1.1
228	2021	Both	50-54 years	Middle SDI	79.6	0.4	0.4	0.4	0.5	4.0	45.0	4.6	0.7	0.3	2.7	1.1
229	2021	Male	55-59 years	Middle SDI	75.1	0.1	0.4	0.2	0.5	3.6	45.4	5.0	0.7	0.3	2.7	1.1
230	2021	Female	55-59 years	Middle SDI	81.1	0.9	-0.5	0.5	0.4	4.3	35.1	3.5	0.7	0.3	2.7	1.1
231	2021	Both	55-59 years	Middle SDI	76.9	0.3	0.1	0.3	0.5	3.8	42.3	4.5	0.7	0.3	2.7	1.1
232	2021	Male	60-64 years	Middle SDI	70.2	-0.1	0.2	0.0	0.6	3.2	44.1	5.2	0.7	0.3	2.7	1.1
233	2021	Female	60-64 years	Middle SDI	76.3	0.6	-0.5	0.2	0.5	3.8	35.7	3.7	0.7	0.3	2.7	1.1
234	2021	Both	60-64 years	Middle SDI	72.1	0.1	0.0	0.1	0.5	3.4	41.5	4.7	0.7	0.3	2.7	1.1
235	2021	Male	65-69 years	Middle SDI	67.3	0.0	-0.4	-0.1	0.6	3.0	38.3	4.7	0.7	0.3	2.7	1.1
236	2021	Female	65-69 years	Middle SDI	73.6	0.6	-1.0	0.1	0.5	3.7	31.1	3.4	0.7	0.3	2.7	1.1
237	2021	Both	65-69 years	Middle SDI	69.3	0.2	-0.6	-0.1	0.5	3.2	36.0	4.2	0.7	0.3	2.7	1.1
238	2021	Male	70-74 years	Middle SDI	63.7	0.0	-0.9	-0.3	0.6	2.7	33.9	4.4	0.7	0.3	2.7	1.1
239	2021	Female	70-74 years	Middle SDI	69.4	0.5	-1.4	-0.1	0.5	3.3	28.4	3.3	0.7	0.3	2.7	1.1
240	2021	Both	70-74 years	Middle SDI	65.6	0.1	-1.1	-0.2	0.6	2.9	32.1	4.0	0.7	0.3	2.7	1.1
241	2021	Male	75-79 years	Middle SDI	57.6	-0.3	-1.2	-0.6	0.6	2.2	32.1	4.7	0.7	0.3	2.7	1.1
242	2021	Female	75-79 years	Middle SDI	62.9	0.2	-1.6	-0.3	0.6	2.8	27.1	3.5	0.7	0.3	2.7	1.1
243	2021	Both	75-79 years	Middle SDI	59.5	-0.1	-1.4	-0.5	0.6	2.4	30.2	4.2	0.7	0.3	2.7	1.1
244	2021	Male	80-84 years	Middle SDI	48.3	-0.9	-1.3	-1.0	0.7	1.6	32.9	6.0	0.7	0.3	2.7	1.1

245	2021	Female	80-84 years	Middle SDI	51.7	-0.5	-1.5	-0.8	0.7	1.9	29.7	4.8	0.7	0.3	2.7	1.1
246	2021	Both	80-84 years	Middle SDI	49.8	-0.7	-1.4	-0.9	0.7	1.7	31.5	5.4	0.7	0.3	2.7	1.1
247	2021	Male	85-89 years	Middle SDI	38.3	-1.5	-1.2	-1.4	0.8	1.0	34.2	8.2	0.7	0.3	2.7	1.1
248	2021	Female	85-89 years	Middle SDI	41.2	-1.2	-1.5	-1.3	0.8	1.3	31.4	6.6	0.7	0.3	2.7	1.1
249	2021	Both	85-89 years	Middle SDI	39.7	-1.4	-1.3	-1.4	0.8	1.1	32.9	7.4	0.7	0.3	2.7	1.1
250	2021	Male	90-94 years	Middle SDI	24.4	-2.7	-0.3	-2.0	1.0	0.7	42.3	12.7	0.7	0.3	2.7	1.1
251	2021	Female	90-94 years	Middle SDI	27.2	-2.4	-0.6	-1.9	1.0	0.8	40.0	10.9	0.7	0.3	2.7	1.1
252	2021	Both	90-94 years	Middle SDI	26.0	-2.6	-0.5	-2.0	1.0	0.8	41.0	11.7	0.7	0.3	2.7	1.1
253	2021	Male	95+ years	Middle SDI	0.0	-5.0	1.4	-3.1	1.4	0.5	57.5	22.7	0.7	0.3	2.7	1.1
254	2021	Female	95+ years	Middle SDI	1.1	-4.9	1.3	-3.1	1.4	0.5	56.8	22.2	0.7	0.3	2.7	1.1
255	2021	Both	95+ years	Middle SDI	0.7	-4.9	1.4	-3.1	1.4	0.5	57.0	22.4	0.7	0.3	2.7	1.1
256	2021	Male	15-19 years	High-middle SDI	85.4	0.1	2.2	0.6	0.3	5.6	46.0	3.7	0.7	0.3	2.8	1.0
257	2021	Female	15-19 years	High-middle SDI	96.0	1.3	0.9	1.2	0.2	6.3	29.5	2.3	0.7	0.3	2.8	1.0
258	2021	Both	15-19 years	High-middle SDI	91.3	0.8	1.5	1.0	0.2	6.0	36.7	2.9	0.7	0.3	2.8	1.0
259	2021	Male	20-24 years	High-middle SDI	86.1	0.2	1.9	0.7	0.3	5.6	42.4	3.5	0.7	0.3	2.8	1.0
260	2021	Female	20-24 years	High-middle SDI	95.7	1.3	0.8	1.2	0.2	6.3	29.0	2.2	0.7	0.3	2.8	1.0
261	2021	Both	20-24 years	High-middle SDI	90.6	0.7	1.4	0.9	0.2	5.9	36.1	2.9	0.7	0.3	2.8	1.0
262	2021	Male	25-29 years	High-middle SDI	89.7	0.7	1.3	0.9	0.3	5.9	35.7	2.8	0.7	0.3	2.8	1.0
263	2021	Female	25-29 years	High-middle SDI	98.7	1.7	0.3	1.4	0.2	6.5	23.3	1.8	0.7	0.3	2.8	1.0
264	2021	Both	25-29 years	High-middle SDI	93.5	1.1	0.9	1.1	0.2	6.1	30.3	2.4	0.7	0.3	2.8	1.0
265	2021	Male	30-34 years	High-middle SDI	91.0	1.0	0.8	0.9	0.2	5.8	30.2	2.5	0.7	0.3	2.8	1.0
266	2021	Female	30-34 years	High-middle SDI	100.0	1.9	0.0	1.4	0.2	6.6	19.9	1.5	0.7	0.3	2.8	1.0
267	2021	Both	30-34 years	High-middle SDI	94.4	1.3	0.5	1.1	0.2	6.1	26.2	2.1	0.7	0.3	2.8	1.0
268	2021	Male	35-39	High-middle	88.9	1.0	0.3	0.8	0.2	5.2	26.6	2.4	0.7	0.3	2.8	1.0

			years	SDI											
269	2021	Female	35-39 years	High-middle SDI	99.4	2.0	-0.2	1.4	0.2	6.4	17.8	1.4	0.7	0.3	2.8	1.0
270	2021	Both	35-39 years	High-middle SDI	92.3	1.3	0.1	1.0	0.2	5.6	23.7	2.0	0.7	0.3	2.8	1.0
271	2021	Male	40-44 years	High-middle SDI	79.4	0.1	0.9	0.3	0.3	4.4	35.4	3.4	0.7	0.3	2.8	1.0
272	2021	Female	40-44 years	High-middle SDI	93.8	1.4	0.1	1.1	0.2	6.0	23.0	1.9	0.7	0.3	2.8	1.0
273	2021	Both	40-44 years	High-middle SDI	83.5	0.5	0.6	0.5	0.3	4.8	31.7	2.9	0.7	0.3	2.8	1.0
274	2021	Male	45-49 years	High-middle SDI	75.8	-0.2	0.9	0.1	0.4	4.2	36.8	3.7	0.7	0.3	2.8	1.0
275	2021	Female	45-49 years	High-middle SDI	90.8	1.2	0.1	0.9	0.3	5.7	23.6	2.1	0.7	0.3	2.8	1.0
276	2021	Both	45-49 years	High-middle SDI	79.9	0.2	0.6	0.3	0.3	4.5	33.0	3.2	0.7	0.3	2.8	1.0
277	2021	Male	50-54 years	High-middle SDI	74.6	-0.2	0.7	0.0	0.4	4.1	35.5	3.7	0.7	0.3	2.8	1.0
278	2021	Female	50-54 years	High-middle SDI	88.4	1.1	0.0	0.8	0.3	5.4	24.0	2.1	0.7	0.3	2.8	1.0
279	2021	Both	50-54 years	High-middle SDI	78.0	0.1	0.5	0.2	0.4	4.4	32.6	3.2	0.7	0.3	2.8	1.0
280	2021	Male	55-59 years	High-middle SDI	73.9	-0.1	0.4	0.0	0.4	3.9	33.1	3.5	0.7	0.3	2.8	1.0
281	2021	Female	55-59 years	High-middle SDI	86.9	1.0	-0.2	0.7	0.3	5.3	22.7	2.1	0.7	0.3	2.8	1.0
282	2021	Both	55-59 years	High-middle SDI	77.1	0.2	0.2	0.2	0.4	4.2	30.3	3.0	0.7	0.3	2.8	1.0
283	2021	Male	60-64 years	High-middle SDI	70.6	-0.3	0.2	-0.2	0.4	3.6	31.9	3.6	0.7	0.3	2.8	1.0
284	2021	Female	60-64 years	High-middle SDI	83.0	0.8	-0.3	0.5	0.3	4.8	22.9	2.2	0.7	0.3	2.8	1.0
285	2021	Both	60-64 years	High-middle SDI	73.8	0.0	0.0	0.0	0.4	3.9	29.5	3.1	0.7	0.3	2.8	1.0
286	2021	Male	65-69 years	High-middle SDI	70.7	-0.1	-0.3	-0.2	0.4	3.5	27.8	3.2	0.7	0.3	2.8	1.0
287	2021	Female	65-69 years	High-middle SDI	82.4	0.9	-0.7	0.5	0.4	4.7	19.7	2.0	0.7	0.3	2.8	1.0
288	2021	Both	65-69 years	High-middle SDI	74.0	0.2	-0.4	0.0	0.4	3.8	25.4	2.8	0.7	0.3	2.8	1.0
289	2021	Male	70-74 years	High-middle SDI	70.6	0.0	-0.7	-0.2	0.4	3.4	23.6	2.8	0.7	0.3	2.8	1.0
290	2021	Female	70-74	High-middle	79.6	0.8	-1.0	0.3	0.4	4.3	17.9	1.9	0.7	0.3	2.8	1.0

			years	SDI												
291	2021	Both	70-74 years	High-middle SDI	73.5	0.3	-0.8	0.0	0.4	3.7	21.7	2.5	0.7	0.3	2.8	1.0
292	2021	Male	75-79 years	High-middle SDI	69.3	0.1	-1.1	-0.2	0.5	3.2	20.4	2.5	0.7	0.3	2.8	1.0
293	2021	Female	75-79 years	High-middle SDI	76.1	0.6	-1.3	0.1	0.4	4.0	16.3	1.9	0.7	0.3	2.8	1.0
294	2021	Both	75-79 years	High-middle SDI	71.8	0.3	-1.2	-0.1	0.5	3.4	18.8	2.2	0.7	0.3	2.8	1.0
295	2021	Male	80-84 years	High-middle SDI	61.2	-0.4	-1.3	-0.7	0.6	2.4	21.2	3.1	0.7	0.3	2.8	1.0
296	2021	Female	80-84 years	High-middle SDI	66.7	0.0	-1.5	-0.4	0.5	2.9	17.5	2.4	0.7	0.3	2.8	1.0
297	2021	Both	80-84 years	High-middle SDI	63.7	-0.2	-1.4	-0.5	0.5	2.6	19.4	2.7	0.7	0.3	2.8	1.0
298	2021	Male	85-89 years	High-middle SDI	54.1	-0.9	-1.5	-1.1	0.6	1.7	21.7	3.8	0.7	0.3	2.8	1.0
299	2021	Female	85-89 years	High-middle SDI	59.4	-0.4	-1.7	-0.8	0.6	2.2	18.1	2.8	0.7	0.3	2.8	1.0
300	2021	Both	85-89 years	High-middle SDI	56.7	-0.7	-1.6	-0.9	0.6	2.0	19.8	3.3	0.7	0.3	2.8	1.0
301	2021	Male	90-94 years	High-middle SDI	40.0	-2.1	-1.1	-1.8	0.8	1.2	28.3	6.3	0.7	0.3	2.8	1.0
302	2021	Female	90-94 years	High-middle SDI	43.0	-1.8	-1.4	-1.7	0.8	1.3	25.1	5.4	0.7	0.3	2.8	1.0
303	2021	Both	90-94 years	High-middle SDI	41.8	-1.9	-1.2	-1.7	0.8	1.2	26.4	5.7	0.7	0.3	2.8	1.0
304	2021	Male	95+ years	High-middle SDI	0.0	-5.8	1.1	-4.0	1.2	0.5	49.2	19.6	0.7	0.3	2.8	1.0
305	2021	Female	95+ years	High-middle SDI	0.8	-5.7	0.9	-3.9	1.2	0.5	47.3	19.4	0.7	0.3	2.8	1.0
306	2021	Both	95+ years	High-middle SDI	0.5	-5.7	1.0	-4.0	1.2	0.5	47.9	19.5	0.7	0.3	2.8	1.0

Table S4. Univariate analysis of the Quality of Care Index and related indicators for lip and oral cavity cancer among individuals aged 15 years and older, 1990–2021: values stratified by sex and Socio-demographic Index region.

	Sexy	location	year	Deaths	DALY	YLD	YLL	Prevalence	Incidence	YLR	DPR	MIR	PIR	PCAscore	QCI
1	Both	Middle SDI	1991	2.3	61.2	1.0	60.2	8.0	3.2	57.9	7.7	0.7	2.5	-1.1	23.9
2	Both	High-middle SDI	1990	2.0	55.7	1.3	54.4	11.5	3.6	41.3	4.9	0.5	3.2	0.5	52.0
3	Both	High-middle SDI	1992	2.0	57.1	1.4	55.8	12.0	3.8	40.6	4.7	0.5	3.2	0.6	53.2
4	Both	High SDI	1992	1.9	53.2	2.4	50.9	25.2	5.4	21.5	2.1	0.3	4.6	2.6	89.2
5	Both	High SDI	1990	1.9	54.1	2.4	51.8	25.1	5.4	21.9	2.2	0.4	4.6	2.6	88.5
6	Both	Low-middle SDI	1992	4.3	120.2	1.7	118.5	11.9	5.5	70.8	10.1	0.8	2.2	-2.1	5.8
7	Both	Low-middle SDI	1990	4.3	119.9	1.7	118.2	11.7	5.4	71.4	10.2	0.8	2.2	-2.2	4.7
8	Both	Low SDI	1992	3.2	89.0	1.2	87.8	8.2	4.0	72.6	10.9	0.8	2.1	-2.4	0.6
9	Both	Middle SDI	1992	2.3	61.6	1.1	60.5	8.1	3.2	57.3	7.6	0.7	2.6	-1.0	24.8
10	Both	Middle SDI	1990	2.3	61.0	1.0	60.0	7.8	3.1	58.3	7.8	0.7	2.5	-1.2	22.9
11	Both	Low SDI	1991	3.2	88.4	1.2	87.2	8.1	3.9	72.8	10.9	0.8	2.1	-2.4	0.4
12	Both	Low SDI	1990	3.2	87.8	1.2	86.6	8.0	3.9	72.9	11.0	0.8	2.0	-2.5	0.2
13	Both	High-middle SDI	1991	2.0	56.1	1.3	54.7	11.7	3.7	40.8	4.8	0.5	3.2	0.6	52.7
14	Both	High-middle SDI	1994	2.1	58.9	1.4	57.5	12.4	3.9	40.4	4.7	0.5	3.2	0.6	53.4
15	Both	High SDI	1991	1.9	53.9	2.4	51.5	25.2	5.4	21.7	2.1	0.4	4.6	2.6	88.8
16	Both	Low-middle SDI	1991	4.3	119.5	1.7	117.9	11.8	5.4	71.2	10.2	0.8	2.2	-2.2	5.3
17	Both	High SDI	1994	1.9	52.4	2.4	50.0	25.5	5.5	20.9	2.1	0.3	4.7	2.7	90.1
18	Both	Low SDI	1994	3.4	91.9	1.2	90.6	8.4	4.1	72.6	11.0	0.8	2.0	-2.5	0.0
19	Both	High-middle SDI	1997	2.0	54.9	1.4	53.5	12.3	3.7	38.8	4.5	0.5	3.3	0.7	56.0
20	Both	Low-middle SDI	1994	4.4	122.1	1.7	120.4	12.2	5.6	70.5	10.0	0.8	2.2	-2.1	6.0
21	Both	High SDI	1997	1.8	51.4	2.5	49.0	26.3	5.7	19.9	2.0	0.3	4.6	2.8	91.4
22	Both	Middle SDI	1994	2.3	62.6	1.1	61.5	8.5	3.3	56.3	7.4	0.7	2.6	-1.0	26.1
23	Both	Low-middle SDI	1997	4.5	123.9	1.7	122.2	12.5	5.6	70.6	9.9	0.8	2.2	-2.1	6.2
24	Both	Low SDI	1997	3.4	92.7	1.3	91.4	8.5	4.1	73.0	10.9	0.8	2.1	-2.5	0.1
25	Both	Middle SDI	1997	2.3	62.1	1.1	61.0	8.8	3.3	55.0	7.1	0.7	2.6	-0.8	28.3
26	Both	Low-middle SDI	1995	4.5	123.3	1.7	121.6	12.4	5.6	70.5	10.0	0.8	2.2	-2.1	5.9
27	Both	Low-middle SDI	1998	4.5	124.3	1.7	122.5	12.6	5.7	70.4	9.9	0.8	2.2	-2.1	6.5
28	Both	Low SDI	1995	3.4	92.5	1.3	91.3	8.5	4.2	72.6	10.9	0.8	2.0	-2.5	0.1
29	Both	High-middle SDI	1995	2.0	57.8	1.4	56.4	12.4	3.9	39.8	4.7	0.5	3.2	0.6	54.2
30	Both	Low SDI	1998	3.4	93.1	1.3	91.9	8.6	4.1	73.1	10.9	0.8	2.1	-2.5	0.2
31	Both	High SDI	1995	1.9	52.6	2.4	50.2	26.0	5.6	20.5	2.0	0.3	4.6	2.7	90.4
32	Both	High SDI	1999	1.8	50.3	2.4	47.9	26.2	5.6	19.6	1.9	0.3	4.7	2.8	92.3
33	Both	High-middle SDI	1998	1.9	53.6	1.4	52.3	12.1	3.7	38.5	4.4	0.5	3.3	0.8	56.4
34	Both	Low-middle SDI	1999	4.5	124.0	1.7	122.2	12.6	5.6	70.3	9.8	0.8	2.2	-2.1	7.0
35	Both	Middle SDI	1995	2.4	62.8	1.1	61.7	8.6	3.3	56.0	7.3	0.7	2.6	-0.9	26.7
36	Both	High SDI	1998	1.8	51.1	2.5	48.6	26.3	5.7	19.8	1.9	0.3	4.7	2.8	91.7
37	Both	Low SDI	1999	3.4	92.6	1.3	91.3	8.5	4.1	72.9	10.9	0.8	2.1	-2.4	0.5
38	Both	Middle SDI	1999	2.3	61.4	1.1	60.3	8.9	3.3	54.0	6.9	0.7	2.7	-0.7	30.1
39	Both	Low SDI	2001	3.4	92.2	1.2	90.9	8.5	4.1	72.8	10.8	0.8	2.1	-2.4	0.7
40	Both	Middle SDI	1998	2.3	61.8	1.1	60.7	8.8	3.3	54.5	7.0	0.7	2.7	-0.8	29.2
41	Both	High-middle SDI	2000	1.9	54.0	1.4	52.6	12.4	3.7	38.2	4.4	0.5	3.4	0.8	57.2

42	Both	Middle SDI	2001	2.3	60.9	1.1	59.8	9.0	3.3	53.1	6.7	0.7	2.7	-0.7	31.6
43	Both	Low-middle SDI	2000	4.4	124.0	1.7	122.2	12.7	5.7	70.1	9.8	0.8	2.2	-2.0	7.3
44	Both	Low SDI	2000	3.4	92.6	1.3	91.3	8.5	4.1	72.9	10.8	0.8	2.1	-2.4	0.5
45	Both	High SDI	2000	1.8	50.1	2.5	47.7	26.8	5.6	19.4	1.9	0.3	4.8	2.9	93.3
46	Both	High-middle SDI	1999	1.9	54.2	1.4	52.8	12.3	3.7	38.5	4.4	0.5	3.3	0.8	56.6
47	Both	High-middle SDI	2003	1.9	51.7	1.4	50.4	12.5	3.6	36.9	4.2	0.5	3.5	0.9	59.6
48	Both	Middle SDI	2000	2.3	61.1	1.1	60.0	9.0	3.3	53.5	6.8	0.7	2.7	-0.7	30.9
49	Both	High SDI	2003	1.7	48.6	2.5	46.1	27.3	5.5	18.6	1.8	0.3	4.9	3.0	95.4
50	Both	Low-middle SDI	2003	4.5	123.8	1.8	122.0	12.8	5.7	69.3	9.7	0.8	2.2	-2.0	8.3
51	Both	Low SDI	2003	3.3	91.5	1.2	90.3	8.5	4.1	72.2	10.7	0.8	2.1	-2.4	1.3
52	Both	High-middle SDI	2001	1.9	53.3	1.4	51.9	12.4	3.7	37.8	4.3	0.5	3.4	0.9	58.0
53	Both	High SDI	2001	1.8	49.9	2.5	47.4	27.3	5.6	19.0	1.8	0.3	4.9	2.9	94.3
54	Both	Middle SDI	2003	2.3	60.3	1.1	59.2	9.2	3.3	52.1	6.6	0.7	2.8	-0.6	33.1
55	Both	Low SDI	2005	3.3	89.7	1.2	88.5	8.5	4.1	71.8	10.6	0.8	2.1	-2.3	2.3
56	Both	Low-middle SDI	2001	4.5	123.9	1.7	122.2	12.7	5.7	69.9	9.8	0.8	2.2	-2.0	7.6
57	Both	Low-middle SDI	2005	4.5	123.3	1.8	121.5	13.0	5.7	68.5	9.5	0.8	2.3	-1.9	9.7
58	Both	High SDI	2005	1.7	46.6	2.5	44.1	27.3	5.5	17.9	1.7	0.3	5.0	3.1	96.6
59	Both	High-middle SDI	2004	1.8	50.9	1.4	49.5	12.6	3.6	36.1	4.0	0.5	3.5	1.0	60.8
60	Both	High SDI	2004	1.7	47.4	2.5	44.9	27.1	5.5	18.2	1.7	0.3	4.9	3.0	96.0
61	Both	Low-middle SDI	2004	4.4	122.8	1.8	121.1	12.8	5.7	69.0	9.6	0.8	2.3	-2.0	8.9
62	Both	Middle SDI	2005	2.2	59.8	1.2	58.7	9.5	3.4	50.7	6.3	0.7	2.8	-0.4	35.4
63	Both	Middle SDI	2004	2.3	59.9	1.1	58.8	9.3	3.3	51.4	6.4	0.7	2.8	-0.5	34.1
64	Both	Low SDI	2004	3.3	90.7	1.2	89.5	8.5	4.1	72.1	10.6	0.8	2.1	-2.4	1.8
65	Both	Low SDI	2007	3.2	87.8	1.2	86.5	8.5	4.0	70.8	10.4	0.8	2.1	-2.3	3.4
66	Both	High-middle SDI	2006	1.8	49.0	1.4	47.6	13.0	3.7	34.0	3.8	0.5	3.5	1.2	64.0
67	Both	High SDI	2006	1.7	46.3	2.5	43.8	27.6	5.6	17.5	1.7	0.3	5.0	3.1	97.0
68	Both	Low-middle SDI	2006	4.4	122.6	1.8	120.8	13.1	5.7	68.0	9.3	0.8	2.3	-1.9	10.5
69	Both	Middle SDI	2007	2.2	59.4	1.2	58.2	9.9	3.4	49.0	6.0	0.7	2.9	-0.3	38.3
70	Both	High-middle SDI	2005	1.8	50.8	1.4	49.4	12.9	3.7	35.2	3.9	0.5	3.5	1.1	62.1
71	Both	Low SDI	2006	3.3	88.5	1.2	87.3	8.5	4.0	71.3	10.5	0.8	2.1	-2.3	2.8
72	Both	High-middle SDI	2008	1.7	48.3	1.4	46.8	13.4	3.7	32.7	3.6	0.5	3.6	1.3	66.1
73	Both	Middle SDI	2006	2.2	59.5	1.2	58.3	9.7	3.4	49.8	6.2	0.7	2.8	-0.4	36.8
74	Both	High SDI	2008	1.6	45.4	2.5	42.9	27.9	5.6	17.0	1.6	0.3	5.0	3.1	97.8
75	Both	Low-middle SDI	2008	4.4	122.6	1.8	120.8	13.6	5.8	66.7	9.0	0.8	2.3	-1.7	12.6
76	Both	Low SDI	2008	3.2	86.8	1.2	85.6	8.5	4.0	70.3	10.2	0.8	2.1	-2.2	4.3
77	Both	Middle SDI	2008	2.2	59.6	1.2	58.4	10.2	3.5	48.1	5.9	0.6	2.9	-0.2	39.7
78	Both	High-middle SDI	2010	1.7	47.5	1.5	46.0	13.8	3.8	31.5	3.4	0.5	3.7	1.4	68.1
79	Both	Low-middle SDI	2007	4.4	122.7	1.8	120.9	13.3	5.8	67.4	9.2	0.8	2.3	-1.8	11.5
80	Both	High SDI	2010	1.6	44.6	2.5	42.1	28.2	5.7	16.6	1.6	0.3	5.0	3.2	98.4
81	Both	Low-middle SDI	2010	4.4	122.4	1.9	120.6	14.1	5.9	65.1	8.7	0.8	2.4	-1.6	15.6
82	Both	High-middle SDI	2007	1.7	48.1	1.4	46.7	13.2	3.7	33.1	3.7	0.5	3.6	1.3	65.6
83	Both	High-middle SDI	2009	1.7	47.4	1.4	45.9	13.5	3.7	32.0	3.5	0.5	3.6	1.4	67.2
84	Both	High SDI	2009	1.6	44.8	2.5	42.3	27.9	5.6	16.8	1.6	0.3	5.0	3.2	98.0
85	Both	Middle SDI	2010	2.3	60.2	1.3	59.0	10.8	3.6	46.3	5.6	0.6	3.0	0.0	42.9

86	Both	Low SDI	2010	3.2	86.9	1.2	85.7	8.8	4.0	68.7	9.9	0.8	2.2	-2.1	6.6
87	Both	High SDI	2007	1.7	45.9	2.5	43.4	27.9	5.6	17.3	1.6	0.3	5.0	3.1	97.4
88	Both	Low-middle SDI	2012	4.4	122.8	1.9	120.9	14.7	6.0	63.8	8.4	0.7	2.5	-1.4	18.0
89	Both	Low SDI	2012	3.2	87.2	1.3	85.9	9.1	4.1	67.5	9.6	0.8	2.2	-2.0	8.5
90	Both	High SDI	2012	1.6	43.5	2.5	41.0	27.9	5.6	16.3	1.6	0.3	5.0	3.2	98.7
91	Both	Low-middle SDI	2009	4.4	122.0	1.8	120.2	13.8	5.8	66.0	8.8	0.8	2.4	-1.7	14.1
92	Both	Middle SDI	2009	2.2	59.9	1.2	58.6	10.5	3.5	47.3	5.7	0.6	3.0	-0.1	41.2
93	Both	Low SDI	2009	3.2	87.0	1.2	85.8	8.6	4.0	69.7	10.1	0.8	2.2	-2.2	5.4
94	Both	Low-middle SDI	2011	4.4	122.9	1.9	121.0	14.5	5.9	64.4	8.5	0.7	2.4	-1.5	16.9
95	Both	High-middle SDI	2011	1.7	47.0	1.5	45.5	13.9	3.8	30.9	3.4	0.5	3.7	1.5	69.0
96	Both	Middle SDI	2012	2.3	60.7	1.3	59.3	11.5	3.7	44.5	5.3	0.6	3.1	0.2	45.7
97	Both	High SDI	2011	1.6	44.3	2.5	41.7	28.1	5.7	16.5	1.6	0.3	5.0	3.2	98.5
98	Both	High-middle SDI	2013	1.7	45.8	1.5	44.3	14.2	3.8	29.8	3.2	0.4	3.7	1.6	70.9
99	Both	Low-middle SDI	2013	4.5	123.6	1.9	121.7	15.0	6.1	63.2	8.2	0.7	2.5	-1.4	18.7
100	Both	High SDI	2013	1.6	43.5	2.5	41.0	28.2	5.7	16.2	1.5	0.3	5.0	3.2	98.9
101	Both	Middle SDI	2011	2.3	60.7	1.3	59.4	11.2	3.7	45.4	5.4	0.6	3.0	0.1	44.3
102	Both	Middle SDI	2014	2.3	60.6	1.4	59.2	12.1	3.8	43.0	5.0	0.6	3.1	0.3	48.4
103	Both	Low SDI	2011	3.2	87.0	1.3	85.8	8.9	4.1	68.2	9.7	0.8	2.2	-2.0	7.6
104	Both	Low SDI	2013	3.3	88.3	1.3	87.0	9.3	4.2	66.9	9.5	0.8	2.2	-1.9	9.1
105	Both	High-middle SDI	2012	1.7	46.6	1.5	45.1	14.1	3.8	30.5	3.3	0.4	3.7	1.5	69.8
106	Both	Middle SDI	2013	2.3	60.8	1.4	59.4	11.8	3.8	43.8	5.2	0.6	3.1	0.2	47.1
107	Both	Low-middle SDI	2015	4.5	125.4	2.0	123.4	15.6	6.2	62.0	8.0	0.7	2.5	-1.3	20.4
108	Both	High-middle SDI	2015	1.7	44.8	1.5	43.3	14.5	3.8	28.8	3.1	0.4	3.8	1.7	72.5
109	Both	High-middle SDI	2014	1.7	45.5	1.5	44.0	14.4	3.8	29.3	3.2	0.4	3.8	1.6	71.8
110	Both	Low SDI	2015	3.4	89.9	1.3	88.5	9.7	4.3	65.9	9.3	0.8	2.2	-1.9	10.8
111	Both	High SDI	2015	1.6	43.3	2.6	40.7	28.6	5.7	15.9	1.5	0.3	5.0	3.2	99.3
112	Both	High SDI	2014	1.6	43.3	2.5	40.8	28.3	5.7	16.0	1.5	0.3	5.0	3.2	99.1
113	Both	Middle SDI	2015	2.3	60.8	1.4	59.4	12.3	3.9	42.5	4.9	0.6	3.2	0.4	49.3
114	Both	Low-middle SDI	2014	4.5	124.0	1.9	122.0	15.2	6.1	62.6	8.1	0.7	2.5	-1.4	19.4
115	Both	High-middle SDI	2017	1.7	44.9	1.5	43.3	15.0	3.9	28.0	3.0	0.4	3.9	1.8	74.1
116	Both	Low SDI	2017	3.4	89.9	1.4	88.6	10.0	4.4	64.5	9.0	0.8	2.3	-1.7	13.0
117	Both	Low SDI	2016	3.4	89.6	1.4	88.2	9.8	4.4	65.2	9.1	0.8	2.3	-1.8	11.9
118	Both	Low-middle SDI	2016	4.6	126.6	2.0	124.6	16.0	6.4	61.5	7.9	0.7	2.5	-1.2	21.4
119	Both	High SDI	2017	1.6	42.5	2.6	39.9	28.5	5.7	15.7	1.5	0.3	5.0	3.3	99.7
120	Both	Low-middle SDI	2017	4.6	127.9	2.1	125.9	16.4	6.5	60.7	7.8	0.7	2.5	-1.2	22.5
121	Both	High-middle SDI	2016	1.7	44.7	1.5	43.2	14.7	3.8	28.4	3.0	0.4	3.8	1.7	73.2
122	Both	Middle SDI	2017	2.3	60.8	1.4	59.3	12.8	4.0	41.2	4.7	0.6	3.2	0.5	51.6
123	Both	Low SDI	2014	3.4	89.0	1.3	87.6	9.5	4.3	66.5	9.4	0.8	2.2	-1.9	9.8
124	Both	High SDI	2016	1.6	43.4	2.6	40.8	29.0	5.8	15.7	1.5	0.3	5.0	3.2	99.6
125	Both	Low-middle SDI	2018	4.7	128.7	2.1	126.6	16.8	6.6	60.0	7.7	0.7	2.6	-1.1	23.5
126	Both	High-middle SDI	2018	1.7	45.0	1.6	43.5	15.3	4.0	27.6	2.9	0.4	3.9	1.8	74.8
127	Both	High SDI	2018	1.6	41.9	2.5	39.4	28.1	5.6	15.6	1.5	0.3	5.0	3.3	99.8
128	Both	Middle SDI	2016	2.3	60.9	1.4	59.4	12.5	3.9	42.0	4.9	0.6	3.2	0.4	50.2
129	Both	High SDI	2021	1.5	40.0	2.4	37.6	27.3	5.4	15.4	1.5	0.3	5.0	3.3	100.0

130	Both	Low-middle SDI	2021	4.7	128.4	2.2	126.2	17.7	6.7	57.9	7.3	0.7	2.6	-0.9	27.0
131	Both	Low SDI	2021	3.4	90.5	1.4	89.1	10.8	4.6	61.9	8.4	0.7	2.4	-1.5	17.6
132	Both	Middle SDI	2018	2.3	60.9	1.5	59.5	13.2	4.0	40.5	4.6	0.6	3.3	0.6	52.9
133	Both	Low SDI	2018	3.4	90.1	1.4	88.7	10.2	4.4	63.8	8.8	0.8	2.3	-1.7	14.1
134	Both	High-middle SDI	2019	1.6	44.7	1.6	43.1	15.4	4.0	27.3	2.9	0.4	3.9	1.9	75.3
135	Both	Middle SDI	2021	2.3	61.1	1.5	59.6	13.9	4.2	39.0	4.4	0.5	3.3	0.7	55.6
136	Both	High SDI	2019	1.6	41.6	2.5	39.1	28.1	5.6	15.6	1.5	0.3	5.0	3.3	99.8
137	Both	Low-middle SDI	2019	4.7	128.6	2.1	126.5	17.1	6.6	59.1	7.5	0.7	2.6	-1.0	24.8
138	Both	Low SDI	2019	3.4	90.3	1.4	88.9	10.4	4.5	63.1	8.7	0.8	2.3	-1.6	15.4
139	Both	Middle SDI	2019	2.3	61.0	1.5	59.5	13.5	4.1	39.8	4.5	0.6	3.3	0.6	54.1
140	Both	High-middle SDI	1996	2.0	56.4	1.4	55.0	12.3	3.8	39.3	4.6	0.5	3.2	0.7	55.1
141	Both	High SDI	1996	1.9	52.1	2.5	49.6	26.3	5.7	20.1	2.0	0.3	4.6	2.8	91.0
142	Both	High-middle SDI	2021	1.6	43.3	1.5	41.8	15.1	3.9	27.0	2.9	0.4	3.9	1.9	75.9
143	Both	Low-middle SDI	1996	4.5	123.2	1.7	121.5	12.4	5.6	70.5	9.9	0.8	2.2	-2.1	6.1
144	Both	Middle SDI	2002	2.3	60.4	1.1	59.3	9.1	3.3	52.6	6.6	0.7	2.7	-0.6	32.3
145	Both	Middle SDI	1996	2.3	62.6	1.1	61.5	8.7	3.3	55.4	7.2	0.7	2.6	-0.9	27.5
146	Both	Low SDI	1996	3.4	92.2	1.2	91.0	8.5	4.1	72.9	10.9	0.8	2.0	-2.5	0.0
147	Both	High-middle SDI	1993	2.1	58.5	1.4	57.1	12.3	3.9	40.5	4.8	0.5	3.2	0.6	53.2
148	Both	High SDI	1993	1.9	52.9	2.4	50.5	25.4	5.5	21.2	2.1	0.3	4.7	2.7	89.7
149	Both	Low SDI	1993	3.3	89.9	1.2	88.7	8.3	4.0	72.7	10.9	0.8	2.1	-2.4	0.6
150	Both	High-middle SDI	2002	1.9	52.5	1.4	51.1	12.5	3.6	37.3	4.2	0.5	3.4	0.9	58.9
151	Both	Low-middle SDI	1993	4.3	120.3	1.7	118.6	12.0	5.5	70.6	10.0	0.8	2.2	-2.1	6.1
152	Both	Middle SDI	1993	2.3	61.8	1.1	60.7	8.3	3.2	56.7	7.5	0.7	2.6	-1.0	25.6
153	Both	High SDI	2002	1.8	49.2	2.5	46.8	27.4	5.6	18.8	1.8	0.3	4.9	3.0	95.0
154	Both	Low SDI	2002	3.3	91.6	1.2	90.4	8.5	4.1	72.4	10.8	0.8	2.1	-2.4	1.1
155	Both	Low-middle SDI	2002	4.5	123.8	1.8	122.0	12.7	5.7	69.7	9.7	0.8	2.2	-2.0	7.9
156	Both	Low SDI	2020	3.4	90.5	1.4	89.0	10.7	4.5	62.3	8.5	0.8	2.4	-1.5	16.8
157	Both	Low-middle SDI	2020	4.7	128.6	2.2	126.4	17.5	6.7	58.4	7.3	0.7	2.6	-1.0	26.3
158	Both	High-middle SDI	2020	1.6	43.7	1.6	42.1	15.1	3.9	27.2	2.9	0.4	3.9	1.9	75.4
159	Both	High SDI	2020	1.5	40.2	2.4	37.8	27.3	5.4	15.5	1.5	0.3	5.0	3.3	99.9
160	Both	Middle SDI	2020	2.3	61.0	1.5	59.5	13.8	4.1	39.3	4.4	0.6	3.4	0.7	55.2
161	Male	Middle SDI	1991	3.2	86.9	1.4	85.5	10.1	4.3	62.1	8.6	0.7	2.4	-1.2	21.9
162	Male	High-middle SDI	1990	3.4	94.5	2.2	92.4	18.3	6.1	42.8	5.2	0.6	3.0	0.6	52.6
163	Male	High-middle SDI	1992	3.4	96.8	2.2	94.5	19.1	6.3	42.3	5.1	0.5	3.0	0.7	53.7
164	Male	High SDI	1992	3.1	86.3	3.6	82.8	37.4	8.3	23.1	2.3	0.4	4.5	2.7	89.0
165	Male	High SDI	1990	3.2	88.4	3.6	84.8	37.8	8.5	23.4	2.3	0.4	4.5	2.6	88.4
166	Male	Low-middle SDI	1992	5.4	149.7	2.0	147.7	13.8	6.6	74.1	10.8	0.8	2.1	-2.1	5.9
167	Male	Low-middle SDI	1990	5.4	150.5	2.0	148.5	13.7	6.6	74.7	11.0	0.8	2.1	-2.1	4.9
168	Male	Low SDI	1992	3.9	108.8	1.4	107.4	9.3	4.7	75.6	11.7	0.8	2.0	-2.4	0.9
169	Male	Middle SDI	1992	3.2	87.2	1.4	85.8	10.3	4.3	61.6	8.5	0.7	2.4	-1.1	22.8
170	Male	Middle SDI	1990	3.2	86.4	1.4	85.0	9.9	4.2	62.5	8.7	0.8	2.4	-1.2	21.0
171	Male	Low SDI	1991	3.9	108.4	1.4	107.0	9.2	4.7	75.9	11.7	0.8	2.0	-2.4	0.7
172	Male	Low SDI	1990	3.9	108.0	1.4	106.6	9.2	4.7	75.9	11.8	0.8	2.0	-2.4	0.6
173	Male	High-middle SDI	1991	3.4	94.9	2.2	92.7	18.6	6.1	42.4	5.1	0.5	3.0	0.6	53.4

174	Male	High-middle SDI	1994	3.5	99.7	2.3	97.4	19.5	6.5	42.3	5.1	0.5	3.0	0.6	53.6
175	Male	High SDI	1991	3.1	87.9	3.6	84.3	37.8	8.4	23.3	2.3	0.4	4.5	2.7	88.7
176	Male	Low-middle SDI	1991	5.3	149.7	2.0	147.7	13.7	6.6	74.4	10.9	0.8	2.1	-2.1	5.5
177	Male	High SDI	1994	3.0	84.4	3.6	80.9	37.6	8.4	22.5	2.2	0.4	4.5	2.7	89.9
178	Male	Low SDI	1994	4.1	112.0	1.5	110.6	9.5	4.9	75.8	11.8	0.8	1.9	-2.4	0.2
179	Male	High-middle SDI	1997	3.3	92.0	2.2	89.8	19.0	6.1	40.8	4.8	0.5	3.1	0.8	56.0
180	Male	Low-middle SDI	1994	5.5	151.6	2.0	149.6	14.1	6.7	73.7	10.8	0.8	2.1	-2.1	6.1
181	Male	High SDI	1997	2.9	81.9	3.6	78.3	38.2	8.5	21.5	2.1	0.3	4.5	2.8	91.2
182	Male	Middle SDI	1994	3.3	89.0	1.4	87.5	10.8	4.5	60.5	8.3	0.7	2.4	-1.0	24.1
183	Male	Low-middle SDI	1997	5.6	153.1	2.1	151.0	14.2	6.8	73.7	10.7	0.8	2.1	-2.1	6.0
184	Male	Low SDI	1997	4.1	112.9	1.5	111.4	9.6	4.9	76.1	11.8	0.8	2.0	-2.4	0.1
185	Male	Middle SDI	1997	3.3	88.7	1.5	87.2	11.1	4.5	59.2	8.0	0.7	2.5	-0.9	26.1
186	Male	Low-middle SDI	1995	5.5	152.4	2.0	150.4	14.2	6.8	73.6	10.8	0.8	2.1	-2.1	6.1
187	Male	Low-middle SDI	1998	5.6	153.5	2.1	151.4	14.3	6.8	73.5	10.7	0.8	2.1	-2.1	6.3
188	Male	Low SDI	1995	4.1	112.4	1.5	111.0	9.5	4.9	75.7	11.8	0.8	2.0	-2.4	0.3
189	Male	High-middle SDI	1995	3.4	97.6	2.3	95.3	19.4	6.4	41.7	5.0	0.5	3.0	0.7	54.3
190	Male	Low SDI	1998	4.2	113.7	1.5	112.2	9.6	4.9	76.3	11.8	0.8	2.0	-2.4	0.0
191	Male	High SDI	1995	3.0	84.8	3.7	81.1	38.4	8.6	22.1	2.2	0.4	4.5	2.7	90.3
192	Male	High SDI	1999	2.8	79.6	3.6	76.0	37.8	8.3	21.2	2.1	0.3	4.5	2.8	92.1
193	Male	High-middle SDI	1998	3.2	89.5	2.1	87.3	18.6	6.0	40.6	4.8	0.5	3.1	0.8	56.1
194	Male	Low-middle SDI	1999	5.5	153.3	2.1	151.2	14.4	6.8	73.6	10.7	0.8	2.1	-2.0	6.6
195	Male	Middle SDI	1995	3.3	89.4	1.5	87.9	10.9	4.5	60.2	8.2	0.7	2.4	-1.0	24.6
196	Male	High SDI	1998	2.9	81.3	3.6	77.6	38.2	8.5	21.3	2.1	0.3	4.5	2.8	91.6
197	Male	Low SDI	1999	4.1	113.0	1.5	111.6	9.6	4.9	76.1	11.8	0.8	2.0	-2.4	0.1
198	Male	Middle SDI	1999	3.2	87.7	1.5	86.3	11.3	4.5	58.3	7.8	0.7	2.5	-0.8	27.8
199	Male	Low SDI	2001	4.1	112.9	1.5	111.4	9.6	4.9	76.1	11.7	0.8	2.0	-2.4	0.3
200	Male	Middle SDI	1998	3.3	88.3	1.5	86.8	11.2	4.5	58.7	7.9	0.7	2.5	-0.9	26.9
201	Male	High-middle SDI	2000	3.2	89.9	2.2	87.7	18.8	6.0	40.5	4.8	0.5	3.1	0.8	56.4
202	Male	Middle SDI	2001	3.2	87.7	1.5	86.2	11.6	4.5	57.2	7.6	0.7	2.5	-0.7	29.3
203	Male	Low-middle SDI	2000	5.5	153.8	2.1	151.7	14.4	6.8	73.4	10.6	0.8	2.1	-2.0	6.9
204	Male	Low SDI	2000	4.1	113.4	1.5	111.9	9.6	4.9	76.3	11.8	0.8	2.0	-2.4	0.1
205	Male	High SDI	2000	2.8	79.2	3.6	75.6	38.5	8.3	21.0	2.1	0.3	4.6	2.9	93.1
206	Male	High-middle SDI	1999	3.2	90.5	2.2	88.3	18.8	6.0	40.7	4.8	0.5	3.1	0.8	56.1
207	Male	High-middle SDI	2003	3.1	86.0	2.1	83.9	18.7	5.8	39.4	4.6	0.5	3.2	0.9	58.4
208	Male	Middle SDI	2000	3.2	87.5	1.5	86.1	11.4	4.5	57.8	7.7	0.7	2.5	-0.8	28.6
209	Male	High SDI	2003	2.7	76.9	3.6	73.3	39.4	8.2	20.1	2.0	0.3	4.8	3.0	95.3
210	Male	Low-middle SDI	2003	5.5	153.4	2.1	151.3	14.6	6.9	72.7	10.5	0.8	2.1	-2.0	7.7
211	Male	Low SDI	2003	4.1	111.0	1.4	109.5	9.5	4.8	75.6	11.7	0.8	2.0	-2.4	0.8
212	Male	High-middle SDI	2001	3.2	88.8	2.2	86.6	18.9	5.9	40.1	4.7	0.5	3.2	0.9	57.2
213	Male	High SDI	2001	2.8	79.1	3.7	75.5	39.4	8.4	20.6	2.0	0.3	4.7	3.0	94.2
214	Male	Middle SDI	2003	3.2	87.6	1.5	86.1	11.9	4.6	56.2	7.4	0.7	2.6	-0.6	31.1
215	Male	Low SDI	2005	4.0	109.4	1.4	107.9	9.5	4.8	75.2	11.5	0.8	2.0	-2.3	1.7
216	Male	Low-middle SDI	2001	5.5	153.5	2.1	151.4	14.4	6.8	73.3	10.6	0.8	2.1	-2.0	7.0
217	Male	Low-middle SDI	2005	5.5	152.8	2.1	150.7	14.8	6.9	71.9	10.3	0.8	2.2	-1.9	9.1

218	Male	High SDI	2005	2.6	73.1	3.6	69.5	38.9	8.1	19.4	1.9	0.3	4.8	3.1	96.5
219	Male	High-middle SDI	2004	3.0	84.4	2.1	82.3	18.8	5.8	38.7	4.5	0.5	3.2	1.0	59.4
220	Male	High SDI	2004	2.6	74.6	3.6	71.0	38.9	8.1	19.8	1.9	0.3	4.8	3.1	95.9
221	Male	Low-middle SDI	2004	5.5	152.5	2.1	150.4	14.6	6.8	72.4	10.4	0.8	2.1	-1.9	8.3
222	Male	Middle SDI	2005	3.2	87.4	1.6	85.8	12.3	4.7	54.7	7.1	0.7	2.6	-0.5	33.5
223	Male	Middle SDI	2004	3.2	87.3	1.5	85.8	12.0	4.6	55.5	7.3	0.7	2.6	-0.6	32.1
224	Male	Low SDI	2004	4.0	110.6	1.4	109.2	9.5	4.8	75.6	11.6	0.8	2.0	-2.3	1.2
225	Male	Low SDI	2007	4.0	108.2	1.4	106.8	9.6	4.8	74.3	11.3	0.8	2.0	-2.2	2.9
226	Male	High-middle SDI	2006	2.9	80.8	2.1	78.7	19.1	5.8	36.7	4.2	0.5	3.3	1.1	62.3
227	Male	High SDI	2006	2.6	72.6	3.6	68.9	39.3	8.2	19.0	1.8	0.3	4.8	3.1	96.9
228	Male	Low-middle SDI	2006	5.5	152.8	2.1	150.7	15.0	6.9	71.6	10.2	0.8	2.2	-1.8	9.9
229	Male	Middle SDI	2007	3.2	87.4	1.6	85.8	13.0	4.8	52.8	6.7	0.7	2.7	-0.3	36.6
230	Male	High-middle SDI	2005	3.0	84.2	2.2	82.0	19.1	5.9	37.8	4.4	0.5	3.2	1.0	60.5
231	Male	Low SDI	2006	4.0	108.2	1.4	106.7	9.5	4.7	74.8	11.4	0.8	2.0	-2.3	2.2
232	Male	High-middle SDI	2008	2.9	79.2	2.2	77.1	19.5	5.8	35.4	4.1	0.5	3.3	1.3	64.2
233	Male	Middle SDI	2006	3.2	87.1	1.6	85.5	12.6	4.7	53.7	6.9	0.7	2.7	-0.4	35.1
234	Male	High SDI	2008	2.5	70.7	3.6	67.1	39.5	8.2	18.5	1.8	0.3	4.8	3.2	97.7
235	Male	Low-middle SDI	2008	5.6	154.7	2.2	152.5	15.6	7.0	70.3	9.9	0.8	2.2	-1.7	11.9
236	Male	Low SDI	2008	3.9	107.2	1.4	105.8	9.6	4.7	73.8	11.2	0.8	2.0	-2.2	3.8
237	Male	Middle SDI	2008	3.2	88.2	1.7	86.5	13.4	4.9	51.8	6.6	0.7	2.7	-0.2	38.2
238	Male	High-middle SDI	2010	2.8	77.7	2.2	75.5	20.0	5.9	34.2	3.9	0.5	3.4	1.4	66.1
239	Male	Low-middle SDI	2007	5.5	153.9	2.1	151.8	15.3	7.0	70.9	10.0	0.8	2.2	-1.8	10.9
240	Male	High SDI	2010	2.5	69.1	3.6	65.5	39.5	8.2	18.1	1.7	0.3	4.8	3.2	98.3
241	Male	Low-middle SDI	2010	5.5	154.5	2.2	152.3	16.3	7.1	68.7	9.5	0.8	2.3	-1.6	14.8
242	Male	High-middle SDI	2007	2.8	78.9	2.2	76.8	19.3	5.8	35.7	4.1	0.5	3.3	1.2	63.8
243	Male	High-middle SDI	2009	2.8	77.4	2.2	75.3	19.5	5.8	34.7	4.0	0.5	3.4	1.3	65.3
244	Male	High SDI	2009	2.5	69.6	3.6	66.0	39.3	8.2	18.3	1.8	0.3	4.8	3.2	98.0
245	Male	Middle SDI	2010	3.3	89.6	1.8	87.9	14.5	5.1	49.7	6.2	0.6	2.8	0.0	41.6
246	Male	Low SDI	2010	4.0	107.9	1.5	106.4	10.0	4.8	72.3	10.8	0.8	2.1	-2.1	6.1
247	Male	High SDI	2007	2.6	71.8	3.6	68.1	39.6	8.2	18.7	1.8	0.3	4.8	3.2	97.4
248	Male	Low-middle SDI	2012	5.5	154.1	2.3	151.8	16.8	7.2	67.4	9.2	0.8	2.3	-1.4	17.2
249	Male	Low SDI	2012	3.9	107.6	1.5	106.1	10.2	4.9	71.2	10.5	0.8	2.1	-2.0	8.0
250	Male	High SDI	2012	2.4	67.0	3.6	63.4	39.0	8.1	17.8	1.7	0.3	4.8	3.2	98.6
251	Male	Low-middle SDI	2009	5.5	153.9	2.2	151.8	15.9	7.0	69.6	9.7	0.8	2.3	-1.7	13.3
252	Male	Middle SDI	2009	3.3	88.8	1.7	87.1	13.9	5.0	50.8	6.4	0.7	2.8	-0.1	39.8
253	Male	Low SDI	2009	3.9	107.6	1.4	106.2	9.8	4.8	73.2	11.0	0.8	2.0	-2.1	4.9
254	Male	Low-middle SDI	2011	5.5	155.0	2.2	152.7	16.6	7.2	68.0	9.3	0.8	2.3	-1.5	16.0
255	Male	High-middle SDI	2011	2.8	76.8	2.2	74.6	20.2	5.9	33.5	3.8	0.5	3.4	1.4	67.0
256	Male	Middle SDI	2012	3.3	90.9	1.9	89.0	15.5	5.4	47.6	5.9	0.6	2.9	0.1	44.7
257	Male	High SDI	2011	2.5	68.4	3.6	64.8	39.5	8.2	17.9	1.7	0.3	4.8	3.2	98.4
258	Male	High-middle SDI	2013	2.7	74.7	2.2	72.5	20.5	5.9	32.3	3.6	0.5	3.5	1.5	68.8
259	Male	Low-middle SDI	2013	5.5	154.3	2.3	152.0	17.0	7.2	66.8	9.1	0.8	2.4	-1.4	17.9
260	Male	High SDI	2013	2.4	66.7	3.6	63.1	39.3	8.1	17.6	1.7	0.3	4.8	3.2	98.8
261	Male	Middle SDI	2011	3.3	90.6	1.8	88.8	15.1	5.3	48.6	6.0	0.6	2.9	0.1	43.2

262	Male	Middle SDI	2014	3.4	90.9	1.9	88.9	16.3	5.5	45.9	5.6	0.6	3.0	0.3	47.4
263	Male	Low SDI	2011	4.0	108.0	1.5	106.5	10.1	4.9	71.8	10.7	0.8	2.1	-2.0	7.1
264	Male	Low SDI	2013	4.0	108.9	1.5	107.4	10.5	5.0	70.6	10.4	0.8	2.1	-1.9	8.6
265	Male	High-middle SDI	2012	2.8	76.2	2.2	74.0	20.4	5.9	33.1	3.7	0.5	3.4	1.5	67.6
266	Male	Middle SDI	2013	3.4	91.2	1.9	89.3	16.0	5.4	46.8	5.7	0.6	2.9	0.2	46.1
267	Male	Low-middle SDI	2015	5.6	155.4	2.3	153.1	17.5	7.4	65.6	8.9	0.8	2.4	-1.3	19.4
268	Male	High-middle SDI	2015	2.7	72.5	2.2	70.2	20.6	5.9	31.4	3.5	0.5	3.5	1.6	70.1
269	Male	High-middle SDI	2014	2.7	74.0	2.3	71.8	20.7	5.9	31.9	3.6	0.5	3.5	1.6	69.5
270	Male	Low SDI	2015	4.1	110.2	1.6	108.6	10.9	5.1	69.4	10.2	0.8	2.1	-1.8	10.2
271	Male	High SDI	2015	2.4	66.1	3.6	62.4	39.7	8.2	17.2	1.7	0.3	4.8	3.3	99.3
272	Male	High SDI	2014	2.4	66.3	3.6	62.7	39.4	8.1	17.4	1.7	0.3	4.8	3.2	99.1
273	Male	Middle SDI	2015	3.4	91.0	2.0	89.0	16.6	5.6	45.4	5.5	0.6	3.0	0.3	48.2
274	Male	Low-middle SDI	2014	5.6	154.6	2.3	152.3	17.3	7.3	66.2	9.0	0.8	2.4	-1.4	18.5
275	Male	High-middle SDI	2017	2.6	71.8	2.3	69.6	21.0	5.9	30.6	3.4	0.4	3.5	1.7	71.5
276	Male	Low SDI	2017	4.1	109.5	1.6	107.9	11.1	5.1	68.2	9.9	0.8	2.2	-1.7	12.1
277	Male	Low SDI	2016	4.1	109.4	1.6	107.8	10.9	5.1	68.7	10.0	0.8	2.1	-1.8	11.2
278	Male	Low-middle SDI	2016	5.6	156.7	2.4	154.3	17.9	7.5	65.1	8.7	0.7	2.4	-1.2	20.4
279	Male	High SDI	2017	2.4	64.3	3.6	60.7	39.2	8.1	17.0	1.6	0.3	4.9	3.3	99.7
280	Male	Low-middle SDI	2017	5.7	158.0	2.4	155.6	18.3	7.6	64.3	8.6	0.7	2.4	-1.2	21.3
281	Male	High-middle SDI	2016	2.7	72.1	2.2	69.8	20.8	5.9	31.0	3.5	0.5	3.5	1.6	70.7
282	Male	Middle SDI	2017	3.3	90.3	2.0	88.3	17.2	5.6	44.2	5.3	0.6	3.1	0.5	50.5
283	Male	Low SDI	2014	4.1	109.1	1.5	107.5	10.6	5.0	70.0	10.3	0.8	2.1	-1.9	9.4
284	Male	High SDI	2016	2.4	65.8	3.6	62.2	39.9	8.2	17.1	1.7	0.3	4.9	3.3	99.5
285	Male	Low-middle SDI	2018	5.7	159.1	2.5	156.6	18.7	7.7	63.5	8.5	0.7	2.4	-1.1	22.4
286	Male	High-middle SDI	2018	2.6	72.1	2.3	69.8	21.4	6.0	30.2	3.4	0.4	3.5	1.7	72.2
287	Male	High SDI	2018	2.3	63.2	3.5	59.7	38.5	7.9	17.0	1.6	0.3	4.9	3.3	99.7
288	Male	Middle SDI	2016	3.4	90.9	2.0	88.9	16.8	5.6	44.9	5.4	0.6	3.0	0.4	49.0
289	Male	High SDI	2021	2.3	60.3	3.4	56.9	37.3	7.7	16.7	1.6	0.3	4.9	3.3	100.0
290	Male	Low-middle SDI	2021	5.8	160.6	2.6	158.0	19.9	8.0	61.6	8.1	0.7	2.5	-1.0	25.5
291	Male	Low SDI	2021	4.1	111.5	1.7	109.8	12.0	5.4	65.5	9.3	0.8	2.2	-1.5	16.5
292	Male	Middle SDI	2018	3.3	90.4	2.0	88.3	17.6	5.7	43.3	5.1	0.6	3.1	0.5	51.8
293	Male	Low SDI	2018	4.1	109.7	1.6	108.1	11.3	5.2	67.4	9.7	0.8	2.2	-1.7	13.3
294	Male	High-middle SDI	2019	2.6	71.2	2.3	68.9	21.4	6.0	29.9	3.3	0.4	3.6	1.7	72.6
295	Male	Middle SDI	2021	3.3	90.7	2.1	88.6	18.6	5.9	41.8	4.9	0.6	3.2	0.7	54.4
296	Male	High SDI	2019	2.3	62.6	3.5	59.1	38.4	7.9	16.9	1.6	0.3	4.9	3.3	99.8
297	Male	Low-middle SDI	2019	5.7	159.4	2.5	156.9	19.1	7.8	62.7	8.3	0.7	2.4	-1.1	23.6
298	Male	Low SDI	2019	4.1	110.4	1.6	108.7	11.6	5.2	66.7	9.5	0.8	2.2	-1.6	14.5
299	Male	Middle SDI	2019	3.3	90.5	2.1	88.4	18.0	5.8	42.6	5.0	0.6	3.1	0.6	52.9
300	Male	High-middle SDI	1996	3.4	94.5	2.2	92.3	19.1	6.3	41.2	4.9	0.5	3.1	0.7	55.1
301	Male	High SDI	1996	3.0	83.5	3.7	79.8	38.5	8.6	21.7	2.2	0.3	4.5	2.8	90.8
302	Male	High-middle SDI	2021	2.5	68.6	2.2	66.3	20.9	5.8	29.6	3.3	0.4	3.6	1.8	73.2
303	Male	Low-middle SDI	1996	5.5	152.1	2.0	150.0	14.1	6.7	73.6	10.7	0.8	2.1	-2.1	6.1
304	Male	Middle SDI	2002	3.2	87.2	1.5	85.7	11.7	4.5	56.7	7.5	0.7	2.6	-0.7	30.2
305	Male	Middle SDI	1996	3.3	89.2	1.5	87.8	11.0	4.5	59.7	8.1	0.7	2.4	-1.0	25.4

306	Male	Low SDI	1996	4.1	111.9	1.5	110.5	9.5	4.9	76.0	11.8	0.8	2.0	-2.4	0.1
307	Male	High-middle SDI	1993	3.5	99.2	2.3	97.0	19.4	6.5	42.4	5.1	0.5	3.0	0.6	53.5
308	Male	High SDI	1993	3.0	85.6	3.6	82.0	37.7	8.4	22.8	2.3	0.4	4.5	2.7	89.6
309	Male	Low SDI	1993	4.0	109.9	1.4	108.4	9.4	4.8	75.7	11.7	0.8	2.0	-2.4	0.8
310	Male	High-middle SDI	2002	3.1	87.4	2.1	85.3	18.8	5.9	39.8	4.6	0.5	3.2	0.9	57.9
311	Male	Low-middle SDI	1993	5.4	149.6	2.0	147.6	13.9	6.6	73.9	10.8	0.8	2.1	-2.1	6.1
312	Male	Middle SDI	1993	3.2	87.7	1.4	86.3	10.5	4.4	61.0	8.4	0.7	2.4	-1.1	23.6
313	Male	High SDI	2002	2.8	78.0	3.7	74.3	39.6	8.3	20.3	2.0	0.3	4.8	3.0	94.9
314	Male	Low SDI	2002	4.1	111.4	1.4	109.9	9.5	4.8	75.8	11.7	0.8	2.0	-2.4	0.6
315	Male	Low-middle SDI	2002	5.5	153.2	2.1	151.1	14.5	6.8	73.1	10.6	0.8	2.1	-2.0	7.3
316	Male	Low SDI	2020	4.1	111.1	1.7	109.4	11.9	5.3	65.9	9.3	0.8	2.2	-1.5	15.8
317	Male	Low-middle SDI	2020	5.8	160.2	2.5	157.7	19.7	7.9	62.0	8.1	0.7	2.5	-1.0	24.9
318	Male	High-middle SDI	2020	2.6	69.6	2.3	67.3	21.0	5.9	29.8	3.3	0.4	3.6	1.7	72.8
319	Male	High SDI	2020	2.3	60.6	3.4	57.2	37.3	7.7	16.8	1.6	0.3	4.9	3.3	99.9
320	Male	Middle SDI	2020	3.3	90.6	2.1	88.5	18.4	5.8	42.1	4.9	0.6	3.2	0.7	54.0
321	Female	Middle SDI	1991	1.4	36.0	0.7	35.2	5.8	2.1	49.4	6.2	0.7	2.8	-0.9	29.3
322	Female	High-middle SDI	1990	0.9	22.3	0.6	21.7	5.6	1.6	35.0	3.9	0.5	3.5	0.5	53.9
323	Female	High-middle SDI	1992	0.9	22.5	0.6	21.9	5.9	1.7	33.9	3.8	0.5	3.6	0.6	55.9
324	Female	High SDI	1992	0.9	24.0	1.3	22.7	14.3	3.0	17.3	1.7	0.3	4.8	2.5	89.7
325	Female	High SDI	1990	1.0	24.2	1.3	22.9	14.0	2.9	17.7	1.7	0.3	4.8	2.5	88.8
326	Female	Low-middle SDI	1992	3.2	89.7	1.3	88.3	10.0	4.3	65.8	9.0	0.8	2.3	-2.2	6.4
327	Female	Low-middle SDI	1990	3.2	88.1	1.3	86.8	9.7	4.2	66.2	9.1	0.8	2.3	-2.3	5.3
328	Female	Low SDI	1992	2.5	68.5	1.0	67.5	7.0	3.2	68.2	9.8	0.8	2.2	-2.5	0.6
329	Female	Middle SDI	1992	1.4	36.3	0.7	35.6	6.0	2.1	48.8	6.1	0.7	2.8	-0.9	30.2
330	Female	Middle SDI	1990	1.4	36.0	0.7	35.3	5.7	2.1	50.0	6.3	0.7	2.8	-1.0	28.2
331	Female	Low SDI	1991	2.4	67.6	1.0	66.6	6.9	3.2	68.2	9.8	0.8	2.2	-2.5	0.5
332	Female	Low SDI	1990	2.4	66.9	1.0	65.9	6.8	3.1	68.3	9.8	0.8	2.2	-2.6	0.3
333	Female	High-middle SDI	1991	0.9	22.4	0.6	21.7	5.8	1.6	34.5	3.9	0.5	3.5	0.5	54.8
334	Female	High-middle SDI	1994	0.9	22.9	0.7	22.2	6.2	1.7	33.2	3.7	0.5	3.6	0.7	57.1
335	Female	High SDI	1991	0.9	23.9	1.3	22.6	14.1	2.9	17.5	1.7	0.3	4.8	2.5	89.3
336	Female	Low-middle SDI	1991	3.2	88.3	1.3	87.0	9.8	4.2	66.1	9.0	0.8	2.3	-2.2	5.9
337	Female	High SDI	1994	0.9	23.8	1.3	22.5	14.6	3.0	16.8	1.6	0.3	4.8	2.5	90.6
338	Female	Low SDI	1994	2.6	71.0	1.0	69.9	7.2	3.4	67.9	9.8	0.8	2.2	-2.6	0.1
339	Female	High-middle SDI	1997	0.9	22.1	0.7	21.4	6.3	1.7	31.8	3.5	0.5	3.7	0.8	59.8
340	Female	Low-middle SDI	1994	3.3	91.8	1.4	90.4	10.3	4.4	65.7	8.9	0.8	2.3	-2.2	6.5
341	Female	High SDI	1997	1.0	24.0	1.4	22.6	15.5	3.2	16.0	1.5	0.3	4.8	2.6	91.9
342	Female	Middle SDI	1994	1.5	36.6	0.7	35.9	6.2	2.2	47.9	5.9	0.7	2.8	-0.8	31.6
343	Female	Low-middle SDI	1997	3.4	94.1	1.4	92.7	10.7	4.5	65.9	8.8	0.8	2.4	-2.2	6.8
344	Female	Low SDI	1997	2.6	71.9	1.0	70.9	7.4	3.4	68.4	9.7	0.8	2.2	-2.6	0.4
345	Female	Middle SDI	1997	1.4	36.1	0.8	35.4	6.4	2.2	46.5	5.6	0.7	2.9	-0.6	34.1
346	Female	Low-middle SDI	1995	3.4	93.4	1.4	92.0	10.5	4.5	65.7	8.9	0.8	2.3	-2.2	6.3
347	Female	Low-middle SDI	1998	3.4	94.6	1.4	93.2	10.8	4.5	65.6	8.8	0.8	2.4	-2.2	7.2
348	Female	Low SDI	1995	2.7	72.0	1.0	70.9	7.4	3.4	68.0	9.8	0.8	2.2	-2.6	0.0
349	Female	High-middle SDI	1995	0.9	22.8	0.7	22.1	6.3	1.7	32.8	3.6	0.5	3.6	0.7	58.0

350	Female	Low SDI	1998	2.6	72.1	1.0	71.1	7.4	3.4	68.5	9.7	0.8	2.2	-2.5	0.7
351	Female	High SDI	1995	1.0	24.0	1.4	22.6	15.0	3.1	16.5	1.6	0.3	4.8	2.6	90.9
352	Female	High SDI	1999	0.9	23.7	1.4	22.3	15.7	3.2	15.6	1.5	0.3	4.9	2.7	92.7
353	Female	High-middle SDI	1998	0.9	21.9	0.7	21.2	6.4	1.7	31.3	3.4	0.5	3.7	0.9	60.8
354	Female	Low-middle SDI	1999	3.4	94.3	1.4	92.9	10.9	4.5	65.4	8.7	0.8	2.4	-2.1	7.8
355	Female	Middle SDI	1995	1.5	36.5	0.8	35.8	6.3	2.2	47.5	5.8	0.7	2.9	-0.8	32.1
356	Female	High SDI	1998	1.0	23.8	1.4	22.4	15.6	3.2	15.8	1.5	0.3	4.8	2.6	92.1
357	Female	Low SDI	1999	2.6	71.7	1.0	70.7	7.4	3.3	68.1	9.6	0.8	2.2	-2.5	1.2
358	Female	Middle SDI	1999	1.4	35.6	0.8	34.9	6.5	2.2	45.6	5.4	0.6	3.0	-0.5	35.9
359	Female	Low SDI	2001	2.6	71.1	1.0	70.1	7.4	3.3	67.8	9.6	0.8	2.2	-2.5	1.6
360	Female	Middle SDI	1998	1.4	35.9	0.8	35.1	6.5	2.2	46.1	5.5	0.7	3.0	-0.6	35.0
361	Female	High-middle SDI	2000	0.9	22.0	0.7	21.3	6.8	1.7	30.5	3.3	0.5	3.9	1.0	63.0
362	Female	Middle SDI	2001	1.4	34.8	0.8	34.0	6.6	2.2	44.7	5.3	0.6	3.0	-0.5	37.3
363	Female	Low-middle SDI	2000	3.4	93.9	1.4	92.5	10.9	4.5	65.2	8.7	0.7	2.4	-2.1	8.2
364	Female	Low SDI	2000	2.6	71.4	1.0	70.4	7.4	3.3	68.1	9.6	0.8	2.2	-2.5	1.4
365	Female	High SDI	2000	0.9	23.7	1.4	22.2	16.1	3.2	15.4	1.5	0.3	5.0	2.7	93.8
366	Female	High-middle SDI	1999	0.9	22.0	0.7	21.3	6.6	1.7	31.0	3.3	0.5	3.8	0.9	61.7
367	Female	High-middle SDI	2003	0.8	21.2	0.7	20.5	6.9	1.7	29.0	3.0	0.5	4.0	1.2	66.1
368	Female	Middle SDI	2000	1.4	35.2	0.8	34.5	6.6	2.2	45.1	5.4	0.6	3.0	-0.5	36.8
369	Female	High SDI	2003	0.9	22.6	1.4	21.2	16.2	3.2	14.7	1.4	0.3	5.1	2.8	95.9
370	Female	Low-middle SDI	2003	3.4	94.2	1.4	92.8	11.1	4.6	64.2	8.5	0.7	2.4	-2.0	9.3
371	Female	Low SDI	2003	2.6	71.8	1.0	70.8	7.6	3.4	67.4	9.5	0.8	2.2	-2.5	2.0
372	Female	High-middle SDI	2001	0.9	21.6	0.7	20.9	6.8	1.7	30.0	3.2	0.5	3.9	1.0	64.0
373	Female	High SDI	2001	0.9	23.3	1.4	21.8	16.1	3.2	15.2	1.4	0.3	5.0	2.8	94.7
374	Female	Middle SDI	2003	1.4	33.8	0.8	33.0	6.6	2.1	43.8	5.1	0.6	3.1	-0.4	38.8
375	Female	Low SDI	2005	2.6	69.9	1.0	68.9	7.5	3.3	66.9	9.3	0.8	2.2	-2.4	3.1
376	Female	Low-middle SDI	2001	3.4	94.2	1.4	92.8	10.9	4.5	64.9	8.6	0.7	2.4	-2.1	8.6
377	Female	Low-middle SDI	2005	3.4	94.1	1.5	92.6	11.3	4.6	63.5	8.3	0.7	2.4	-2.0	10.6
378	Female	High SDI	2005	0.9	22.1	1.5	20.6	16.5	3.2	14.2	1.3	0.3	5.2	2.9	96.9
379	Female	High-middle SDI	2004	0.8	20.9	0.7	20.2	7.1	1.8	28.2	3.0	0.5	4.0	1.2	67.5
380	Female	High SDI	2004	0.9	22.3	1.4	20.9	16.3	3.2	14.5	1.4	0.3	5.1	2.9	96.4
381	Female	Low-middle SDI	2004	3.4	93.3	1.4	91.9	11.1	4.6	64.0	8.4	0.7	2.4	-2.0	9.8
382	Female	Middle SDI	2005	1.3	33.1	0.8	32.4	6.7	2.2	42.4	5.0	0.6	3.1	-0.3	40.7
383	Female	Middle SDI	2004	1.3	33.3	0.8	32.6	6.6	2.1	43.1	5.1	0.6	3.1	-0.3	39.7
384	Female	Low SDI	2004	2.6	70.6	1.0	69.6	7.5	3.3	67.2	9.4	0.8	2.2	-2.4	2.6
385	Female	Low SDI	2007	2.5	67.2	1.0	66.2	7.4	3.3	65.7	9.1	0.8	2.3	-2.3	4.3
386	Female	High-middle SDI	2006	0.8	20.6	0.7	19.8	7.5	1.8	26.5	2.7	0.4	4.1	1.4	70.9
387	Female	High SDI	2006	0.9	22.0	1.5	20.5	16.7	3.2	14.0	1.3	0.3	5.2	2.9	97.3
388	Female	Low-middle SDI	2006	3.4	92.6	1.5	91.2	11.3	4.6	62.8	8.2	0.7	2.4	-1.9	11.5
389	Female	Middle SDI	2007	1.3	32.4	0.8	31.6	6.9	2.2	41.0	4.7	0.6	3.2	-0.1	43.2
390	Female	High-middle SDI	2005	0.8	20.9	0.7	20.1	7.3	1.8	27.3	2.8	0.5	4.1	1.3	69.2
391	Female	Low SDI	2006	2.6	68.8	1.0	67.7	7.4	3.3	66.3	9.2	0.8	2.3	-2.4	3.6
392	Female	High-middle SDI	2008	0.8	20.5	0.8	19.7	7.9	1.9	25.4	2.6	0.4	4.2	1.6	73.1
393	Female	Middle SDI	2006	1.3	32.8	0.8	32.0	6.8	2.2	41.7	4.8	0.6	3.1	-0.2	41.9

394	Female	High SDI	2008	0.9	22.0	1.5	20.5	17.2	3.3	13.5	1.3	0.3	5.2	3.0	98.1
395	Female	Low-middle SDI	2008	3.4	91.0	1.5	89.5	11.5	4.6	61.3	7.9	0.7	2.5	-1.8	13.8
396	Female	Low SDI	2008	2.5	66.4	1.0	65.4	7.4	3.2	65.2	9.0	0.8	2.3	-2.3	5.2
397	Female	Middle SDI	2008	1.3	32.1	0.8	31.3	7.0	2.2	40.3	4.6	0.6	3.2	-0.1	44.6
398	Female	High-middle SDI	2010	0.8	20.4	0.8	19.6	8.3	1.9	24.4	2.5	0.4	4.3	1.7	75.2
399	Female	Low-middle SDI	2007	3.4	92.0	1.5	90.5	11.4	4.6	62.2	8.1	0.7	2.5	-1.9	12.5
400	Female	High SDI	2010	0.9	21.8	1.5	20.3	17.6	3.4	13.2	1.2	0.3	5.2	3.0	98.7
401	Female	Low-middle SDI	2010	3.3	91.1	1.5	89.6	12.1	4.7	59.7	7.6	0.7	2.6	-1.6	16.9
402	Female	High-middle SDI	2007	0.8	20.6	0.8	19.9	7.7	1.9	25.9	2.7	0.4	4.2	1.5	72.2
403	Female	High-middle SDI	2009	0.8	20.5	0.8	19.7	8.1	1.9	24.9	2.5	0.4	4.2	1.6	74.1
404	Female	High SDI	2009	0.9	21.8	1.5	20.3	17.3	3.3	13.4	1.3	0.3	5.2	3.0	98.4
405	Female	Middle SDI	2010	1.3	32.0	0.8	31.2	7.3	2.2	38.8	4.4	0.6	3.3	0.1	47.4
406	Female	Low SDI	2010	2.5	66.0	1.0	65.0	7.6	3.3	63.6	8.7	0.8	2.3	-2.2	7.4
407	Female	High SDI	2007	0.9	22.0	1.5	20.5	17.0	3.3	13.7	1.3	0.3	5.2	3.0	97.7
408	Female	Low-middle SDI	2012	3.4	92.3	1.6	90.8	12.7	4.8	58.5	7.3	0.7	2.6	-1.5	19.0
409	Female	Low SDI	2012	2.6	66.9	1.1	65.9	7.9	3.4	62.2	8.5	0.8	2.3	-2.1	9.2
410	Female	High SDI	2012	0.9	21.7	1.5	20.1	17.6	3.4	13.1	1.2	0.3	5.2	3.0	98.9
411	Female	Low-middle SDI	2009	3.3	90.7	1.5	89.2	11.7	4.6	60.5	7.7	0.7	2.5	-1.7	15.4
412	Female	Middle SDI	2009	1.3	32.0	0.8	31.2	7.1	2.2	39.6	4.5	0.6	3.3	0.0	45.9
413	Female	Low SDI	2009	2.5	66.4	1.0	65.4	7.5	3.3	64.5	8.9	0.8	2.3	-2.2	6.2
414	Female	Low-middle SDI	2011	3.4	91.7	1.5	90.2	12.4	4.8	59.1	7.4	0.7	2.6	-1.6	18.0
415	Female	High-middle SDI	2011	0.8	20.3	0.8	19.5	8.4	1.9	23.9	2.4	0.4	4.3	1.7	76.0
416	Female	Middle SDI	2012	1.3	31.8	0.8	31.0	7.6	2.3	37.5	4.2	0.6	3.4	0.2	49.8
417	Female	High SDI	2011	0.9	21.8	1.5	20.2	17.6	3.4	13.1	1.2	0.3	5.2	3.0	98.8
418	Female	High-middle SDI	2013	0.8	19.8	0.8	19.0	8.6	2.0	22.9	2.3	0.4	4.4	1.8	78.1
419	Female	Low-middle SDI	2013	3.5	93.9	1.6	92.3	13.0	4.9	58.0	7.2	0.7	2.6	-1.5	19.7
420	Female	High SDI	2013	0.9	21.8	1.6	20.2	17.8	3.4	13.0	1.2	0.3	5.2	3.0	99.1
421	Female	Middle SDI	2011	1.3	32.0	0.8	31.2	7.5	2.2	38.1	4.3	0.6	3.3	0.2	48.6
422	Female	Middle SDI	2014	1.3	31.8	0.8	31.0	8.0	2.3	36.5	4.0	0.6	3.5	0.4	52.2
423	Female	Low SDI	2011	2.5	66.2	1.0	65.1	7.7	3.3	62.9	8.6	0.8	2.3	-2.1	8.4
424	Female	Low SDI	2013	2.6	67.8	1.1	66.7	8.1	3.4	61.7	8.4	0.8	2.4	-2.0	9.7
425	Female	High-middle SDI	2012	0.8	20.1	0.8	19.3	8.5	2.0	23.5	2.4	0.4	4.3	1.8	76.9
426	Female	Middle SDI	2013	1.3	31.8	0.8	31.0	7.8	2.3	37.0	4.1	0.6	3.4	0.3	51.0
427	Female	Low-middle SDI	2015	3.6	96.4	1.7	94.7	13.7	5.1	57.0	7.0	0.7	2.7	-1.4	21.3
428	Female	High-middle SDI	2015	0.8	19.9	0.9	19.1	9.0	2.0	22.2	2.2	0.4	4.5	1.9	79.6
429	Female	High-middle SDI	2014	0.8	19.8	0.8	19.0	8.8	2.0	22.6	2.3	0.4	4.4	1.9	79.0
430	Female	Low SDI	2015	2.7	69.8	1.1	68.6	8.6	3.6	60.9	8.2	0.8	2.4	-1.9	11.3
431	Female	High SDI	2015	0.9	21.9	1.6	20.3	18.2	3.5	12.8	1.2	0.3	5.2	3.0	99.4
432	Female	High SDI	2014	0.9	21.7	1.6	20.2	17.9	3.4	12.9	1.2	0.3	5.2	3.0	99.1
433	Female	Middle SDI	2015	1.3	32.1	0.9	31.3	8.2	2.3	36.0	3.9	0.6	3.5	0.4	53.2
434	Female	Low-middle SDI	2014	3.5	94.3	1.6	92.7	13.2	5.0	57.6	7.1	0.7	2.7	-1.4	20.3
435	Female	High-middle SDI	2017	0.8	20.5	0.9	19.6	9.6	2.1	21.6	2.1	0.4	4.5	2.0	81.3
436	Female	Low SDI	2017	2.7	70.6	1.2	69.5	8.9	3.7	59.5	7.9	0.7	2.4	-1.8	13.6
437	Female	Low SDI	2016	2.7	70.0	1.1	68.8	8.7	3.6	60.3	8.0	0.7	2.4	-1.9	12.5

438	Female	Low-middle SDI	2016	3.6	97.6	1.7	95.9	14.1	5.3	56.5	6.9	0.7	2.7	-1.3	22.4
439	Female	High SDI	2017	0.9	22.0	1.6	20.4	18.5	3.5	12.7	1.2	0.3	5.2	3.1	99.8
440	Female	Low-middle SDI	2017	3.7	99.0	1.7	97.2	14.5	5.4	55.8	6.8	0.7	2.7	-1.2	23.5
441	Female	High-middle SDI	2016	0.8	20.2	0.9	19.3	9.3	2.1	21.9	2.2	0.4	4.5	2.0	80.3
442	Female	Middle SDI	2017	1.3	32.7	0.9	31.8	8.7	2.4	34.9	3.8	0.5	3.6	0.6	55.3
443	Female	Low SDI	2014	2.7	69.0	1.1	67.9	8.3	3.5	61.5	8.3	0.8	2.4	-2.0	10.2
444	Female	High SDI	2016	0.9	22.3	1.6	20.7	18.7	3.6	12.7	1.2	0.3	5.2	3.1	99.7
445	Female	Low-middle SDI	2018	3.7	99.6	1.8	97.8	14.9	5.5	55.2	6.7	0.7	2.7	-1.2	24.5
446	Female	High-middle SDI	2018	0.8	20.6	0.9	19.7	9.9	2.2	21.3	2.1	0.4	4.6	2.1	82.1
447	Female	High SDI	2018	0.9	21.8	1.6	20.2	18.4	3.5	12.7	1.2	0.3	5.3	3.1	99.8
448	Female	Middle SDI	2016	1.3	32.4	0.9	31.5	8.4	2.4	35.5	3.9	0.5	3.5	0.5	54.1
449	Female	High SDI	2021	0.9	20.9	1.5	19.4	17.8	3.4	12.5	1.2	0.3	5.3	3.1	100.0
450	Female	Low-middle SDI	2021	3.6	97.9	1.8	96.1	15.5	5.5	52.9	6.3	0.7	2.8	-1.0	28.4
451	Female	Low SDI	2021	2.7	70.1	1.2	68.9	9.6	3.8	57.0	7.3	0.7	2.5	-1.5	18.5
452	Female	Middle SDI	2018	1.3	33.0	0.9	32.1	9.0	2.5	34.4	3.7	0.5	3.6	0.6	56.5
453	Female	Low SDI	2018	2.7	70.7	1.2	69.5	9.1	3.7	59.0	7.8	0.7	2.4	-1.7	14.7
454	Female	High-middle SDI	2019	0.8	20.6	0.9	19.7	10.0	2.2	21.0	2.1	0.4	4.6	2.1	82.6
455	Female	Middle SDI	2021	1.3	33.2	1.0	32.2	9.5	2.6	33.0	3.5	0.5	3.7	0.8	59.4
456	Female	High SDI	2019	0.9	21.7	1.6	20.1	18.4	3.5	12.6	1.2	0.3	5.3	3.1	99.9
457	Female	Low-middle SDI	2019	3.7	99.0	1.8	97.2	15.1	5.5	54.3	6.5	0.7	2.8	-1.1	25.9
458	Female	Low SDI	2019	2.7	70.5	1.2	69.3	9.3	3.7	58.2	7.6	0.7	2.5	-1.7	16.2
459	Female	Middle SDI	2019	1.3	33.2	1.0	32.2	9.2	2.5	33.8	3.6	0.5	3.7	0.7	57.7
460	Female	High-middle SDI	1996	0.9	22.6	0.7	22.0	6.4	1.7	32.3	3.5	0.5	3.7	0.8	59.1
461	Female	High SDI	1996	1.0	24.0	1.4	22.6	15.3	3.2	16.2	1.6	0.3	4.8	2.6	91.6
462	Female	High-middle SDI	2021	0.8	20.3	0.9	19.4	9.9	2.2	20.8	2.0	0.4	4.6	2.1	83.0
463	Female	Low-middle SDI	1996	3.4	93.7	1.4	92.3	10.6	4.5	65.8	8.8	0.8	2.4	-2.2	6.4
464	Female	Middle SDI	2002	1.4	34.4	0.8	33.6	6.6	2.2	44.3	5.2	0.6	3.0	-0.4	38.0
465	Female	Middle SDI	1996	1.5	36.5	0.8	35.7	6.4	2.2	47.0	5.7	0.7	2.9	-0.7	33.1
466	Female	Low SDI	1996	2.6	71.9	1.0	70.9	7.4	3.4	68.4	9.7	0.8	2.2	-2.6	0.1
467	Female	High-middle SDI	1993	0.9	22.8	0.7	22.1	6.1	1.7	33.4	3.7	0.5	3.6	0.6	56.6
468	Female	High SDI	1993	0.9	23.9	1.3	22.6	14.4	3.0	17.1	1.7	0.3	4.8	2.5	90.1
469	Female	Low SDI	1993	2.5	69.2	1.0	68.2	7.1	3.2	68.1	9.7	0.8	2.2	-2.5	0.7
470	Female	High-middle SDI	2002	0.9	21.3	0.7	20.6	6.8	1.7	29.5	3.1	0.5	4.0	1.1	65.0
471	Female	Low-middle SDI	1993	3.2	90.1	1.4	88.7	10.1	4.3	65.6	8.9	0.7	2.3	-2.2	6.8
472	Female	Middle SDI	1993	1.4	36.2	0.7	35.5	6.1	2.1	48.3	5.9	0.7	2.8	-0.8	31.2
473	Female	High SDI	2002	0.9	22.9	1.4	21.5	16.2	3.2	14.9	1.4	0.3	5.1	2.8	95.3
474	Female	Low SDI	2002	2.6	71.6	1.0	70.5	7.5	3.4	67.4	9.5	0.8	2.2	-2.5	1.8
475	Female	Low-middle SDI	2002	3.4	94.4	1.4	93.0	11.0	4.6	64.7	8.6	0.7	2.4	-2.1	8.9
476	Female	Low SDI	2020	2.7	70.2	1.2	69.0	9.5	3.8	57.4	7.4	0.7	2.5	-1.6	17.8
477	Female	Low-middle SDI	2020	3.6	98.4	1.8	96.6	15.4	5.5	53.5	6.4	0.7	2.8	-1.0	27.5
478	Female	High-middle SDI	2020	0.8	20.2	0.9	19.3	9.9	2.1	20.9	2.0	0.4	4.6	2.1	82.7
479	Female	High SDI	2020	0.9	21.0	1.6	19.4	17.9	3.4	12.5	1.2	0.3	5.3	3.1	100.0
480	Female	Middle SDI	2020	1.3	33.1	1.0	32.2	9.4	2.5	33.2	3.5	0.5	3.7	0.8	58.9
